# Modelling the Encapsulation of 3-Hydroxyflavone with Cyclodextrin and Octa Acid and Comparing Their Differences

**DOI:** 10.3390/molecules28093966

**Published:** 2023-05-08

**Authors:** Ka Wa Fan, Hoi Ling Luk, David Lee Phillips

**Affiliations:** 1 Department of Chemistry, University of Hong Kong, Hong Kong 999077, China; u3532679@connect.hku.hk; 2 Guangdong-Hong Kong-Macao Joint Laboratory of Optoelectronic and Magnetic Functional Materials, Hong Kong 999077, China

**Keywords:** 3-hydroxyflavone, cyclodextrin, octa acid

## Abstract

The 3-hydroxyflavone (3-HF) is one of the common fluorescence probes. It has two distinct fluorescence bands: normal form and tautomer form. However, 3-hydroxyflavone has poor performance in water because of hydrogen bonding perturbation. The utilization of supramolecular chemistry would improve the fluorescence performance of 3-hydroxyflavone in water. In this paper, it reviews supramolecular chemistry of 3-hydroxyflavone with cyclodextrin and octa acid. Past research has found that the addition of β-cyclodextrin to 3-hydroxyflavone in water would slightly improve the fluorescence intensity of the tautomer form. When adding γ-cyclodextrin to 3-hydroxyflavone in water, the green fluorescence intensity would be enhanced. Finally, the addition of octa acid creates a dry environment for the 3-hydroxyflavone, and it would only have a tautomer form. The ONIOM calculation shows the ways of self-assembly of β- and γ-cyclodextrin. It can explain the difference in ratio between the tautomer form and normal form after understanding the interaction.

## 1. Introduction

**Scheme 1:**
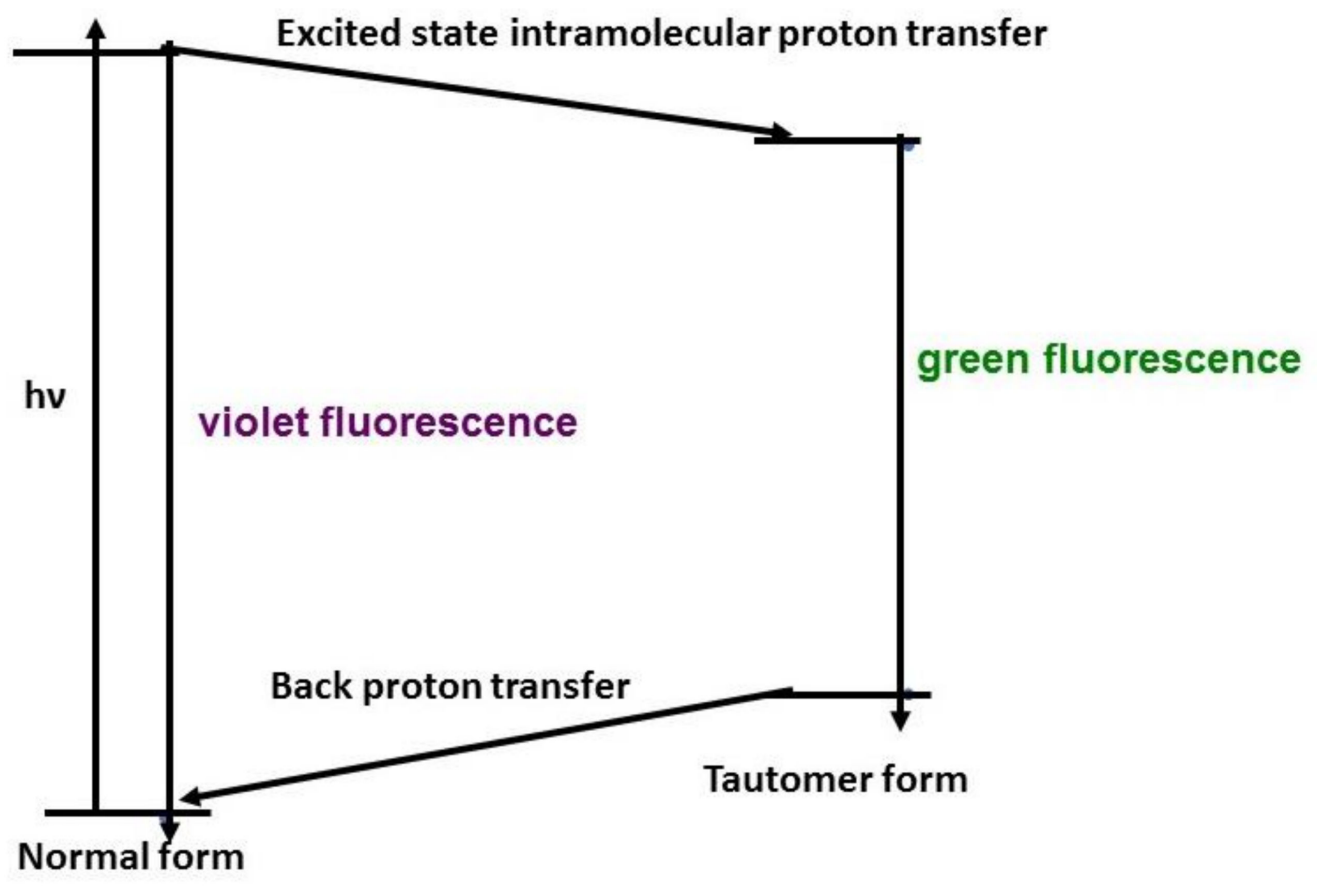
The general mechanism for 3-hydroxyflavone excited state intramolecular proton transfer (ESIPT).

Sengupta and Kasha [1] studied the luminescence of 3-hydroxyflavone and quercetin and proposed the mechanism of excited-state intramolecular proton transfer (ESIPT). The mechanism has been investigated by time-resolved spectroscopy [2,3]. The general mechanism of 3-hydroxyflavone was explained in Scheme 1: The 3-hydroxyflavone would be excited into the higher excited state. Then, the excited 3-hydroxyflavone would undergo vibrational relaxation and internal conversion to the first singlet excited state of the normal form. Next, there are two possible pathways for energy dissipation. The first one would be by giving out a violet fluorescence directly from the normal form. The other pathway would be undergoing tautomerism. The proton is transferred from the hydroxyl group to the carbonyl group. This is because the energy of first singlet excited state of the tautomer form is lower than that of the first singlet excited state of normal form. Later, the tautomer form would relax back to the ground state by giving out a green fluorescence. Since the potential energy surface of the ground state is different from that of first excited singlet state, the energy of the tautomer form on the ground state is higher than that of the normal form and results in back proton transfer to form the normal form.

Klymchenko and Demchenko reported the ratio of intensity of the normal form to the intensity of the tautomer form is 0.312 [4]. The reciprocal ratio would be 3.2 and can be used as a reference point to compare the performance of the different hosts. Therefore, ESIPT is not effective in polar protic solvent [4]. As the hydrogen bond forms between the solvent and 3-hydroxyflavone, it would perturb the tautomerism. Hence, the decay of the excited state would favor the radiative and non-radiative decay of the normal form. To improve the fluorescence intensity of 3-hydroxyflavone in an aqueous medium, different supramolecular cages were added to provide hydrophobic environments.

**Scheme 2:**
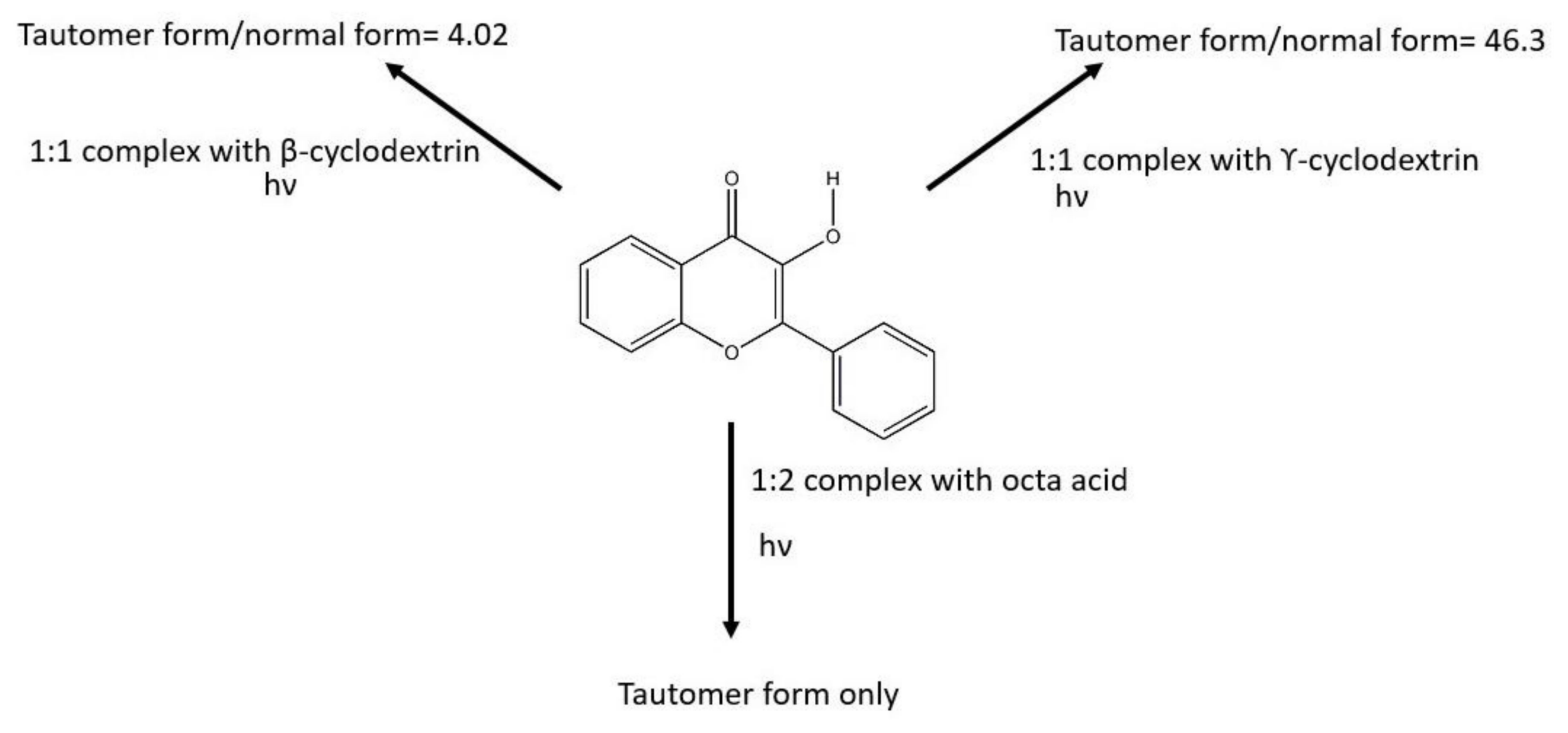
Summary interaction and photophysical properties between 3-hydroxyflavone and different hosts.

The cyclodextrin was a popular host for studying the supramolecular chemistry of different guests [5]. Sengupta and his co-workers studied the encapsulation of 3-hydroxyflavone in β-cyclodextrin [6] and γ-cyclodextrin [7]. In the aqueous medium, they observed the ratio of fluorescence intensity of the tautomer form to its normal form was 4.02 for 3-hydroxyflavone and β-cyclodextrin, as shown in Scheme 2. Then, they also performed similar measurements for 3-hydroxyflavone and γ-cyclodextrin. The result ratio of fluorescence intensity of the tautomer form to its normal form was 46.53. β-cyclodextrin and γ-cyclodextrin is composed of seven and eight glucose subunits, respectively. Since the interior walls of the hosts have a similar environment and same guests are employed for the supramolecular system, one would expect the fluorescence intensity ratios of these two supramolecular complexes give similar results; however, Sengupta group reported a huge difference in the ratio of fluorescence intensity between these two complexes. Recently, the theoretical calculations based on the inclusion complex were further published [8,9]. It is interesting for us to further investigate the self-assembly of 3-hydroxyflavone with β-cyclodextrin and γ-cyclodextrin.

Another supramolecular host that has been experimentally studied would be the octa acid capsule [10]. Octa acid is also an internal hydrophobic capsule that is 1 nm in width and depth. The four external carboxylic acid groups are outside the capsule to improve its solubility in water. Ramamurthy et al. studied the self-assembly between 3-hydroxyflavone and octa acid [11]. They determined the stoichiometry of octa acid and 3-hydroxyflavone was a 2:1 host/guest complex by NMR titration experiment. They studied the fluorescence measurement by the addition of octa acid to 3-hydroxyflavone in an aqueous medium, and they reported only a green fluorescence could be observed. All these results in different hosts are summarized in Scheme 2.

In this paper, our own N-layered integrated molecular orbital and molecular mechanics (ONIOM) is used to model these three complexes [12]. As the host has many atoms, it would be very computationally expensive if a full quantum mechanics calculation, such as density functional theory (DFT) calculation, were employed. Therefore, the treatment of the host was molecular mechanics. Then, the 3-hydroxyflavone part in these complexes would be treated as the DFT part. The theoretical binding energies are compared to the experimental results to deduce the ways of self-assembly. After understanding the ways of self-assembly, one can explain the trend of the ratio tautomer form to the normal form among the three complexes.

## 2. Results

### 2.1. Definition

**Figure 1:**
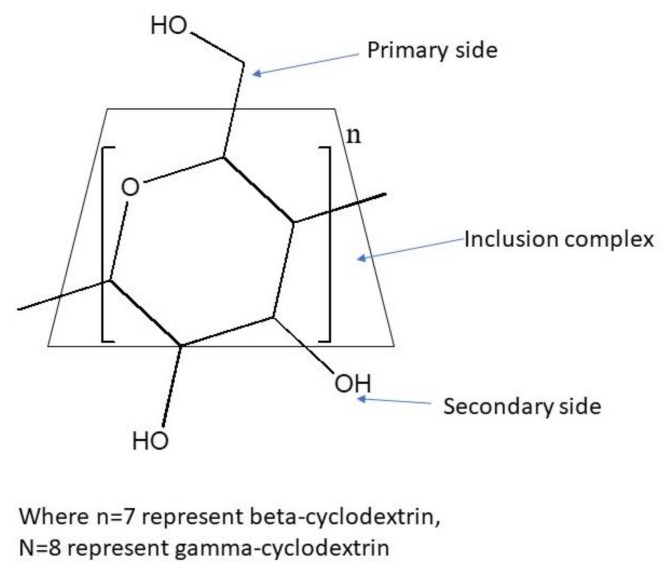
The structure of cyclodextrin.

**Figure 2:**
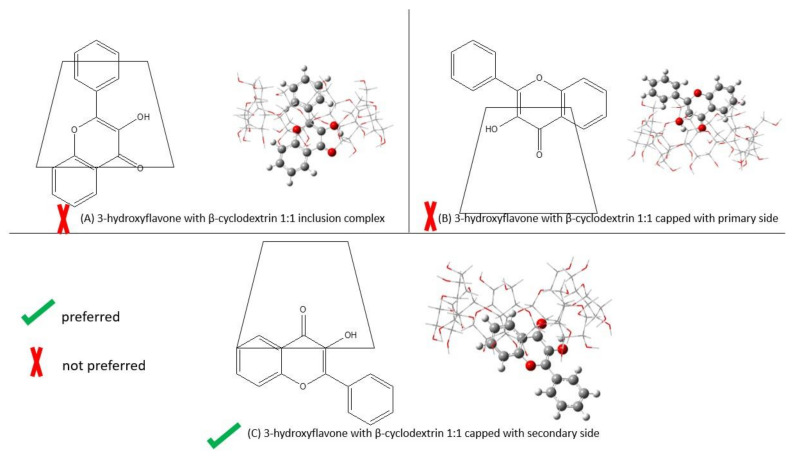
The ways of binding between 3-hydroxyflavone and β-cyclodextrin by B3LYP/6-311+g(d): UFF. The left shows 2-D self-assembly, and the right refers to the optimized structure. (**A**) 1:1 inclusion complex; (**B**) 1:1 capped with primary side; (**C**) 1:1 capped with secondary side.

**Figure 3:**
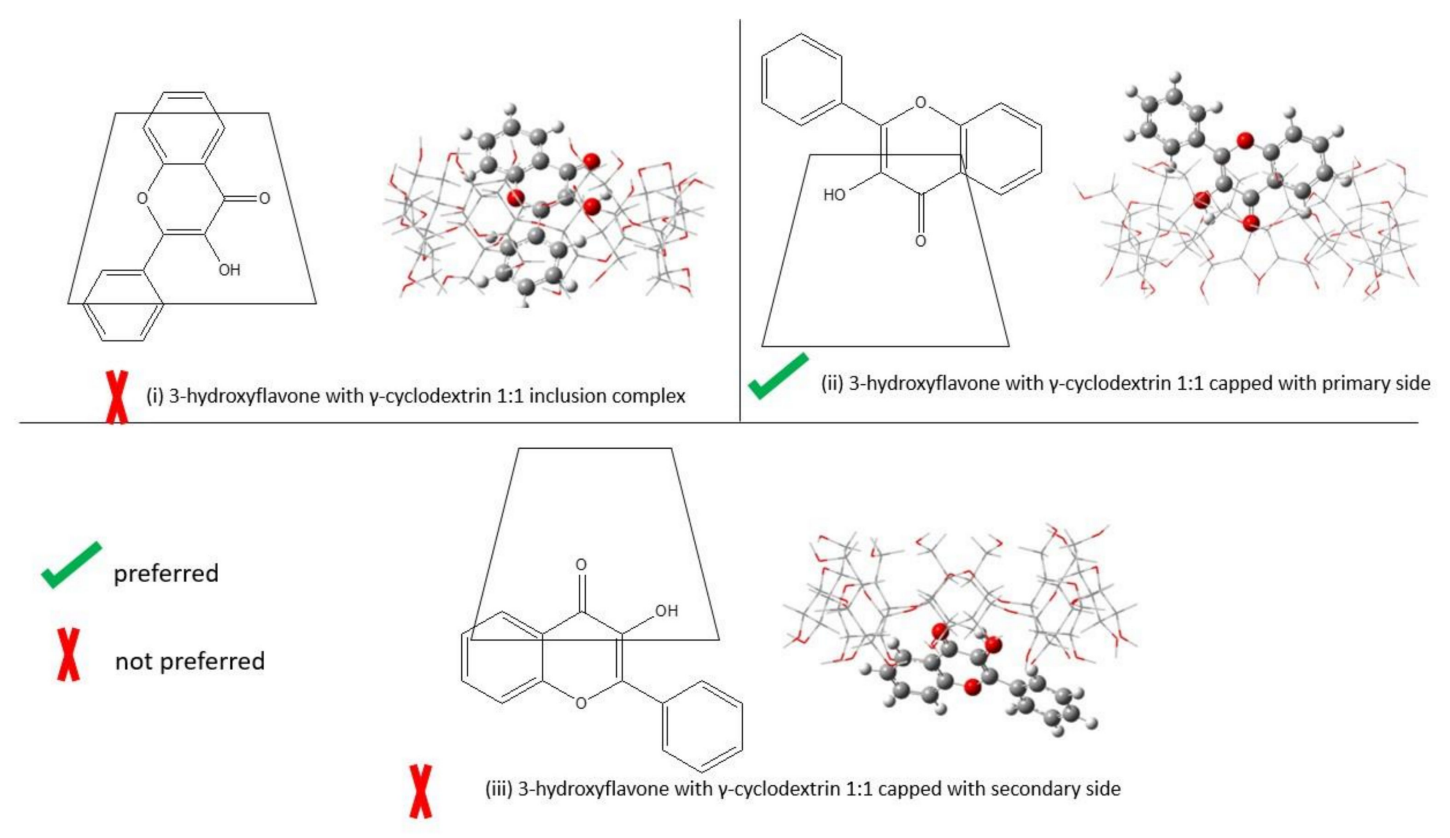
The ways of binding between 3-hydroxyflavone and γ-cyclodextrin by B3LYP/6-311+g(d): UFF. The left shows the 2-D of self-assembly, and the right refers to the optimized structure. (**i**) 1:1 inclusion complex; (**ii**) 1:1 capped with primary side; (**iii**) 1:1 capped with secondary side.

Cyclodextrin is commonly known as a cyclic oligosaccharides, which have a hydrophobic interior and hydrophilic exterior [13]. Since cyclodextrin has a toroidal shapes with the smaller openings (primary side) and larger openings (secondary side), there are three possible ways of interactions formed between 3-hydroxyflavone and cyclodextrin. They are (1) the inclusion complex, in which the 3-hydroxyflavone is completely surrounded by the cyclodextrin; (2) the complex is capped on the primary side; and (3) the complex is capped on the secondary side. In the following representation, we make use of the trapezoid to represent the cyclodextrin (Figure 1). We would have three drawings in Figure 2 and Figure 3. (1) The inclusion complex is represented by the 3-hydroxyflavone and is drawn inside the trapezoid. (2) The 3-hydroxyflavone is drawn close to the upper base of the trapezoid and represents that the complex is capped on the primary side. (3) The 3-hydroxyflavone is drawn close to the lower base and represents that the complex is capped on the secondary side.

As the primary side and secondary side of cyclodextrin have different functional groups attached, the 3-hydroxyflavone capped on each side should be considered as a different interaction. Therefore, we need to specify that it is capped on the different sides as two interactions. As was found in the calculation of [8], the orientation of 3-hydroxyflavone in the inclusion complex is not too different. For simplicity we only present one structure in our calculation. Please refer to the XYZ coordinate in the Supplementary Materials for exact structure. At the same time, the capped complex should be the carbonyl group pointing towards the cyclodextrin and results in one orientation. Table 1 shows the corresponding Gibbs free energies in gas phase of 3-hydroxyflavone and two different cyclodextrin hosts. 

### 2.2. Ways of Self-Assembly for 3-Hydroxyflavone and β-Cyclodextrin

Table 2 shows the binding energy of the different ways of self-assembly of 3-hydroxyflavone with β-cyclodextrin. The optimized structure of the different ways of self-assembly of 3-hydroxyflavone with β-cyclodextrin is shown in Figure 2. The computed results shows that the inclusion complex has the largest binding energy. Then, the 3-hydroxyflavone capped with the secondary side has the second most stable binding energy. Finally, the 3-hydroxyflavone capped with the primary side is the least stable structure.

When comparing the computed energy with experimental values (−3.67 kcal/mol) [6], the binding energy of 3-hydroxyflavone capped with the secondary side of β-cyclodextrin is preferred since it only has 6% error. The binding energy of the inclusion complex significantly deviates by 201% from the experimental value. Additionally, being capped with the primary side is also not preferred, as it has a −46% error relative to the experimental value.

### 2.3. Ways of Self-Assembly of 3-Hydroxyflavone and γ-Cyclodextrin

Table 3 shows the binding energy of the different ways of self-assembly of 3-hydroxyflavone with γ-cyclodextrin. The optimized structure of the different ways of self-assembly of 3-hydroxyflavone with γ-cyclodextrin is shown in Figure 3. When comparing the binding energies between 3-hydroxyflavone and γ-cyclodextrin, the inclusion complex is still the most stable form. Then, the second most stable binding energy is capped with the primary side. Finally, the least stable binding energy is capped with the secondary side.

Similar to case of β-cyclodextrin, the binding energy of inclusion complex also has an error of 178% compared to the experimental value. This also suggests that the inclusion complex is unlikely to be the binding form in experiment. Hence, the actual binding should be the structures that are capped with one of the sides. The calculation shows that the binding energies of 3-hydroxyflavone with β-cyclodextrin and γ-cyclodextrin are different. 3-hydroxyflavone would prefer to be capped with the primary side of γ-cyclodextrin, as the binding energy is only −6% error.

### 2.4. Variation among Different Computation Methods

Other density functionals and MP2 were used to calculate the binding energies of the three different complexes with β-cyclodextrin and are shown in Table 4. The binding energy of inclusion complex with β-cyclodextrin is around 10.5 kcal/mol by all the methods, which is consistent with the B3LYP. This shows a large discrepancy with the experimental binding energy. The experimental result is better approximated by the binding energy of the structure, which is capped on the secondary side by B97D3/6-311+g(d): UFF. The error in the binding energy of those capped with the secondary side is 2%.

Other density functionals and MP2 were also used to calculate the binding energies of the three different complexes with γ-cyclodextrin and are shown in Table 5. The binding energy of the inclusion complex with γ-cyclodextrin is close to 8.5 kcal/mol by all the method, which is consistent with the B3LYP. Similarly, the estimation of the binding energy of those capped with the primary side by B3PW91/6-311+g(d): UFF is close to the experiment. The error in the binding energy of those capped with the primary side is −2%.

In short, different methods show similar trends, which indicate the structure of 3-hydroxyflavone capped with the secondary side of β-cyclodextrin is preferred, while the structure of 3-hydroxyflavone capped with the primary side of γ-cyclodextrin is preferred.

### 2.5. Supramolecular Chemistry of 3-Hydroxyflavone and Octa Acid

As the 2:1 host/guest complex of 3-hydroxyflavone and octa acid has been determined by an NMR experiment, there is only one way for the binding to occur. 3-hydroxyflavone and 4′-(N,N-Diethylamino)-3-hydroxyflavone with octa acid cavitand were optimized to ground state of two species by B3LYP/6-311+g(d): UFF and the differences in geometry were compared.

**Figure 4:**
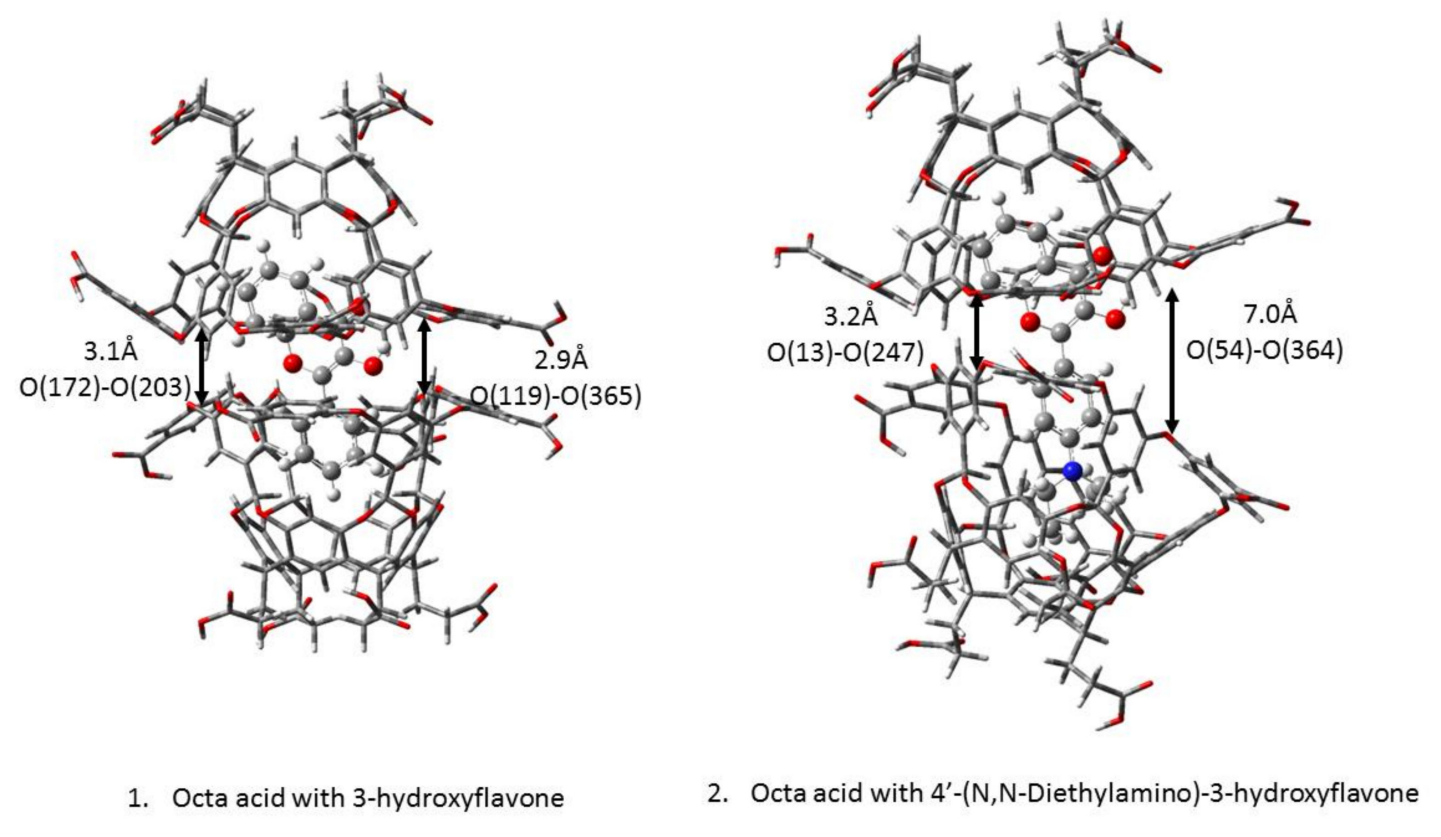
Optimized structure of (**1**) 3-hydroxyflavone with two octa acids (**2**) 4′-(N,N-Diethylamino)-3-hydroxyflavone with two octa acids.

The optimized structures show that the two octa acid cavitands encapsulate the 3-hydroxyflavone completely while the two octa acid cavitands with 4′-(N,N-Diethylamino)-3-hydroxyflavone is exposed to some area to the surroundings. The two optimized structures can be found in Figure 4. The left panel is optimized in a 2:1 host/guest complex of 3-hydroxyflavone and octa acid. When we measure the bond distance between O (172)-O (203) and O (119)-O (365), the two bond distances are close to each other. The right panel is optimized in a 2:1 host/guest complex of 4′-(N,N-Diethylamino)-3-hydroxyflavone and octa acid. When we measure the bond distance between O (13)-O (247) and O (54)-O (364), the two bond distances were different by 3.8 Å.

The structural difference between the two guests would be increased the addition of a bulky group, a N,N-diethylamino substituent at the para position on a phenyl ring. The exposed part would be moiety of the excited state intramolecular proton transfer. In our model, we have not drawn the water box for the calculations; however, when looking at the two octa acids in the two optimized structures, it can deduce that the waterproof quality for 4′-(N,N-Diethylamino)-3-hydroxyflavone is not as good as that of 3-hydroxyflavone. The water molecules would interact with the 4′-(N,N-Diethylamino)-3-hydroxyflavone in an octa acid capsule. Ramamurthy and co-worker reported that 3-hydroxyflavone in octa acid only had green emission and the substituted one had two distinct fluorescence bands [11]. Therefore, it demonstrates that 3-hydroxyflavone in an octa acid capsule can be viewed as dry and a non-polar environment.

## 3. Discussion

### 3.1. Ways of Self-Assembly

Douhal et al. has found that the addition of cyclodextrin to 3-hydroxyflavone in DMF [14] and the addition of γ-cyclodextrin to 4′-(N,N-dimethylamine)-3-hydroxyflavone in DMF [15] would assist the formation of anions. As they measured the absorption of the supramolecular complex, they reported that the intensity of the absorption band at 425 nm, which is the absorption from the anion, increases when the concentration of γ-cyclodextrin in the solution increases. Additionally, the intensity of the absorption band at 340 nm, which is the absorption from normal form, decreases when the concentration of γ-cyclodextrin in the solution increases. The 3-hydroxyflavone in DMF, without the presence of cyclodextrin, had anion formation. Therefore, they tried to add 1.5 M water to the 3-hydroxyflavone in DMF. The absorption band at 425 nm slightly decreased. They explained that the increase of the absorption band at 425 nm is not because of the water released from the host. Finally, they added maltose to the 3-hydroxyflavone in DMF and also observed a small increase in absorption at 425 nm. This experiment explains that the deprotonation of 3-hydroxyflavone is due to its interaction with the hydrogen bonding network. They further extended their studies to the supramolecular chemistry of 4′-(N,N-dimethylamine)-3-hydroxyflavone with γ-cyclodextrin. They proposed the capped complex is one of self-assembly between 4′-(N,N-dimethylamine)-3-hydroxyflavone and γ-cyclodextrin. These results lay an important foundation for us to find out the ways of self-assembly.

First, our calculations predict that the inclusion complex should be viewed as the most stable among the three complexes because the binding energy of the inclusion complex is the largest. This prediction is consistent with most of the observations between cyclodextrin and the different guests; however, the inclusion complex cannot explain the experimental observations. For example, the addition of cyclodextrin to the 3-hydroxyflavone in the buffer and DMF show different absorption and emission spectra [5,6,12]. The inclusion complex model fails to answer the observation of anion formation in DMF, but no anion formation in the buffer. This means that the addition of cyclodextrin does not completely isolate the 3-hydroxyflavone from the bulk solvent. Hence, the 3-hydroxyflavone can still deprotonate by solute and solvent interactions. Therefore, we cannot simply only compare the binding energy but must also compare the experimental free energy to look for the ways of binding. In Table 2 and Table 3, they show that there is an overestimation in the binding energy of the inclusion complex in β- and γ-cyclodextrin by −201% and −178%, respectively [5,6]. Therefore, the 3-hydroxyflavone is unlikely to form the inclusion complex [8].

Then, we compared the experimental binding energy to the theoretical binding energy of the other two complexes; it shows that 3-hydroxyflavone would prefer to be capped on the secondary side of β-cyclodextrin, while would prefer to be capped on the primary side of γ-cyclodextrin. It is because the calculated binding energy of 3-hydroxyflavone are closer to the experimental value. In Table 2 and Table 3, the error in the binding free energy of 3-hydroxyflavone preferring to be capped on the secondary side of β-cyclodextrin and capped on the primary side of γ-cyclodextrin would be 6% and −6%, respectively. This explains why the ratio of the tautomer form to the normal form emission intensity of the two complexes is significantly different. Additionally, the wavelength of the tautomer emission was recorded differently. The tautomer emission for 3-hydroxyflavone with β-cyclodextrin and γ-cyclodextrin were 521.5 nm and 538 nm, respectively. These observations suggest that 3-hydroxyflavone experiences different supramolecular chemistry environments. Because both emission comes from the tautomer of 3-hydroxyflavone, it should be very close if 3-hydroxyflavone were capped on same side of surface for both; however, this was not true in Sengupta’s group work. Therefore, the difference between β-cyclodextrin and γ-cyclodextrin is different by 1 glucose subunit. The size of the secondary side of β-cyclodextrin is close to the size of the primary side of γ-cyclodextrin. The size of the primary side of β-cyclodextrin is much smaller than the size of the secondary size of γ-cyclodextrin. The non-covalent interaction between 3-hydroxyflavone and cyclodextrin should have a similar size fitting. Therefore, the calculations of our models can explicitly demonstrate that 3-hydroxyflavone prefers to be capped on the secondary side of β-cyclodextrin and prefers capped on the primary side of γ-cyclodextrin.

For 3-hydroxyflavone with octa acid, there is only one possible way of self-assembly due to the NMR-measurement-confirmed 2:1 host: guest complex. It was observed that the two capsules close completely from the optimized structure in Figure 4. It also agrees with Ramamurthy’s work on coumarins [16]. The smaller size of the guests can cause them to be completely encapsulated, and no water can interact with the molecules. Due to this, it is known that 3-hydroxyflavone in this complex is a completely dry and non-polar environment.

### 3.2. Ratio of the Tautomer Form to the Normal Form

The excited state of the intramolecular proton transfer of 3-hydroxyflavone is sensitive to the environment [4]. When 3-hydroxyflavone is excited in a more polar solvent, it has a larger resistance to the proton transfer and results in an increase in the intensity of fluorescence from the normal form.

After knowing the ways of self-assembly, one can explain the trend in the ratio of the tautomer form to the normal form. Let it be remembered that, regarding ways of binding, between 3-hydroxyflavone and β-cyclodextrin prefer to be capped on the secondary side. The secondary side of β-cyclodextrin had 14 hydroxyl groups nearby 3-hydroxyflavone. Due to the hydrogen bonding network, it provides the highest activation energy barrier during the excited-state intramolecular proton transfer. Then, it would slightly enhance the formation of the tautomer form and green fluorescence decay so that it only slightly increases the ratio of the tautomer form to the normal form when compared to its aqueous solution.

Regarding the ways of binding, 3-hydroxyflavone and γ-cyclodextrin prefer to be capped on the primary side; the primary side of γ-cyclodextrin is less polar and has eight CH_2_OH groups pointing toward the 3-hydroxyflavone. With such non-covalent interactions, it has a smaller activation energy barrier during the excited-state intramolecular proton transfer. As such, it would be easier for the excited normal form to overcome the energy barrier and green fluorescence decay. Therefore, it has a larger ratio of the tautomer form to the normal form when compared to its buffer solution.

In the optimized structure in Figure 4, which shows the ratio 2:1 host/guest complex of 3-hydroxyflavone, octa acid is completely encapsulated and 3-hydroxyflavone in the interior wall of octa acid experience a dry and non-polar environment. The excited-state intramolecular proton transfer of 3-hydroxyflavone in a non-polar solvent such as benzene would have a low activation barrier. All the excited 3-hydroxyflavone would undergo a proton transfer and decay by fluorescence of the tautomer form. Therefore, the 3-hydroxyflavone with octa acid also exhibits similar characteristics with the non-polar solvent conditions if the complex were to be solvated in buffer conditions). All the excited 3-hydroxyflavone in the interior wall with the two octa acids can overcome the low activation barrier. After the excited-state intramolecular proton transfer, the tautomer form decays by green fluorescence.

In short, the ratio of the tautomer form to the normal form is correlated to the ways of binding. The ways of binding of each host with 3-hydroxyflavone affects the activation energy barrier of the excited-state intramolecular proton transfer. More polar environments given by the host would result in higher activation energy. The higher activation energy would retard the formation of the tautomer form. Finally, the ratio of the tautomer form to the normal form measured by emission would become less.

## 4. Materials and Methods

All the computations were performed using the Gaussian16 suite [17]. The free energies of 3-hydroxyflavone in gas phase was calculated by different density functional theory(DFT)/6-311+g(d), and the host system was calculated by molecular mechanics (UFF). The host and guest complex was calculated using the ONIOM method; DFT/6-311+g(d) was used for 3-hydroxyflavone and UFF for the host part. Then, the binding energy was calculated by the free energy obtained by ONIOM minus the free energy obtained by DFT and the free energy obtained by MM. We take the experimental free energy reported by Sengupta’s group as reference. The error percentage was calculated from the free energy minus the experimental energy and then divided by the experimental free energy. After calculating the error, which ways of binding would be closest to reality can be found out.

The binding energy was calculated as:

$$E_{\mathrm{bindings}}=E_{\mathrm{oniom}}-E_{3-\mathrm{HF}(\mathrm{gas})}-E_{\mathrm{host}(\mathrm{gas})}$$

The error was calculated as:

$$\mathrm{Error}=\frac{E_{\mathrm{computed}}-E_{\mathrm{experiment}}}{E_{\mathrm{experiment}}}\times 100\%$$

## 5. Conclusions

The excited-state intramolecular proton transfer of 3-hydroxyflavone is not effective in a polar solvent due to hydrogen bond perturbation; it would limit the application of 3-hydroxyflavone as a fluorescent probe. It can utilize the supramolecular host to encapsulate 3-hydroxyflavone and can increase the intensity of fluorescence performance of the tautomer.

In the literature, research studies were performed on three supramolecular complexes: (1) the supramolecular chemistry of 3-hydrxyflavone and β-cyclodextrin, (2) the supramolecular chemistry of 3-hydrxyflavone and γ-cyclodextrin, and (3) the supramolecular chemistry of 3-hydrxyflavone and octa acid. These three supramolecular hosts show how to improve the fluorescence performance to different extents. The supramolecular chemistry of 3-hydroxyflavone and β-cyclodextrin complex slightly increases the intensity of fluorescence when compared to 3-hydroxyflavone in a buffer solution. The supramolecular chemistry of 3-hydroxyflavone and γ-cyclodextrin complex had a better increase in the fluorescence intensity relative to supramolecular chemistry of 3-hydroxyflavone and β-cyclodextrin. The supramolecular chemistry of 3-hydroxyflavone and octa acid allowed for a complete proton transfer.

To explain this trend, we start with the discussion of the ways of self-assembly of the 3-hydroxyflavone with different host. There are a total of three possible ways of self-assembly of 3-hydroxyflavone with cyclodextrin: (1) the inclusion complex, in which 3-hydroxyflavone inside the cyclodextrin; (2) the complex is capped on the primary side of cyclodextrin, which means that 3-hydroxyflavone interacts with the smaller opening of the host; and (3) the complex is capped on the secondary side of cyclodextrin, which means that 3-hydroxyflavone interacts with the larger opening of the host. By comparing the theoretical binding energy and experimental binding energy, we prove that 3-hydroxyflavone prefers to be capped on the secondary side of β-cyclodextrin and prefers to be capped on the primary side of γ-cyclodextrin. Moreover, once the ways of binding of 3-hydroxyflavone and cyclodextrin are known, it can also explain the formation of anions in the DMF. As for the octa acid, it can confirm the ways of self-assembly are a 2:1 host/guest complex. Therefore, it only has a single way of binding, in which the two octa acids completely close in structure by ONIOM optimization.

So, the extent of the improved fluorescence performance of the tautomer form can be related with the environment of the host provided. As 3-hydroxyflavone prefers to be capped on the secondary side of β-cyclodextrin, the excited normal form of 3-hydroxyflavone has the highest activation energy of proton transfer. As a result, the intensity of fluorescence of the tautomer form slightly enhanced. Furthermore, 3-hydroxyflavone prefers to be capped on the primary side of γ-cyclodextrin, as it would have fewer polar CH_2_OH groups pointing to 3-hydroxyflavone. Hence, it would have less activation energy for the proton transfer and more tautomer forms can be formed; therefore, more emissions can come from tautomers. The two octa acids provide dry and non-polar environment to 3-hydroxyflavone. Thus, the activation energy for 3-hydroxyflavone is the lowest. All the excited normal forms can pass through the energy barrier and be converted to the tautomer form. In this way, the reason why only green emissions can be observed for 3-hydroxyflavone with an octa acid complex can be explained.

## Figures and Tables

**Table 1:** Gibbs free energies in gas phase.

	Method	Gibbs Free Energies (Hartree)
3-hydroxyflavone	B3LYP/6-311+g(d)	−803.316151
β-cyclodextrin	UFF	1.595391
ϒ-cyclodextrin	UFF	1.816360

**Table 2:** Gibbs free energies of the 3-HF and β-CD complex that was computed by B3LYP/6-311+g(d): UFF.

Ways of Binding	E_oniom_ (Hartree)	E_binding_ (kcal/mol)	Error ^1^ (%)
Inclusion	−801.73836	−11.04	201
Capped with primary side	−801.723895	−1.97	−46
Capped with secondary side	−801.726953	−3.89	6

^1^ The experimental value would be −3.67 kcal/mol [6].

**Table 3:** Gibbs free energies of the 3-HF and γ-CD complex that was computed by B3LYP/6-311+g(d): UFF.

Ways of Binding	E_oniom_ (Hartree)	E_binding_ (kcal/mol)	Error ^1^ (%)
Inclusion	−801.513753	−8.76	178
Capped with primary side	−801.504527	−2.97	−6
Capped with secondary side	−801.502545	−1.73	−45

^1^ The experimental value is −3.157 kcal/mol [7].

**Table 4:** Gibbs free energies of the 3-HF and β-CD complex and the corresponding errors from experimental values by different methods.

Method/6-311+g(d)	Inclusion	Capped with Primary Side	Capped with Secondary Side
B3PW91	−10.94	−2.04	−4.09
	198%	−44%	11%
B97D	−10.61	−1.83	−4.00
	189%	−50%	9%
B97D3	−10.55	−1.73	−3.73
	187%	−53%	2%
BPW91	−10.66	−1.78	−3.91
	190%	−52%	6%
Cam-B3LYP	−10.85	−1.96	−3.96
	196%	−47%	8%
M06	−10.98	−2.10	−4.04
	199%	−43%	10%
MP2	−10.28	−2.02	−4.03
	180%	−45%	10%
PBEPBE	−10.72	−1.81	−3.93
	192%	−51%	7%
wB97XD	−10.73	−2.23	−3.87
	192%	−39%	5%

**Table 5:** Gibbs free energies of the 3-HF and γ-CD and the corresponding errors from experimental values by different methods.

Method/6-311+g(d)	Inclusion	Capped with Primary Side	Capped with Secondary Side
B3PW91	−8.89	−3.08	−1.80
	182%	−2%	−43%
B97D	−8.75	−3.02	−1.95
	177%	−4%	−38%
B97D3	−8.55	−2.77	−1.60
	171%	−12%	−49%
BPW91	−8.67	−2.90	−1.61
	175%	−8%	−49%
Cam-B3LYP	−8.79	−2.98	−1.69
	179%	−6%	−46%
M06	−8.99	−3.05	−1.70
	185%	−3%	−46%
MP2	−8.05	−3.31	−1.54
	155%	5%	−51%
PBEPBE	−8.69	−2.91	−1.67
	175%	−8%	−47%
wB97XD	−8.64	−3.00	−1.66
	174%	−5%	−47%

## Data Availability

Not applicable.

## Supplementary Materials

The following supporting information can be downloaded at: https://www.mdpi.com/article/10.3390/molecules28093966/s1. It is provided Gibbs free energy and XYZ coordinate.

## References

[1] Sengupta P.K., Kasha M. (1979). Excited state proton-transfer spectroscopy of 3-hydroxyflavone and quercetin. Chem. Phys. Lett..

[2] Itoh M., Tokumura K., Tanimoto Y., Okada Y., Takeuchi H., Obi K., Tanaka I. (1982). Time-Resolved and Steady-State Fluorescence Studies of the Excited-State Proton Transfer in 3-Hydroxyflavone and 3-Hydroxychromone. J. Am. Chem. Soc..

[3] Itoh M., Tanimoto Y., Tokumura K. (1983). Transient Absorption Study of the Intramolecular Excited-State and Ground-State Proton Transfer in 3-Hydroxyflavone and 3-Hydroxychromone. J. Am. Chem. Soc..

[4] Klymchenko A.S., Demchenko A.P. (2004). 3-Hydroxychromone dyes exhibiting excited-state intramolecular proton transfer in water with efficient two-band fluorescence. New J. Chem..

[5] Lipkowitz K.B. (1998). Applications of computational chemistry to the study of cyclodextrins. Chem. Rev..

[6] Banerjee A., Sengupta P.K. (2006). Encapsulation of 3-hydroxyflavone and fisetin in β-cyclodextrins: Excited state proton transfer fluorescence and molecular mechanics studies. Chem. Phys. Lett..

[7] Pahari B., Chakraborty S., Sengupta P.K. (2011). Encapsulation of 3-hydroxyflavone in γ-cyclodextrin nanocavities: Excited state proton transfer fluorescence and molecular docking studies. J. Mol. Struct..

[8] Kerdpol K., Daengngern R., Sattayanon C., Namuangruk S., Rungrotmongkol T., Wolschann P., Kungwan N., Hannongbua S. (2021). Effect of water microsolvation on the excited-state proton transfer of 3-hydroxyflavone enclosed in -cyclodextrin. Molecules.

[9] Praveena A., Prabu S., Madi F., Rajamohan R. (2022). Theoretical Investigation of Inclusion Complexes of 3-Hydroxyflavone and Quercetin as Guests with Native and Modified β-Cyclodextrins as Hosts. Polycycl. Aromat. Compd..

[10] Gibb C.L.D., Gibb B.C. (2004). Well-Defined, Organic Nanoenvironments in Water: The Hydrophobic Effect Drives a Capsular Assembly. J. Am. Chem. Soc..

[11] Santos F.S.R.F.S., Ramasamy E., Ramamurthy V. (2014). Excited State Chemistry of Flavone Derivatives in a Confined Medium: ESIPT emission in aqueous media. Photochem. Photobiol. Sci..

[12] Chung L.W., Sameera W.M.C., Ramozzi R., Page A.J., Hatanaka M., Petrova G.P., Harris T.V., Li X., Ke Z., Liu F. (2015). The ONIOM Method and Its Applications. Chem. Rev..

[13] Poulson B.G., Alsulami Q.A., Sharfalddin A., El Agammy E.F., Mouffouk F., Emwas A.-H., Jaremko L., Jaremko M. (2021). Cyclodextrins: Structural, Chemical, and Physical Properties, and Applications. Polysaccharides.

[14] Tormo L., Douhal A. (2005). Caging anionic structure of a proton transfer dye in a hydrophobic nanocavity with a cooperative H-bonding. J. Photochem. Photobiol. A Chem..

[15] Organero J.A., Tormo L., Sanz M., Roshal A., Douhal A. (2007). Complexation effect of γ-cyclodextrin on a hydroxyflavone derivative: Formation of excluded and included anions. J. Photochem. Photobiol. A Chem..

[16] Das A., Sharma G., Kamatham N., Prabhakar R., Sen P., Ramamurthy V. (2019). Ultrafast Solvation Dynamics Reveal that Octa Acid Capsule’s Interior Dryness Depends on the Guest. J. Phys. Chem. A.

[17] Frisch M.J., Trucks G.W., Schlegel H.B., Scuseria G.E., Robb M.A., Cheeseman J.R., Scalmani G., Barone V., Petersson G.A., Nakatsuji H. (2016). Gaussian 16, Revision B.01.

