# Supplementary material for: Modelling the Encapsulation of 3-Hydroxyflavone with Cyclodextrin and Octa Acid and Comparing Their Differences
Source: Molecules. 2023 May 8;28(9):3966. doi: 10.3390/molecules28093966 (PMC10179834; doi:10.3390/molecules28093966)
Supplement: Supplementary file 1 [file molecules-28-03966-s001.zip › molecules-2327253-supplementary.pdf]

Supporting information

It is provided Gibbs free energy and XYZ coordinate

Contents

3-hydroxyflavone 2
β-cyclodextrin 3
ϒ-cyclodextrin 7
Octa acid cavitand 11
3-hydroxyflavone with β-cyclodextrin 1:1 inclusion complex 21
3-hydroxyflavone with β-cyclodextrin 1:1 Capped with primary side 26
3-hydroxyflavone with β-cyclodextrin 1:1 Capped with secondary side 31
3-hydroxyflavone with ϒ-cyclodextrin 1:1 inclusion complex 36
3-hydroxyflavone with ϒ-cyclodextrin 1:1 Capped with primary side 42
3-hydroxyflavone with ϒ-cyclodextrin 1:1 Capped with secondary side 47
3-hydroxyflavone with octa acid cavitand 1:2 inclusion complex 52
4'-(N,N-Diethylamino)-3-hydroxyflavone with octa acid cavitand 1:2 inclusion complex 64
3-hydroxyflavone with the water box .... **Error! Bookmark not defined.**

3-hydroxyflavone

Sum of electronic and thermal Free Energies= -803.316151 (dft)

```
C 1.58217000 -0.87574300 0.00002400
C 2.27616200 -2.09109200 0.00005700
C 3.65993300 -2.07108700 0.00005600
C 4.36361800 -0.85328600 0.00002300
C 3.67153000 0.34211400 -0.00001000
C 2.26444700 0.34710300 -0.00001000
H 1.71894900 -3.02078300 0.00008300
H 4.20537300 -3.00893700 0.00008200
H 5.44800300 -0.85629400 0.00002200
H 4.18454700 1.29693800 -0.00003700
C 1.48566600 1.57680700 -0.00004300
C 0.03545400 1.41181400 -0.00003700
C -0.56206400 0.18188900 -0.00000300
C -1.99377500 -0.14093100 0.00000400
C -2.40575100 -1.48759300 0.00001300
C -2.98331200 0.86072500 0.00000400
C -3.75518100 -1.81865500 0.00002100
C -4.33169100 0.51789300 0.00001200
H -2.69659300 1.90132200 -0.00000300
C -4.72657500 -0.81830700 0.00002100
H -4.04886500 -2.86340200 0.00002700
H -5.07828500 1.30541100 0.00001200
H -5.78010300 -1.07844400 0.00002700
O 1.95409300 2.72197800 -0.00007400
O -0.65529000 2.57491400 -0.00006900
H 0.03071800 3.27323900 -0.00009100
O 0.22570100 -0.94244500 0.00002700
H -1.66354600 -2.27453300 0.00001400
```

β-cyclodextrin

Sum of electronic and thermal Free Energies= 1.595391 (uff)

O-O_3 2.82014900 4.10229200 -2.61891700

H-H_ 2.33622600 3.55722300 -3.29293700

O-O_3 5.50705000 4.09625300 -1.77165600

H-H_ 5.89868600 4.97797900 -1.53751400

O-O_3 6.46658200 1.40335700 -2.01003000

H-H_ 6.17862500 1.75204200 -2.89390700

O-O_3 5.52663300 -1.38860700 -2.85361100

H-H_ 4.79193000 -0.72592500 -2.77440200

O-O_3 3.83462600 -3.70809300 -2.64434600

H-H_ 3.52049900 -3.12340500 -3.38259100

O-O_3 2.71577600 -6.24119300 -1.81074000

H-H_ 3.46765900 -6.83948900 -1.56088800

O-O_3 -0.13699100 -6.47820900 -2.03312500

H-H_ 0.27168700 -6.32149900 -2.92427400

O-O_3 -2.63306700 -4.98529800 -2.90781600

H-H_ -1.85180300 -4.37528100 -2.85392300

O-O_3 -4.63546500 -2.93126800 -2.76538500

H-H_ -4.01229200 -2.74293300 -3.51500400

O-O_3 -7.11764600 -0.97704900 -1.77554900

H-H_ -6.30272700 -1.10076000 -2.32527600

O-O_3 -6.70770900 1.88974400 -1.66920000

H-H_ -6.70586200 1.57394100 -2.61038900

O-O_3 -5.20331000 4.41172700 -2.00772700

H-H_ -5.31757200 5.19133500 -2.61152500

O-O_3 -2.65647100 5.76047600 -2.24594700

H-H_ -2.86386100 5.34469600 -3.12328400

O-O_3 0.16947600 5.14662500 -2.98742200

H-H_ -0.29412700 4.28793900 -2.80537900

O-O_3 -0.73939000 4.69951000 -0.30339900

O-O_3 3.40749200 3.43452300 0.11366600

O-O_3 4.94401400 -0.37623500 -0.22382300

O-O_3 2.68909900 -4.01285200 -0.01726800

O-O_3 -1.49134400 -4.53862200 -0.31514300

O-O_3 -4.75380600 -1.62977600 -0.21434100

O-O_3 -4.42666000 2.59672100 -0.00471200

O-O_3 0.93439500 6.14235400 0.45214800

O-O_3 5.08214300 2.80339600 1.59929800

O-O_3 5.88985800 -2.31761700 0.66195600

O-O_3 1.57877500 -5.34897100 1.53129500

O-O_3 -3.56923800 -5.03397000 0.62619100

O-O_3 -5.73195900 -0.46217100 1.55300200

O-O_3 -3.97334500 4.40118300 1.41120800

O-O_3 -2.13258200 5.97571400 2.82065000

H-H_ -1.62273500 6.16576400 3.65060200

O-O_3 3.37260900 6.54763600 1.89762200

H-H_ 2.81210600 7.36637000 1.86984100

O-O_3 6.41705800 0.80238200 3.00628800

H-H_ 6.54181100 0.25987700 3.82785700

O-O_3 5.57474300 -4.75652800 2.06103700

H-H_ 5.57918800 -5.24446700 2.92508800

O-O_3 -0.81958900 -5.91951000 2.97245500

H-H_ -0.01635300 -6.29381800 3.41960800

O-O_3 -5.90343800 -4.11476600 2.07851700

H-H_ -5.78592100 -5.10004500 2.10367100

O-O_3 -4.05647500 0.29345800 3.59757200

H-H_ -3.12351400 0.09281100 3.32312300

C-C_3 5.07176200 0.65318100 2.64227700

H-H_ 4.42328300 1.04223800 3.45915600

H-H_ 4.83562900 -0.42122400 2.50245500

C-C_3 4.77307400 1.43656700 1.35688800

H-H_ 3.68050900 1.31683200 1.17343100

C-C_3 5.55648200 0.85123700 0.14468100

H-H_ 6.62370700 0.75267100 0.45494900

C-C_3 5.49069800 1.79254700 -1.07765100

H-H_ 4.47555000 1.70134500 -1.53536500

C-C_3 5.71887100 3.25423700 -0.66947900

H-H_ 6.77237400 3.36150200 -0.31674500

C-C_3 4.77053500 3.61849900 0.48425300

H-H_ 4.99068800 4.66492200 0.79428300

C-C_3 4.69972600 -3.67505100 2.23005600

H-H_ 5.08104500 -3.01586700 3.04189900

H-H_ 3.69470700 -4.03607100 2.53704800

C-C_3 4.60123100 -2.85818400 0.93603500

H-H_ 3.87301600 -2.04224400 1.14499000

C-C_3 4.05352600 -3.70261300 -0.25177300

H-H_ 4.69818900 -4.59977500 -0.40910100

C-C_3 4.10504800 -2.87358800 -1.54784700

H-H_ 3.32528700 -2.07622200 -1.48089700

C-C_3 5.48622700 -2.21742900 -1.71784200

H-H_ 6.23977400 -3.02356400 -1.86278300

C-C_3 5.86703700 -1.43554400 -0.44791200

H-H_ 6.90785500 -1.07640700 -0.61929300

C-C_3 -0.47473000 -4.63294500 2.53132100

H-H_ 0.09931400 -4.09608600 3.32119000

H-H_ -1.40244400 -4.05454900 2.36458400

C-C_3 0.35567900 -4.68652500 1.23836200

H-H_ 0.54727800 -3.62588400 0.95581400

C-C_3 -0.42146800 -5.38418500 0.08739800

H-H_ -0.74810200 -6.38089200 0.46853900

C-C_3 0.49684600 -5.60651900 -1.13247400

H-H_ 0.67107900 -4.61813100 -1.62329300

C-C_3 1.84992100 -6.19710400 -0.70709100

H-H_ 1.67862900 -7.23167200 -0.32526600

C-C_3 2.45874600 -5.34755500 0.42096400

H-H_ 3.38744500 -5.85823800 0.76273000

C-C_3 -4.62165900 -3.55220700 2.17108800

H-H_ -4.03769800 -4.06307300 2.97083000

H-H_ -4.70707500 -2.49550200 2.48501000

C-C_3 -3.86463400 -3.65729800 0.83822000

H-H_ -2.92698900 -3.07306900 0.97213000

C-C_3 -4.64615100 -3.03961100 -0.36218700

H-H_ -5.63196100 -3.54757800 -0.48034400

C-C_3 -3.84934400 -3.28674200 -1.65804100

H-H_ -2.93180300 -2.64968900 -1.62661600

C-C_3 -3.43446100 -4.76308100 -1.77337600

H-H_ -4.35821500 -5.37277100 -1.89058600

C-C_3 -2.72232400 -5.23142100 -0.49209600

H-H_ -2.58536800 -6.32995300 -0.62174800

C-C_3 -4.53981300 1.25947600 2.70301400

H-H_ -3.79690200 2.07675800 2.60174300

H-H_ -5.46986500 1.69546300 3.12641500

C-C_3 -4.84065000 0.62444400 1.33764700

H-H_ -3.86679900 0.26270000 0.93468800

C-C_3 -5.44219400 1.67108500 0.35991100

H-H_ -6.33033900 2.12283600 0.86059200

C-C_3 -5.92593200 0.98499400 -0.93308500

H-H_ -5.02521100 0.69472100 -1.52653300

C-C_3 -6.75097900 -0.27795600 -0.61239300

H-H_ -7.68932200 0.04186900 -0.10654300

C-C_3 -5.98102600 -1.18628400 0.36064000

H-H_ -6.66573500 -2.02079700 0.63108300

C-C_3 -1.85105700 4.64373800 2.48768000

H-H_ -2.19332300 3.97749100 3.31087300

H-H_ -0.75650000 4.50565900 2.37301900

C-C_3 -2.57594900 4.24784300 1.19405900

H-H_ -2.32525300 3.17723900 1.01396000

C-C_3 -2.07626600 5.08556500 -0.01412400

H-H_ -2.18221300 6.16202300 0.26160700

C-C_3 -2.94134800 4.80250100 -1.25891800

H-H_ -2.68826900 3.78113400 -1.63460700

C-C_3 -4.43880100 4.84835900 -0.91347600

H-H_ -4.71978600 5.89542100 -0.64461100

C-C_3 -4.73116500 3.95463100 0.30013000

H-H_ -5.80470400 4.12425300 0.54070500

C-C_3 2.50515900 5.47077700 2.13549800

H-H_ 1.76414300 5.73980800 2.92312500

H-H_ 3.09136800 4.61466100 2.52928500

C-C_3 1.76511400 5.05838000 0.85396900

H-H_ 1.14595900 4.17584000 1.12976000

C-C_3 2.74356200 4.62641800 -0.27386000

H-H_ 3.43463600 5.46834200 -0.51721800

C-C_3 1.93883600 4.30276200 -1.54463200

H-H_ 1.35472300 3.36877900 -1.35758200

C-C_3 0.96949700 5.45130200 -1.87149500

H-H_ 1.57202000 6.35160600 -2.12646100

C-C_3 0.10349400 5.79511300 -0.64395100

H-H_ -0.46861900 6.70697900 -0.93313100

ϒ-cyclodextrin

Sum of electronic and thermal Free Energies= 1.816360 (uff)

O-O_3 0 -0.12894300 6.12661300 -2.83508300 L

H-H_ 0 -0.54335500 6.96718400 -3.16262700 L

O-O_3 0 2.58030000 5.30887700 -2.69859600 L

H-H_ 0 2.93519700 4.38272300 -2.74171400 L

O-O_3 0 0.74224700 6.99417300 0.60777400 L

O-O_3 0 3.15625200 7.46290700 1.99493500 L

H-H_ 0 3.63251300 7.61731000 2.85169600 L

O-O_3 0 3.39897500 4.52592500 -0.05169900 L

O-O_3 0 5.38526000 5.33567200 -1.91877900 L

H-H_ 0 6.20963400 5.44023500 -2.46170100 L

O-O_3 0 6.71289000 2.77832500 -2.10734800 L

H-H_ 0 6.35156300 2.99241000 -3.00695800 L

O-O_3 0 5.09922300 4.04217400 1.46644000 L

O-O_3 0 6.55797100 2.04910900 2.94259500 L

H-H_ 0 6.63093800 2.95096700 3.35068300 L

O-O_3 0 5.56980300 0.82993500 -0.24722700 L

O-O_3 0 6.02828400 -0.00354200 -2.87039100 L

H-H_ 0 6.25359400 -0.48340300 -3.70967600 L

O-O_3 0 5.19119000 -2.74446000 -2.57368500 L

H-H_ 0 4.57520600 -2.31425900 -3.22257500 L

O-O_3 0 7.08360700 -0.77454800 0.53281100 L

O-O_3 0 6.88012200 -1.48836400 3.17289300 L

H-H_ 0 6.04696800 -1.13239800 3.57856600 L

O-O_3 0 4.54959700 -3.41402100 0.13041900 L

O-O_3 0 5.45402100 -5.42365300 -1.70008100 L

H-H_ 0 5.58344300 -6.25669000 -2.22417500 L

O-O_3 0 2.90385800 -6.73148700 -1.99022600 L

H-H_ 0 3.16265200 -6.37593700 -2.88031200 L

O-O_3 0 4.02757800 -5.11603800 1.62830900 L

O-O_3 0 2.00207800 -6.59185200 3.02670100 L

H-H_ 0 2.88514200 -6.65060600 3.47615200 L

O-O_3 0 0.88144700 -5.55953200 -0.21628600 L

O-O_3 0 0.10736800 -5.93911200 -2.88258300 L

H-H_ 0 0.49741100 -6.75295300 -3.29608700 L

O-O_3 0 -2.61935200 -5.11583700 -2.64905600 L

H-H_ 0 -2.13739800 -4.53367200 -3.29281900 L

O-O_3 0 -0.75353000 -7.08075700 0.48164100 L

O-O_3 0 -1.55245300 -6.92586600 3.09982800 L

H-H_ 0 -1.21299000 -6.09906600 3.53174800 L

O-O_3 0 -3.40394400 -4.56089200 0.03796200 L

O-O_3 0 -5.33532500 -5.42734000 -1.88588500 L

H-H_ 0 -6.12872400 -5.48993600 -2.47916700 L

O-O_3 0 -6.65549400 -2.88348900 -2.15957000 L

H-H_ 0 -7.54958200 -3.11513400 -1.79491000 L

O-O_3 0 -5.15778500 -4.11506800 1.50227100 L

O-O_3 0 -6.70964300 -2.19447800 2.94083400 L

H-H_ 0 -6.74328700 -3.09252700 3.36228800 L

O-O_3 0 -5.61908900 -0.91897600 -0.25233200 L

O-O_3 0 -6.23802700 0.00518800 -2.88779000 L

H-H_ 0 -5.35000400 -0.41565700 -2.74769700 L

O-O_3 0 -5.29002800 2.69630200 -2.56552300 L

H-H_ 0 -6.16896300 3.13130800 -2.72055600 L

O-O_3 0 -7.07513200 0.68844300 0.61556000 L

O-O_3 0 -7.52830900 3.00903600 2.15081500 L

H-H_ 0 -7.68172300 3.43561400 3.03352000 L

O-O_3 0 -4.56262000 3.34658800 0.13541200 L

O-O_3 0 -5.50328900 5.43930900 -1.56583500 L

H-H_ 0 -5.58961700 6.27976600 -2.08687100 L

O-O_3 0 -2.95866400 6.73565000 -1.89919000 L

H-H_ 0 -3.15747800 7.60091500 -1.45431700 L

O-O_3 0 -3.98458000 4.96742900 1.70442800 L

O-O_3 0 -2.00137800 6.31674400 3.18543300 L

H-H_ 0 -1.26770900 6.90266900 2.86308800 L

O-O_3 0 -0.89257500 5.53031200 -0.20105500 L

C-C_3 0 -0.11051800 6.68642800 -0.48297600 L

H-H_ 0 -0.73584600 7.58798200 -0.67856400 L

C-C_3 0 0.71352800 6.47951000 -1.76859500 L

H-H_ 0 1.24876800 7.43016800 -2.00764500 L

C-C_3 0 1.75904000 5.37999600 -1.56155800 L

H-H_ 0 1.22266800 4.40961000 -1.42014200 L

C-C_3 0 2.60826600 5.67616100 -0.31135300 L

H-H_ 0 3.20758100 6.59246300 -0.52799600 L

C-C_3 0 1.65336300 5.93806700 0.88569700 L

H-H_ 0 1.10499500 4.99453100 1.09881100 L

C-C_3 0 2.40690600 6.29220200 2.17275800 L

H-H_ 0 1.66612200 6.44904700 2.98872600 L

H-H_ 0 3.06738500 5.45208200 2.47762100 L

C-C_3 0 4.73795600 4.82029500 0.33844500 L

H-H_ 0 4.87601800 5.88242100 0.64293900 L

C-C_3 0 5.72213700 4.54512300 -0.80869100 L

H-H_ 0 6.74554800 4.81244300 -0.45046700 L

C-C_3 0 5.69472500 3.05136400 -1.17917600 L

H-H_ 0 4.69951000 2.80234500 -1.62109500 L

C-C_3 0 5.89774300 2.17435600 0.07566700 L

H-H_ 0 6.94672700 2.29830000 0.43735200 L

C-C_3 0 4.95679500 2.65116300 1.21508400 L

H-H_ 0 3.90391400 2.41216900 0.93930600 L

C-C_3 0 5.22581000 1.89969300 2.52838400 L

H-H_ 0 4.53300700 2.26859000 3.31917600 L

H-H_ 0 5.01123500 0.82282600 2.39573400 L

C-C_3 0 6.71071100 0.03985800 -0.56726400 L

H-H_ 0 7.59877800 0.65718100 -0.83746600 L

C-C_3 0 6.42962600 -0.82722900 -1.80577000 L

H-H_ 0 7.37269500 -1.36356100 -2.07137200 L

C-C_3 0 5.33583500 -1.85908500 -1.49309300 L

H-H_ 0 4.37193800 -1.32365800 -1.31014800 L

C-C_3 0 5.69435200 -2.65541200 -0.22704800 L

H-H_ 0 6.58950300 -3.27964400 -0.46335300 L

C-C_3 0 6.04102500 -1.66338100 0.91538800 L

H-H_ 0 5.12039900 -1.09924500 1.18156100 L

C-C_3 0 6.48800300 -2.40314800 2.18473900 L

H-H_ 0 5.66715500 -3.04629300 2.57387100 L

H-H_ 0 7.35557500 -3.05578200 1.94899200 L

C-C_3 0 4.84279000 -4.75005900 0.52858000 L

H-H_ 0 5.89581700 -4.86991200 0.87161600 L

C-C_3 0 4.61446900 -5.74298500 -0.62134700 L

H-H_ 0 4.85968000 -6.76420400 -0.24143000 L

C-C_3 0 3.13906000 -5.71061200 -1.05508100 L

H-H_ 0 2.91442100 -4.71608200 -1.51188200 L

C-C_3 0 2.20721700 -5.90608100 0.16193900 L

H-H_ 0 2.30098400 -6.95859700 0.52255600 L

C-C_3 0 2.64494900 -4.97656600 1.32819400 L

H-H_ 0 2.41214500 -3.92151900 1.05561100 L

C-C_3 0 1.84802300 -5.26070000 2.61093600 L

H-H_ 0 0.77393600 -5.06236500 2.43739700 L

H-H_ 0 2.17570300 -4.56474700 3.41701300 L

C-C_3 0 0.09535900 -6.68809500 -0.58529100 L

H-H_ 0 0.71683100 -7.57329900 -0.85454900 L

C-C_3 0 -0.73343100 -6.37473900 -1.84569900 L

H-H_ 0 -1.27474100 -7.30013500 -2.15873000 L

C-C_3 0 -1.77432200 -5.29039600 -1.54114200 L

H-H_ 0 -1.24864700 -4.33081600 -1.31409500 L

C-C_3 0 -2.61396600 -5.68615900 -0.31568200 L

H-H_ 0 -3.21547700 -6.58407200 -0.59652100 L

C-C_3 0 -1.65811100 -6.04740500 0.85296600 L

H-H_ 0 -1.10587500 -5.12948100 1.15201600 L

C-C_3 0 -2.43641100 -6.51875200 2.08998200 L

H-H_ 0 -3.09424700 -5.70661100 2.47272000 L

H-H_ 0 -3.07813300 -7.38389700 1.81819500 L

C-C_3 0 -4.74641900 -4.88728000 0.38702300 L

H-H_ 0 -4.85932800 -5.95236100 0.69321400 L

C-C_3 0 -5.71002000 -4.64168500 -0.78510800 L

H-H_ 0 -6.73304300 -4.93392200 -0.44728800 L

C-C_3 0 -5.70360600 -3.14817700 -1.15913300 L

H-H_ 0 -4.70164700 -2.89864200 -1.57503800 L

C-C_3 0 -5.94858500 -2.26041100 0.08296200 L

H-H_ 0 -7.00449300 -2.39346800 0.41919900 L

C-C_3 0 -5.03873600 -2.71966400 1.25521500 L

H-H_ 0 -3.98226400 -2.45627200 1.01869900 L

C-C_3 0 -5.37736300 -1.97651200 2.55810000 L

H-H_ 0 -4.68551500 -2.30654600 3.36672300 L

H-H_ 0 -5.21870900 -0.88945700 2.42800400 L

C-C_3 0 -6.76321700 -0.11285600 -0.51311800 L

H-H_ 0 -7.66851900 -0.71643000 -0.75544200 L

C-C_3 0 -6.53511600 0.77765200 -1.75069000 L

H-H_ 0 -7.48663500 1.31865700 -1.95196000 L

C-C_3 0 -5.43341700 1.81900200 -1.47638200 L

H-H_ 0 -4.46584000 1.28286200 -1.34997200 L

C-C_3 0 -5.72516700 2.59337700 -0.17569900 L

H-H_ 0 -6.63101500 3.22136000 -0.35039400 L

C-C_3 0 -6.00833400 1.56707300 0.95736900 L

H-H_ 0 -5.07277700 0.99419900 1.14408000 L

C-C_3 0 -6.36244800 2.24344900 2.28679600 L

H-H_ 0 -6.52693500 1.45584900 3.05600800 L

H-H_ 0 -5.51922300 2.87932600 2.63382900 L

C-C_3 0 -4.83574300 4.66111600 0.61274400 L

H-H_ 0 -5.87618200 4.76929700 0.99396900 L

C-C_3 0 -4.64281700 5.71149800 -0.49167800 L

H-H_ 0 -4.89210300 6.70832400 -0.05518700 L

C-C_3 0 -3.17582800 5.71213600 -0.96040300 L

H-H_ 0 -2.97201600 4.74281800 -1.46819500 L

C-C_3 0 -2.20698400 5.84515600 0.23894600 L

H-H_ 0 -2.29809700 6.87213600 0.66595800 L

C-C_3 0 -2.61111200 4.84431100 1.35684400 L

H-H_ 0 -2.38882600 3.80879700 1.01049200 L

C-C_3 0 -1.78184500 5.04472000 2.63412900 L

H-H_ 0 -2.09350700 4.28407400 3.38150400 L

H-H_ 0 -0.70314700 4.86921000 2.43829200 L

Octa acid cavitand

Sum of electronic and thermal Free Energies= 4.320503

O-O_R 0 1.53116300 7.52675700 -4.74042700 M

C-C_R 0 1.17081600 6.43548500 -5.53437900 M

O-O_R 0 0.82038000 6.65033900 -6.72519000 M

C-C_R 0 1.21135400 5.04267700 -5.02545100 M

H-H_ 0 0.79348200 5.56422900 -2.96480600 M

C-C_R 0 1.01053700 4.76547800 -3.66196900 M

C-C_R 0 1.40939600 3.97465600 -5.91874500 M

H-H_ 0 1.56626900 4.16662300 -6.97318700 M

C-C_R 0 1.00192700 3.44039900 -3.19665800 M

C-C_R 0 1.44070900 2.65342500 -5.46065800 M

O-O_R 0 0.69084500 3.14168700 -1.86186800 M

C-C_R 0 1.19393900 2.39254600 -4.09760800 M

O-O_R 0 1.66087800 1.61795300 -6.38978900 M

C-C_R 0 1.47818300 3.80382800 -0.90855600 M

H-H_ 0 3.36001500 3.21032500 -1.76281400 M

H-H_ 0 1.14472400 1.37449900 -3.73271100 M

C-C_R 0 2.42214300 0.54896700 -5.89716800 M

C-C_R 0 2.88043500 3.75817100 -0.96247700 M

C-C_R 0 0.85172600 4.44831100 0.15812500 M

H-H_ 0 4.14317400 1.75736100 -5.51436200 M

H-H_ 0 0.84180600 -0.87817500 -6.15034100 M

H-H_ 0 5.09763100 3.23950100 -3.46530700 M

H-H_ 0 -0.22965200 4.48519300 0.19986500 M

C-C_R 0 6.08154300 3.04778000 -3.05636000 M

C-C_R 0 1.84791800 -0.71891200 -5.79364300 M

C-C_R 0 3.72445500 0.76295500 -5.43067000 M

O-O_R 0 9.96988700 7.90499000 -0.72080600 M

O-O_R 0 5.63779400 4.68229100 -1.34532800 M

C-C_R 0 6.52632100 3.77605900 -1.95241300 M

C-C_R 0 6.85767900 2.01797000 -3.58970800 M

O-O_R 0 6.32499200 1.23642500 -4.62127800 M

H-H_ 0 10.64632400 1.78955100 -4.18687500 M

H-H_ 0 10.06746100 5.12893000 -1.44951600 M

O-O_R 0 11.17800200 0.60753200 -6.75171300 M

C-C_R 0 3.66145900 4.38666600 0.02237000 M

C-C_R 0 1.60471100 5.06240300 1.16221300 M

C-C_R 0 9.69780900 7.13061700 0.40691100 M

H-H_ 0 8.64597300 0.47807400 -4.69089600 M

C-C_R 0 8.12515600 1.72272400 -3.04568500 M

C-C_R 0 4.43941000 -0.27221900 -4.80527600 M

C-C_R 0 7.81815500 3.53479000 -1.42219100 M

H-H_ 0 7.85225100 5.39967500 -0.41620200 M

C-C_R 0 2.52932600 -1.76247000 -5.16149300 M

C-C_3 0 10.46111300 0.82206400 -3.67120700 M

C-C_R 0 8.57988400 2.46962100 -1.94250200 M

C-C_3 0 5.16887700 4.25432200 -0.05576100 M

C-C_3 0 9.86328000 4.74834100 -0.42491800 M

C-C_R 0 3.01143000 5.03976800 1.09172500 M

C-C_3 0 8.92935400 0.56461800 -3.62023500 M

H-H_ 0 12.33044800 -0.07577200 -4.32069500 M

C-C_R 0 10.87197200 -0.40604800 -5.84371300 M

C-C_3 0 8.35156200 4.41331500 -0.30065300 M

C-C_3 0 5.78750600 -0.01657500 -4.17366200 M

O-O_R 0 0.92623400 5.82707600 2.12116300 M

O-O_R 0 1.82432500 -2.95705000 -4.92731700 M

H-H_ 0 11.44321000 5.90691900 0.51573400 M

C-C_3 0 10.34261500 5.78628300 0.60604000 M

C-C_R 0 3.82354200 -1.52651800 -4.65151300 M

O-O_R 0 5.82474600 5.03763300 0.95630200 M

C-C_3 0 11.24307700 -0.29209100 -4.39050700 M

O-O_R 0 8.91331100 7.57897500 1.28392800 M

H-H_ 0 9.52036800 2.21700900 -1.48157500 M

O-O_R 0 10.29091800 -1.44413200 -6.25642300 M

O-O_R 0 6.70161000 -1.06323300 -4.53354800 M

H-H_ 0 0.76862200 7.68171000 3.89986600 M

H-H_ 0 10.48635900 3.84095300 -0.29653300 M

H-H_ 0 10.88904500 0.92559700 -2.65480600 M

H-H_ 0 5.35841800 3.15823200 0.09180400 M

H-H_ 0 3.45907800 -4.05795500 -6.70220400 M

H-H_ 0 5.59702100 -0.00637200 -3.06867900 M

H-H_ 0 3.58331600 5.54086400 1.86160300 M

C-C_R 0 1.07271400 6.73615900 4.33006400 M

H-H_ 0 4.33439100 -2.30089300 -4.09807600 M

C-C_R 0 1.25645900 5.64508800 3.47535600 M

C-C_R 0 7.95548200 3.83930900 1.04827000 M

C-C_R 0 2.58422600 -4.11844800 -4.74611800 M

C-C_R 0 6.77711100 4.30154300 1.67413300 M

C-C_R 0 8.52395100 -0.72653000 -2.92506300 M

H-H_ 0 10.13888100 5.41560100 1.63401200 M

C-C_R 0 3.37654100 -4.62697700 -5.78563300 M

C-C_R 0 7.47234900 -1.51359100 -3.45129800 M

H-H_ 0 11.07093100 -1.26288100 -3.87756800 M

O-O_R 0 1.23688400 9.06798700 6.22782600 M

O-O_R 0 4.63025700 -6.13743200 -8.07135600 M

C-C_R 0 0.98590700 7.75259800 6.62493200 M

O-O_R 0 0.57435600 7.54860800 7.79805100 M

C-C_R 0 1.21122300 6.59975500 5.71872600 M

C-C_R 0 6.42143300 3.82441100 2.92988900 M

C-C_R 0 2.47465200 -4.82492700 -3.54644300 M

C-C_R 0 1.56234600 4.38125000 4.02316300 M

C-C_R 0 8.69061000 2.79938300 1.65329900 M

C-C_R 0 4.89058000 -6.40454000 -6.72524600 M

C-C_R 0 7.09186000 -2.69295600 -2.80735800 M

C-C_R 0 4.05062200 -5.85236600 -5.63398900 M

C-C_R 0 9.13014300 -1.12181500 -1.71841900 M

H-H_ 0 5.47996300 4.13297500 3.36814000 M

H-H_ 0 9.53210500 2.36869400 1.13268300 M

H-H_ 0 6.26810900 -3.27583300 -3.19564700 M

H-H_ 0 9.87722000 -0.49039700 -1.26468000 M

H-H_ 0 1.85248700 -4.43694500 -2.75005800 M

H-H_ 0 1.62341900 3.52028300 3.37123600 M

O-O_R 0 5.85674900 -7.16128900 -6.44083300 M

C-C_R 0 1.49900700 5.33542800 6.25194800 M

C-C_R 0 1.68183100 4.22389000 5.42387300 M

C-C_R 0 7.15029200 2.79811700 3.52623700 M

C-C_R 0 3.17166300 -6.02788000 -3.36710700 M

C-C_R 0 3.93759900 -6.54722900 -4.41615000 M

C-C_R 0 8.30047500 2.27413700 2.89980200 M

C-C_R 0 7.69655300 -3.07345600 -1.61007300 M

C-C_R 0 8.75885300 -2.31901600 -1.08201000 M

H-H_ 0 4.41335300 2.76320000 5.50611100 M

H-H_ 0 11.30461600 -3.09296800 -1.84833800 M

H-H_ 0 1.57060700 5.20287400 7.32482600 M

H-H_ 0 5.85101800 0.90268800 2.91484800 M

H-H_ 0 11.26418000 -1.56208700 0.13948600 M

H-H_ 0 5.19006900 -5.12175900 -1.90949200 M

O-O_R 0 6.53393900 2.07664500 4.54760200 M

C-C_R 0 3.82827200 1.93706000 5.12389400 M

O-O_R 0 1.84543200 2.97665500 6.05671800 M

H-H_ 0 5.95104200 -2.69083200 0.07018200 M

H-H_ 0 4.45039500 -7.49065900 -4.27364100 M

O-O_R 0 7.16464600 -4.14757500 -0.88729000 M

O-O_R 0 3.07234100 -6.74306400 -2.16484400 M

C-C_3 0 11.65902000 -3.42739400 -0.85062900 M

C-C_3 0 5.88356000 0.90380000 4.03887500 M

C-C_3 0 9.00755700 1.07971000 3.53227500 M

H-H_ 0 10.95382600 1.26103900 2.49641600 M

C-C_3 0 9.40421800 -2.76394700 0.21562300 M

C-C_R 0 4.61824900 -5.23068000 -0.99830900 M

H-H_ 0 10.81690600 2.21263000 3.98481500 M

C-C_3 0 10.95132500 -2.61889100 0.25096000 M

C-C_R 0 9.09360900 -0.83304800 1.84196100 M

O-O_R 0 10.71661400 -5.65180400 -1.60442800 M

H-H_ 0 9.89677000 -0.31608400 1.34423700 M

C-C_3 0 10.55385600 1.23282200 3.52937800 M

H-H_ 0 12.74893900 -3.21512400 -0.80992800 M

C-C_3 0 6.30273600 -3.74683000 0.19226300 M

C-C_R 0 2.44345400 1.95843700 5.30943200 M

C-C_R 0 4.43414500 0.87112600 4.43180000 M

C-C_R 0 8.47767900 -0.23423800 2.95410300 M

C-C_R 0 3.46544500 -6.03627200 -1.01434900 M

C-C_R 0 8.68631200 -2.09565600 1.37213300 M

C-C_R 0 5.04769200 -4.60228500 0.18636700 M

H-H_ 0 8.74966500 1.08023400 4.61323100 M

C-C_R 0 11.45767000 -4.90966400 -0.68395300 M

H-H_ 0 9.24352400 -3.85697200 0.33281800 M

C-C_R 0 7.35519200 -0.88045300 3.53690500 M

C-C_R 0 7.55264500 -2.71398400 1.95219900 M

O-O_R 0 6.57390200 -0.25517200 4.52356000 M

H-H_ 0 11.32415700 -2.95582700 1.24358000 M

C-C_R 0 6.92521200 -2.11731100 3.04718900 M

C-C_R 0 2.74478900 -6.22065000 0.16617500 M

C-C_R 0 3.63458400 -0.17399200 3.94640200 M

C-C_R 0 1.66190500 0.91046800 4.82497800 M

O-O_R 0 7.01385200 -3.90744300 1.43283900 M

C-C_R 0 4.26810100 -4.74956400 1.34937700 M

C-C_3 0 11.28615000 0.11383600 4.29244300 M

C-C_R 0 2.24811100 -0.15826000 4.13896400 M

C-C_R 0 3.10688700 -5.55346200 1.33954400 M

H-H_ 0 1.87859700 -6.87080500 0.16664600 M

H-H_ 0 4.07988100 -0.97339300 3.36673600 M

H-H_ 0 6.05360100 -2.58874100 3.48313700 M

H-H_ 0 0.60124800 0.93823800 4.99317700 M

O-O_R 0 11.96216100 -5.49550900 0.31000200 M

H-H_ 0 12.38210300 0.25524100 4.17883100 M

H-H_ 0 1.88025600 -3.19645200 1.97614000 M

O-O_R 0 11.36032900 1.15243200 6.59528100 M

H-H_ 0 4.58045300 -4.26946000 2.26738700 M

H-H_ 0 11.03926600 -0.87342400 3.84684700 M

C-C_R 0 10.95560100 0.11157800 5.75966300 M

C-C_R 0 1.93058500 -3.45087500 3.02715400 M

O-O_R 0 2.35763900 -5.81676900 2.50068700 M

O-O_R 0 1.44049500 -1.14555100 3.55889300 M

C-C_R 0 2.18935200 -4.77463700 3.42761900 M

C-C_R 0 1.73245700 -2.45017800 3.98237300 M

O-O_R 0 10.32047900 -0.85809100 6.25147600 M

C-C_R 0 2.15328200 -5.10017800 4.78773800 M

C-C_R 0 1.73220300 -2.78391800 5.34710700 M

C-C_R 0 1.93870000 -4.10973700 5.76077000 M

H-H_ 0 1.54357600 -2.01020900 6.08123300 M

H-H_ 0 2.26519200 -6.13831800 5.07177800 M

C-C_R 0 1.86996400 -4.45454600 7.20257000 M

O-O_R 0 1.18637200 -3.73596900 7.97946100 M

O-O_R 0 2.55024600 -5.55031200 7.73858000 M

H-H_ 0 1.96074400 7.26557700 -3.86234100 M

H-H_ 0 10.64734300 7.47119100 -1.33639100 M

H-H_ 0 11.68348900 1.37392800 -6.32369800 M

H-H_ 0 1.71857800 9.13857200 5.34075600 M

H-H_ 0 3.74463800 -5.67477900 -8.22991600 M

H-H_ 0 10.32726800 -5.08388000 -2.34734700 M

H-H_ 0 11.90301100 1.85482500 6.10716900 M

H-H_ 0 3.20794000 -5.97360700 7.09702100 M

O-O_R 0 -1.52600100 -7.52323600 -4.74529300 M

C-C_R 0 -1.16681800 -6.43096100 -5.53835500 M

O-O_R 0 -0.81549400 -6.64457500 -6.72916600 M

C-C_R 0 -1.20933100 -5.03854800 -5.02855200 M

H-H_ 0 -0.79168200 -5.56095800 -2.96805800 M

C-C_R 0 -1.00935600 -4.76200400 -3.66478100 M

C-C_R 0 -1.40820800 -3.97017000 -5.92121100 M

H-H_ 0 -1.56445700 -4.16164100 -6.97584600 M

C-C_R 0 -1.00216700 -3.43721000 -3.19867900 M

C-C_R 0 -1.44099500 -2.64925000 -5.46234800 M

O-O_R 0 -0.69152100 -3.13901500 -1.86366000 M

C-C_R 0 -1.19487100 -2.38898800 -4.09902900 M

O-O_R 0 -1.66173700 -1.61344100 -6.39098100 M

C-C_R 0 -1.47861600 -3.80220100 -0.91091700 M

H-H_ 0 -3.36059500 -3.20798000 -1.76428800 M

H-H_ 0 -1.14663200 -1.37113700 -3.73346700 M

C-C_R 0 -2.42299000 -0.54475600 -5.89770000 M

C-C_R 0 -2.88087300 -3.75656600 -0.96453700 M

C-C_R 0 -0.85193900 -4.44770100 0.15502700 M

H-H_ 0 -4.14398800 -1.75341100 -5.51561800 M

H-H_ 0 -0.84262500 0.88254100 -6.14995600 M

H-H_ 0 -5.09805800 -3.23677900 -3.46695700 M

H-H_ 0 0.22945700 -4.48460200 0.19650800 M

C-C_R 0 -6.08196700 -3.04537800 -3.05785800 M

C-C_R 0 -1.84870400 0.72302400 -5.79327300 M

C-C_R 0 -3.72525400 -0.75907300 -5.43123700 M

O-O_R 0 -9.96953600 -7.90420000 -0.72699300 M

O-O_R 0 -5.63807900 -4.68093000 -1.34785000 M

C-C_R 0 -6.52665000 -3.77434800 -1.95433500 M

C-C_R 0 -6.85822300 -2.01533200 -3.59057400 M

O-O_R 0 -6.32563600 -1.23315300 -4.62170500 M

H-H_ 0 -10.64691600 -1.78676600 -4.18729300 M

H-H_ 0 -10.06766000 -5.12749700 -1.45262000 M

O-O_R 0 -11.17914600 -0.60366500 -6.75139100 M

C-C_R 0 -3.66169700 -4.38601900 0.01985000 M

C-C_R 0 -1.60470700 -5.06274700 1.15867100 M

C-C_R 0 -9.69771900 -7.13110400 0.40166400 M

H-H_ 0 -8.64666300 -0.47481400 -4.69071300 M

C-C_R 0 -8.12569200 -1.72049100 -3.04631200 M

C-C_R 0 -4.44009600 0.27562900 -4.80492800 M

C-C_R 0 -7.81848300 -3.53352800 -1.42395100 M

H-H_ 0 -7.85239200 -5.39912600 -0.41930400 M

C-C_R 0 -2.53001000 1.76610000 -5.16022800 M

C-C_3 0 -10.46172500 -0.81954500 -3.67112000 M

C-C_R 0 -8.58033100 -2.46811200 -1.94358000 M

C-C_3 0 -5.16915000 -4.25378900 -0.05801000 M

C-C_3 0 -9.86348900 -4.74796500 -0.42762600 M

C-C_R 0 -3.01145000 -5.04011300 1.08848500 M

C-C_3 0 -8.92997500 -0.56205400 -3.62008900 M

H-H_ 0 -12.33114400 0.07863600 -4.31988900 M

C-C_R 0 -10.87292200 0.40951800 -5.84301300 M

C-C_3 0 -8.35180600 -4.41290500 -0.30305700 M

C-C_3 0 -5.78812200 0.01956100 -4.17334600 M

O-O_R 0 -0.92599800 -5.82830100 2.11676000 M

O-O_R 0 -1.82500400 2.96053600 -4.92527100 M

H-H_ 0 -11.44325300 -5.90772100 0.51182900 M

C-C_3 0 -10.34267100 -5.78705200 0.60224200 M

C-C_R 0 -3.82414700 1.52976800 -4.65022700 M

O-O_R 0 -5.82487900 -5.03786200 0.95356300 M

C-C_3 0 -11.24377600 0.29495200 -4.38979200 M

O-O_R 0 -8.91328300 -7.58038200 1.27826500 M

H-H_ 0 -9.52085100 -2.21590700 -1.48249400 M

O-O_R 0 -10.29192200 1.44776600 -6.25538500 M

O-O_R 0 -6.70228900 1.06642600 -4.53247300 M

H-H_ 0 -0.76599100 -7.68399500 3.89401000 M

H-H_ 0 -10.48666400 -3.84077800 -0.29828700 M

H-H_ 0 -10.88960100 -0.92364700 -2.65475400 M

H-H_ 0 -5.35882100 -3.15782600 0.09033700 M

H-H_ 0 -3.46094600 4.06164200 -6.69896800 M

H-H_ 0 -5.59752800 0.00866200 -3.06838600 M

H-H_ 0 -3.58317300 -5.54197000 1.85797800 M

C-C_R 0 -1.07066400 -6.73901500 4.32506100 M

H-H_ 0 -4.33488100 2.30370500 -4.09606300 M

C-C_R 0 -1.25564700 -5.64745700 3.47124800 M

C-C_R 0 -7.95580500 -3.83984900 1.04628700 M

C-C_R 0 -2.58496600 4.12178300 -4.74337600 M

C-C_R 0 -6.77742300 -4.30244300 1.67187600 M

C-C_R 0 -8.52457200 0.72867200 -2.92411700 M

H-H_ 0 -10.13893800 -5.41742500 1.63059500 M

C-C_R 0 -3.37809800 4.63038000 -5.78224300 M

C-C_R 0 -7.47295100 1.51605100 -3.44986000 M

H-H_ 0 -11.07151300 1.26551600 -3.87645700 M

O-O_R 0 -1.23191300 -9.07246300 6.22107300 M

O-O_R 0 -4.63414500 6.14086100 -8.06661800 M

C-C_R 0 -0.98208800 -7.75713500 6.61913500 M

O-O_R 0 -0.57045100 -7.55367400 7.79230900 M

C-C_R 0 -1.20870900 -6.60379200 5.71387700 M

C-C_R 0 -6.42196800 -3.82631800 2.92808000 M

C-C_R 0 -2.47502700 4.82783800 -3.54349200 M

C-C_R 0 -1.56232100 -4.38426100 4.02014700 M

C-C_R 0 -8.69102900 -2.80040300 1.65202200 M

C-C_R 0 -4.89363900 6.40750800 -6.72024500 M

C-C_R 0 -7.09234500 2.69492600 -2.80510100 M

C-C_R 0 -4.05269200 5.85535900 -5.62972100 M

C-C_R 0 -9.13069900 1.12317100 -1.71717100 M

H-H_ 0 -5.48052800 -4.13516000 3.36620000 M

H-H_ 0 -9.53244600 -2.36932500 1.13160400 M

H-H_ 0 -6.26856800 3.27802000 -3.19301400 M

H-H_ 0 -9.87778300 0.49148600 -1.26382500 M

H-H_ 0 -1.85230900 4.43977600 -2.74757800 M

H-H_ 0 -1.62444700 -3.52287200 3.36888500 M

O-O_R 0 -5.85996200 7.16370700 -6.43495800 M

C-C_R 0 -1.49735100 -5.34011200 6.24817300 M

C-C_R 0 -1.68141200 -4.22808200 5.42103700 M

C-C_R 0 -7.15096500 -2.80053500 3.52516200 M

C-C_R 0 -3.17258500 6.03032200 -3.36323300 M

C-C_R 0 -3.93937000 6.54975700 -4.41163000 M

C-C_R 0 -8.30105700 -2.27606400 2.89896000 M

C-C_R 0 -7.69693800 3.07460000 -1.60750800 M

C-C_R 0 -8.75925700 2.31988200 -1.07992500 M

H-H_ 0 -4.41414700 -2.76713800 5.50514700 M

H-H_ 0 -11.30522600 3.09495100 -1.84512300 M

H-H_ 0 -1.56870900 -5.20846100 7.32117500 M

H-H_ 0 -5.85161100 -0.90482300 2.91506600 M

H-H_ 0 -11.26445900 1.56231900 0.14126200 M

H-H_ 0 -5.19094100 5.12347700 -1.90578800 M

O-O_R 0 -6.53469100 -2.07974600 4.54705500 M

C-C_R 0 -3.82895200 -1.94091300 5.12328400 M

O-O_R 0 -1.84620900 -2.98156500 6.05501600 M

H-H_ 0 -5.95120200 2.69062700 0.07221800 M

H-H_ 0 -4.45265000 7.49280700 -4.26839200 M

O-O_R 0 -7.16492400 4.14814200 -0.88396300 M

O-O_R 0 -3.07326300 6.74479700 -2.16055100 M

C-C_3 0 -11.65930100 3.42859100 -0.84703200 M

C-C_3 0 -5.88416500 -0.90665400 4.03909500 M

C-C_3 0 -9.00813000 -1.08197400 3.53211800 M

H-H_ 0 -10.95421300 -1.26264000 2.49576200 M

C-C_3 0 -9.40436300 2.76391700 0.21813700 M

C-C_R 0 -4.61876100 5.23149900 -0.99472500 M

H-H_ 0 -10.81768200 -2.21497500 3.98371000 M

C-C_3 0 -10.95147100 2.61898500 0.25365200 M

C-C_R 0 -9.09377700 0.83185300 1.84302400 M

O-O_R 0 -10.71684600 5.65351700 -1.59927000 M

H-H_ 0 -9.89695500 0.31529500 1.34490700 M

C-C_3 0 -10.55445700 -1.23495700 3.52882100 M

H-H_ 0 -12.74925400 3.21651300 -0.80620200 M

C-C_3 0 -6.30280800 3.74656500 0.19511500 M

C-C_R 0 -2.44409600 -1.96279800 5.30836900 M

C-C_R 0 -4.43473200 -0.87441800 4.43197000 M

C-C_R 0 -8.47803100 0.23229700 2.95485500 M

C-C_R 0 -3.46592900 6.03704200 -1.01049800 M

C-C_R 0 -8.68635000 2.09472500 1.37404400 M

C-C_R 0 -5.04771800 4.60197400 0.18952800 M

H-H_ 0 -8.75043000 -1.08320500 4.61312300 M

C-C_R 0 -11.45760900 4.91068200 -0.67911700 M

H-H_ 0 -9.24353500 3.85683300 0.33614200 M

C-C_R 0 -7.35554300 0.87806000 3.53817500 M

C-C_R 0 -7.55260300 2.71255500 1.95451100 M

O-O_R 0 -6.57438400 0.25208600 4.52449000 M

H-H_ 0 -11.32407000 2.95504100 1.24666000 M

C-C_R 0 -6.92534300 2.11514300 3.04920100 M

C-C_R 0 -2.74474000 6.22022800 0.16987600 M

C-C_R 0 -3.63503600 0.17082500 3.94705200 M

C-C_R 0 -1.66238300 -0.91478100 4.82426300 M

O-O_R 0 -7.01364200 3.90631500 1.43597200 M

C-C_R 0 -4.26762100 4.74808600 1.35234500 M

C-C_3 0 -11.28687300 -0.11629600 4.29224000 M

C-C_R 0 -2.24851200 0.15458400 4.13916100 M

C-C_R 0 -3.10632900 5.55190900 1.34276100 M

H-H_ 0 -1.87850000 6.87033200 0.17057400 M

H-H_ 0 -4.08028700 0.97068900 3.36798500 M

H-H_ 0 -6.05369700 2.58620200 3.48549400 M

H-H_ 0 -0.60168500 -0.94301000 4.99206800 M

O-O_R 0 -11.96147000 5.49571300 0.31563900 M

H-H_ 0 -12.38280300 -0.25750200 4.17816200 M

H-H_ 0 -1.88057800 3.19377600 1.97774800 M

O-O_R 0 -11.36209800 -1.15613300 6.59447600 M

H-H_ 0 -4.57963400 4.26712900 2.27002100 M

H-H_ 0 -11.03972900 0.87118600 3.84728800 M

C-C_R 0 -10.95685200 -0.11490400 5.75957400 M

C-C_R 0 -1.93037600 3.44771700 3.02890600 M

O-O_R 0 -2.35648600 5.81409600 2.50377300 M

O-O_R 0 -1.44079400 1.14196800 3.55937400 M

C-C_R 0 -2.18834200 4.77141200 3.43011200 M

C-C_R 0 -1.73227800 2.44647400 3.98358000 M

O-O_R 0 -10.32183600 0.85443600 6.25217400 M

C-C_R 0 -2.15140000 5.09632100 4.79035000 M

C-C_R 0 -1.73135100 2.77957700 5.34846400 M

C-C_R 0 -1.93690500 4.10531900 5.76285400 M

H-H_ 0 -1.54277400 2.00542700 6.08214200 M

H-H_ 0 -2.26249000 6.13441100 5.07485300 M

C-C_R 0 -1.86710300 4.44945600 7.20477900 M

O-O_R 0 -1.18401000 3.72970500 7.98105600 M

O-O_R 0 -2.54561100 5.54592000 7.74159400 M

H-H_ 0 -1.95629700 -7.26320700 -3.86720600 M

H-H_ 0 -10.64693300 -7.46976400 -1.34219200 M

H-H_ 0 -11.68457800 -1.37023100 -6.32361700 M

H-H_ 0 -1.71377600 -9.14283900 5.33408300 M

H-H_ 0 -3.74843600 5.67865100 -8.22594200 M

H-H_ 0 -10.32801400 5.08620000 -2.34292200 M

H-H_ 0 -11.90470400 -1.85817200 6.10576800 M

H-H_ 0 -3.20314400 5.97034400 7.10061800 M

3-hydroxyflavone with β-cyclodextrin 1:1 inclusion complex

ONIOM: gridpoint 1 method: low system: model energy: 0.132613824882
ONIOM: gridpoint 2 method: high system: model energy: -803.487455584261
ONIOM: gridpoint 3 method: low system: real energy: 0.405373600857
ONIOM: extrapolated energy = -803.214695808286

Sum of electronic and thermal Free Energies= -801.738360

O-O_3 0 -2.92497000 3.66417500 2.43087900 L

H-H_ 0 -2.42555500 3.13217000 3.10333800 L

O-O_3 0 -5.64773400 3.91579800 1.65178900 L

H-H_ 0 -6.00042000 4.82151200 1.44944300 L

O-O_3 0 -6.73021700 1.26077800 1.84068500 L

H-H_ 0 -6.40891100 1.56923500 2.72812100 L

O-O_3 0 -6.09034200 -1.78495300 2.45122600 L

H-H_ 0 -5.43681300 -1.04249400 2.51940500 L

O-O_3 0 -4.12559200 -3.87525600 2.27468500 L

H-H_ 0 -3.95572400 -3.32430600 3.08303700 L

O-O_3 0 -2.54308100 -6.12584300 1.43537300 L

H-H_ 0 -3.21958700 -6.77165100 1.10223100 L

O-O_3 0 0.28673700 -6.10292800 1.85323600 L

H-H_ 0 -0.18434900 -6.00910200 2.72209300 L

O-O_3 0 2.59195000 -4.36004700 2.96893600 L

H-H_ 0 1.85219500 -3.71723700 2.80987400 L

O-O_3 0 4.75176000 -2.46338600 2.84634500 L

H-H_ 0 4.09818200 -2.17352500 3.53530200 L

O-O_3 0 7.43202800 -0.59455400 1.92774300 L

H-H_ 0 6.57408300 -0.74223900 2.40128000 L

O-O_3 0 6.99941200 2.24793100 1.48147000 L

H-H_ 0 6.97538200 2.02676700 2.44910900 L

O-O_3 0 5.27982100 4.87142100 1.17197800 L

H-H_ 0 5.18195300 5.71083700 1.69300700 L

O-O_3 0 2.84032900 3.57298300 1.50016500 L

H-H_ 0 3.18301500 3.82305500 2.39752100 L

O-O_3 0 -0.18879900 4.52169300 2.75387000 L

H-H_ 0 0.21649800 3.64929300 2.50855200 L

O-O_3 0 0.62914800 4.18981700 0.04766500 L

O-O_3 0 -3.63273000 3.16619300 -0.30473600 L

O-O_3 0 -5.24952800 -0.50872400 -0.00666300 L

O-O_3 0 -2.66858800 -3.82458000 -0.22553100 L

O-O_3 0 1.63776700 -4.05942100 0.29630500 L

O-O_3 0 5.14348300 -1.34563700 0.25031400 L

O-O_3 0 4.66101300 2.56981100 -0.31023200 L

O-O_3 0 -1.01132600 5.72511700 -0.60876500 L

O-O_3 0 -5.38902500 2.70408800 -1.75897800 L

O-O_3 0 -5.95580500 -2.42698500 -1.13589400 L

O-O_3 0 -1.29017600 -4.89670000 -1.76411400 L

O-O_3 0 3.74759500 -4.68447300 -0.49107600 L

O-O_3 0 6.35808600 -0.43720000 -1.52879800 L

O-O_3 0 3.69201100 3.90805100 -1.95945300 L

O-O_3 0 1.28459600 4.69263700 -3.32144400 L

H-H_ 0 0.64083800 4.54383900 -4.06183000 L

O-O_3 0 -3.44546500 6.31059300 -1.99121500 L

H-H_ 0 -2.83872100 7.09536900 -1.95599000 L

O-O_3 0 -6.89545800 0.80620900 -3.15085300 L

H-H_ 0 -7.08570900 0.30578500 -3.98644200 L

O-O_3 0 -5.25947200 -4.74290900 -2.62412100 L

H-H_ 0 -5.12488800 -5.17885300 -3.50533600 L

O-O_3 0 1.30453100 -5.03660700 -3.04744400 L

H-H_ 0 0.59663800 -5.51531900 -3.55235000 L

O-O_3 0 6.19797200 -3.93531600 -1.88285900 L

H-H_ 0 6.04482400 -4.91556300 -1.85307800 L

O-O_3 0 5.09495400 0.10744600 -3.88624100 L

H-H_ 0 4.14005900 -0.13477900 -3.76291600 L

C-C_3 0 -5.53813900 0.59592800 -2.87235200 L

H-H_ 0 -4.92293500 0.99364200 -3.71059300 L

H-H_ 0 -5.33291400 -0.48981000 -2.78748300 L

C-C_3 0 -5.13737400 1.31649800 -1.57914000 L

H-H_ 0 -4.04431600 1.14260000 -1.46604100 L

C-C_3 0 -5.87648700 0.71989300 -0.34106800 L

H-H_ 0 -6.95781100 0.63428300 -0.59823000 L

C-C_3 0 -5.75726700 1.63460300 0.89894300 L

H-H_ 0 -4.73991000 1.49759100 1.33692400 L

C-C_3 0 -5.92818500 3.11402700 0.53477400 L

H-H_ 0 -6.98325300 3.28047500 0.21121300 L

C-C_3 0 -4.98914100 3.45802500 -0.62997200 L

H-H_ 0 -5.14415800 4.52730300 -0.89797200 L

C-C_3 0 -4.49635800 -3.56795600 -2.65135200 L

H-H_ 0 -4.87325900 -2.90278600 -3.46009100 L

H-H_ 0 -3.43473500 -3.80157500 -2.88113400 L

C-C_3 0 -4.60071800 -2.82912900 -1.31164200 L

H-H_ 0 -3.94652000 -1.93615800 -1.41089000 L

C-C_3 0 -4.07793200 -3.69143100 -0.12264700 L

H-H_ 0 -4.62199100 -4.66523700 -0.09534500 L

C-C_3 0 -4.36408800 -2.98470300 1.21573500 L

H-H_ 0 -3.67324300 -2.11150600 1.29788200 L

C-C_3 0 -5.82096100 -2.49076100 1.26506500 L

H-H_ 0 -6.49348100 -3.37717200 1.24219800 L

C-C_3 0 -6.12208000 -1.63167200 0.02632300 L

H-H_ 0 -7.19714100 -1.35184500 0.10067900 L

C-C_3 0 0.72020400 -3.86310000 -2.54802900 L

H-H_ 0 0.11208200 -3.37061600 -3.34063900 L

H-H_ 0 1.52110600 -3.15072500 -2.27809800 L

C-C_3 0 -0.16473800 -4.14888100 -1.32428600 L

H-H_ 0 -0.48379300 -3.15360000 -0.93908000 L

C-C_3 0 0.60802600 -4.90026600 -0.20771800 L

H-H_ 0 0.99122100 -5.84917900 -0.65529100 L

C-C_3 0 -0.35773800 -5.24910500 0.94337000 L

H-H_ 0 -0.64850100 -4.29993800 1.45616700 L

C-C_3 0 -1.61901700 -5.93622800 0.39639400 L

H-H_ 0 -1.32479000 -6.92664000 -0.02553600 L

C-C_3 0 -2.23861900 -5.08484700 -0.72729400 L

H-H_ 0 -3.06612000 -5.68039000 -1.17528300 L

C-C_3 0 4.94129800 -3.33585900 -2.05274000 L

H-H_ 0 4.36729800 -3.86845700 -2.84554300 L

H-H_ 0 5.07376200 -2.30216100 -2.41853100 L

C-C_3 0 4.13446100 -3.34015700 -0.74644700 L

H-H_ 0 3.24255000 -2.70417700 -0.93486300 L

C-C_3 0 4.90524900 -2.72908200 0.46053000 L

H-H_ 0 5.83276900 -3.31437700 0.66094300 L

C-C_3 0 4.01304600 -2.83585900 1.71225500 L

H-H_ 0 3.14660300 -2.14350300 1.58161300 L

C-C_3 0 3.48516800 -4.26931100 1.88583300 L

H-H_ 0 4.35278100 -4.92936000 2.11010100 L

C-C_3 0 2.83302200 -4.77853600 0.58641200 L

H-H_ 0 2.64410200 -5.86243100 0.76696500 L

C-C_3 0 5.36076000 1.15951200 -2.99772600 L

H-H_ 0 4.57497500 1.93405300 -3.10188700 L

H-H_ 0 6.33269500 1.61759700 -3.27956400 L

C-C_3 0 5.43213400 0.64090500 -1.55319300 L

H-H_ 0 4.41102500 0.27365500 -1.30926100 L

C-C_3 0 5.81657400 1.78573900 -0.56277000 L

H-H_ 0 6.67662700 2.35106900 -0.99080800 L

C-C_3 0 6.27754100 1.24525100 0.81277200 L

H-H_ 0 5.36893100 0.98977600 1.40651200 L

C-C_3 0 7.14200300 -0.02166500 0.67693600 L

H-H_ 0 8.10933100 0.26365700 0.20634900 L

C-C_3 0 6.44357600 -1.02750300 -0.24560900 L

H-H_ 0 7.11270200 -1.91204600 -0.33536500 L

C-C_3 0 1.46276200 3.44655400 -2.70585900 L

H-H_ 0 1.91115300 2.73424700 -3.43432700 L

H-H_ 0 0.48466200 3.02708400 -2.39116500 L

C-C_3 0 2.39348900 3.56715700 -1.49321000 L

H-H_ 0 2.39620000 2.55908100 -1.02184800 L

C-C_3 0 1.88672200 4.60794400 -0.46142000 L

H-H_ 0 1.82210800 5.59333300 -0.98372600 L

C-C_3 0 2.90309800 4.72636300 0.69668400 L

H-H_ 0 2.63898000 5.62079300 1.30741800 L

C-C_3 0 4.32982200 4.92443500 0.13941300 L

H-H_ 0 4.38223000 5.92160500 -0.35866000 L

C-C_3 0 4.65415700 3.85484300 -0.91763900 L

H-H_ 0 5.63351700 4.13963600 -1.36234900 L

C-C_3 0 -2.64656200 5.19353600 -2.27842600 L

H-H_ 0 -1.90738800 5.44282600 -3.07415400 L

H-H_ 0 -3.28844900 4.38396800 -2.68359500 L

C-C_3 0 -1.90554000 4.70245000 -1.02575900 L

H-H_ 0 -1.34160300 3.79903900 -1.34031200 L

C-C_3 0 -2.87902800 4.29132800 0.11137600 L

H-H_ 0 -3.50418400 5.16674200 0.40876500 L

C-C_3 0 -2.06120600 3.86031700 1.34194200 L

H-H_ 0 -1.54261700 2.90197600 1.09836800 L

C-C_3 0 -1.01315800 4.93190400 1.68977300 L

H-H_ 0 -1.55208400 5.84933500 2.01590800 L

C-C_3 0 -0.17295600 5.29456600 0.45117400 L

H-H_ 0 0.44452800 6.16971700 0.76069300 L

C-C_R 0 -1.15279200 0.25177200 2.52411300 H

C-C_R 0 -2.41800700 0.19480800 3.11611800 H

C-C_R 0 -2.53227400 0.40880300 4.47774300 H

C-C_R 0 -1.39553300 0.67929000 5.26050000 H

C-C_R 0 -0.14766900 0.72817400 4.66983600 H

C-C_R 0 -0.00796500 0.51209700 3.28661500 H

H-H_ 0 -3.27983900 -0.00655200 2.49497900 H

H-H_ 0 -3.51220000 0.37019000 4.94167400 H

H-H_ 0 -1.50164000 0.84772500 6.32643900 H

H-H_ 0 0.74731100 0.93093100 5.24666900 H

C-C_R 0 1.28101100 0.54322900 2.61303400 H

C-C_R 0 1.24920200 0.31608200 1.17266100 H

C-C_R 0 0.08765200 0.09027700 0.49056300 H

C-C_R 0 -0.10105400 -0.11446900 -0.94792800 H

C-C_R 0 -1.39960900 -0.31058500 -1.45258100 H

C-C_R 0 0.97743400 -0.11132600 -1.85176700 H

C-C_R 0 -1.61460000 -0.48745800 -2.81313500 H

C-C_R 0 0.75106300 -0.28426700 -3.21287500 H

H-H_ 0 1.98379600 0.02728500 -1.48960400 H

C-C_R 0 -0.54044100 -0.47023700 -3.70178400 H

H-H_ 0 -2.62376300 -0.63737700 -3.18221500 H

H-H_ 0 1.59389400 -0.27549600 -3.89564200 H

H-H_ 0 -0.70847100 -0.60623200 -4.76506600 H

O-O_R 0 2.37321500 0.74684300 3.15793400 H

O-O_R 0 2.46231700 0.35741600 0.58040600 H

H-H_ 0 3.08389300 0.53935700 1.31354700 H

O-O_R 0 -1.09579200 0.04885400 1.18337500 H

H-H_ 0 -2.23514200 -0.32420000 -0.76697400 H

3-hydroxyflavone with β-cyclodextrin 1:1 Capped with primary side

ONIOM: gridpoint 1 method: low system: model energy: 0.120250908406
ONIOM: gridpoint 2 method: high system: model energy: -803.486297615105
ONIOM: gridpoint 3 method: low system: real energy: 0.405345548956
ONIOM: extrapolated energy = -803.201202974555
Sum of electronic and thermal Free Energies= -801.723895

O-O_3 0 0.51039200 5.22304600 -2.68636700 L

H-H_ 0 0.42249000 4.55182600 -3.41252700 L

O-O_3 0 2.62852100 6.62797600 -1.58823800 L

H-H_ 0 2.49342100 7.55701100 -1.26516100 L

O-O_3 0 4.84539700 4.87410200 -1.94833600 L

H-H_ 0 4.42653200 5.07725500 -2.82530300 L

O-O_3 0 5.20714100 2.11174100 -3.14790500 L

H-H_ 0 4.24734400 2.22574000 -2.92232300 L

O-O_3 0 5.16617700 -0.76346400 -3.08161300 L

H-H_ 0 4.47363500 -0.42797800 -3.70889000 L

O-O_3 0 5.94620700 -3.42569300 -2.38399600 L

H-H_ 0 6.90725000 -3.54688300 -2.16617400 L

O-O_3 0 3.70904400 -5.16606800 -2.74325000 L

H-H_ 0 3.93252800 -4.71332600 -3.59817400 L

O-O_3 0 0.89978900 -6.34879300 -3.07421800 L

H-H_ 0 1.25869300 -5.47826600 -3.38623500 L

O-O_3 0 -1.78760900 -5.42139200 -3.45933000 L

H-H_ 0 -1.25801100 -5.25742700 -4.28290300 L

O-O_3 0 -3.02747100 -2.81672400 -4.18954100 L

H-H_ 0 -2.15880100 -2.55120400 -3.78951500 L

O-O_3 0 -4.30086300 -0.30229900 -3.67841700 L

H-H_ 0 -3.45009000 -0.14452200 -4.16517100 L

O-O_3 0 -6.44380600 1.29200000 -2.96889700 L

H-H_ 0 -6.92707500 1.93160800 -3.55430800 L

O-O_3 0 -5.08138800 3.83338400 -2.91515500 L

H-H_ 0 -4.93677700 3.43646700 -3.81364700 L

O-O_3 0 -2.26267300 4.86348600 -3.32846100 L

H-H_ 0 -2.24286500 3.87716600 -3.21879200 L

O-O_3 0 -3.04038900 3.83376800 -0.76903000 L

O-O_3 0 1.12106400 4.78115800 0.08795200 L

O-O_3 0 4.52541900 2.40649800 -0.34785600 L

O-O_3 0 4.72068500 -1.75554100 -0.38349600 L

O-O_3 0 1.53555000 -4.54276100 -0.92520900 L

O-O_3 0 -2.61375700 -3.46290000 -1.40937000 L

O-O_3 0 -4.78225700 0.14312700 -0.85284300 L

O-O_3 0 -2.40206900 5.86213900 0.19083400 L

O-O_3 0 2.83523600 4.98161700 1.64834600 L

O-O_3 0 6.57015600 1.38432700 0.15325500 L

O-O_3 0 4.55404600 -3.62805600 0.97922400 L

O-O_3 0 -0.15479300 -5.56590000 0.33024100 L

O-O_3 0 -4.94231200 -3.52071600 -1.16202400 L

O-O_3 0 -5.73079600 1.72623000 0.55804300 L

O-O_3 0 -5.45129200 4.03812000 2.10235900 L

H-H_ 0 -5.28746100 4.48053500 2.97544700 L

O-O_3 0 -0.63894500 7.32763800 1.89792400 L

H-H_ 0 -1.53282000 7.75409800 1.83170100 L

O-O_3 0 4.97677400 3.87811400 3.04023900 L

H-H_ 0 5.33840400 3.41082500 3.83760300 L

O-O_3 0 7.98674400 -0.85837000 1.13539700 L

H-H_ 0 8.44369400 -1.30820900 1.89273800 L

O-O_3 0 2.77164400 -5.57372000 2.22788200 L

H-H_ 0 3.68451500 -5.57011100 2.61764400 L

O-O_3 0 -2.70827500 -5.41600200 1.59347100 L

H-H_ 0 -2.12961400 -6.09944600 2.02155400 L

O-O_3 0 -6.01784000 -3.32716600 1.35879600 L

H-H_ 0 -5.24539700 -3.14739500 1.95557700 L

C-C_3 0 3.94401700 3.06753500 2.54979700 L

H-H_ 0 3.15518300 2.96174400 3.32816400 L

H-H_ 0 4.33513800 2.05620400 2.32422400 L

C-C_3 0 3.32152900 3.69257300 1.29423600 L

H-H_ 0 2.47270500 3.02831500 1.01020900 L

C-C_3 0 4.34282300 3.73583900 0.11684600 L

H-H_ 0 5.28484000 4.19474000 0.50151700 L

C-C_3 0 3.80243100 4.59596800 -1.04965500 L

H-H_ 0 3.00048500 4.01735900 -1.56885300 L

C-C_3 0 3.21023800 5.91261800 -0.53090300 L

H-H_ 0 4.03679100 6.51741300 -0.08692600 L

C-C_3 0 2.17493800 5.61279400 0.56642200 L

H-H_ 0 1.79768000 6.58354900 0.95989200 L

C-C_3 0 6.71284500 -0.50842100 1.60284500 L

H-H_ 0 6.81484800 0.19796900 2.45726000 L

H-H_ 0 6.17900900 -1.40881200 1.97555500 L

C-C_3 0 5.90371600 0.17392800 0.49299800 L

H-H_ 0 4.91466900 0.40205500 0.93291200 L

C-C_3 0 5.67738300 -0.76688700 -0.73113300 L

H-H_ 0 6.65367000 -1.18901300 -1.06723100 L

C-C_3 0 5.07706000 0.01560300 -1.91662500 L

H-H_ 0 4.00293000 0.22020300 -1.68529700 L

C-C_3 0 5.80552500 1.35303200 -2.12572300 L

H-H_ 0 6.85301400 1.13827000 -2.43405100 L

C-C_3 0 5.84799700 2.13725500 -0.80515000 L

H-H_ 0 6.43155600 3.06349500 -1.01202000 L

C-C_3 0 2.44715700 -4.23761200 1.95021700 L

H-H_ 0 2.71518100 -3.59243000 2.81644100 L

H-H_ 0 1.35073100 -4.15036100 1.82258100 L

C-C_3 0 3.16425900 -3.73283300 0.68786600 L

H-H_ 0 2.73337800 -2.72679500 0.48124800 L

C-C_3 0 2.90156500 -4.65011400 -0.54024600 L

H-H_ 0 3.23553700 -5.68173400 -0.28458200 L

C-C_3 0 3.75875600 -4.18873000 -1.73642200 L

H-H_ 0 3.33924900 -3.22696000 -2.12049000 L

C-C_3 0 5.21753100 -3.96580600 -1.31323000 L

H-H_ 0 5.65382600 -4.95277800 -1.02760800 L

C-C_3 0 5.27144700 -3.03322500 -0.09023900 L

H-H_ 0 6.33339900 -2.97002400 0.23694300 L

C-C_3 0 -1.90241400 -4.29111700 1.36289900 L

H-H_ 0 -1.24932000 -4.09634300 2.24452000 L

H-H_ 0 -2.55544300 -3.40536400 1.24871400 L

C-C_3 0 -1.03562500 -4.47176100 0.10582700 L

H-H_ 0 -0.45431300 -3.52674600 -0.00277700 L

C-C_3 0 -1.91043100 -4.67252500 -1.16496200 L

H-H_ 0 -2.59564000 -5.53345900 -0.97729600 L

C-C_3 0 -1.00459600 -4.99183900 -2.37585900 L

H-H_ 0 -0.45633500 -4.05822400 -2.64948000 L

C-C_3 0 0.00933500 -6.09428700 -2.01601100 L

H-H_ 0 -0.55586700 -7.03329100 -1.82175300 L

C-C_3 0 0.77097600 -5.73085500 -0.73112300 L

H-H_ 0 1.38814600 -6.62301100 -0.48485300 L

C-C_3 0 -6.11162200 -2.23905500 0.47840900 L

H-H_ 0 -6.12316600 -1.29026600 1.05373700 L

H-H_ 0 -7.07451200 -2.31500200 -0.07036100 L

C-C_3 0 -4.94780500 -2.25100100 -0.52160700 L

H-H_ 0 -4.01803000 -2.11753100 0.06824200 L

C-C_3 0 -5.07117400 -1.07018700 -1.52962400 L

H-H_ 0 -6.08296100 -1.09345200 -1.99984200 L

C-C_3 0 -4.02267600 -1.21615800 -2.64989300 L

H-H_ 0 -3.01816000 -0.99482200 -2.21305200 L

C-C_3 0 -4.02494500 -2.64586400 -3.21270700 L

H-H_ 0 -5.00845600 -2.83016300 -3.69977800 L

C-C_3 0 -3.86236800 -3.65904300 -2.06846400 L

H-H_ 0 -3.95582200 -4.66747800 -2.53086300 L

C-C_3 0 -4.36527300 3.17744400 1.89280500 L

H-H_ 0 -4.33143000 2.42319800 2.70972500 L

H-H_ 0 -3.41548900 3.75308100 1.91986000 L

C-C_3 0 -4.50651600 2.45380500 0.54619700 L

H-H_ 0 -3.64628000 1.75157400 0.48877500 L

C-C_3 0 -4.40401500 3.45822400 -0.63961000 L

H-H_ 0 -5.08772400 4.31061100 -0.41307700 L

C-C_3 0 -4.84751600 2.81741100 -1.97353000 L

H-H_ 0 -4.02575000 2.15067400 -2.33101500 L

C-C_3 0 -6.12095700 1.97856200 -1.78829900 L

H-H_ 0 -6.96466800 2.65362900 -1.50580700 L

C-C_3 0 -5.91074500 0.98463400 -0.63621600 L

H-H_ 0 -6.84681900 0.39533700 -0.50792300 L

C-C_3 0 -0.85704200 5.94743800 2.02519400 L

H-H_ 0 -1.69265100 5.75349500 2.73641100 L

H-H_ 0 0.04891800 5.47732100 2.46151700 L

C-C_3 0 -1.17949200 5.30726500 0.66639900 L

H-H_ 0 -1.29979800 4.21764000 0.86479800 L

C-C_3 0 -0.02248500 5.49619600 -0.35548100 L

H-H_ 0 0.16072600 6.58600700 -0.51115700 L

C-C_3 0 -0.44347800 4.89755300 -1.70944700 L

H-H_ 0 -0.50128400 3.78748800 -1.59560700 L

C-C_3 0 -1.82162500 5.44188400 -2.12458100 L

H-H_ 0 -1.72694900 6.53780400 -2.29375200 L

C-C_3 0 -2.84722800 5.22397500 -0.99606100 L

H-H_ 0 -3.77484200 5.74249000 -1.33167000 L

C-C_R 0 0.58985600 -1.21739800 3.74472600 H

C-C_R 0 1.48736000 -1.46405500 4.78886700 H

C-C_R 0 2.82920700 -1.19049500 4.59277300 H

C-C_R 0 3.28895400 -0.68207300 3.36551700 H

C-C_R 0 2.39944500 -0.46559400 2.33167500 H

C-C_R 0 1.03167700 -0.73345000 2.50758200 H

H-H_ 0 1.11252200 -1.84387500 5.73220800 H

H-H_ 0 3.53236900 -1.36465000 5.40030200 H

H-H_ 0 4.34017100 -0.45580200 3.24512700 H

H-H_ 0 2.70933200 -0.08177800 1.36783300 H

C-C_R 0 0.05350100 -0.49370800 1.45642100 H

C-C_R 0 -1.33730300 -0.70547200 1.83543100 H

C-C_R 0 -1.69482300 -1.14371400 3.07738800 H

C-C_R 0 -3.04490800 -1.33997800 3.61591300 H

C-C_R 0 -3.24808300 -2.20551600 4.70427200 H

C-C_R 0 -4.13996500 -0.62420800 3.10743100 H

C-C_R 0 -4.51061600 -2.34580500 5.26820900 H

C-C_R 0 -5.39324400 -0.74500100 3.69872000 H

H-H_ 0 -4.00334500 0.02322700 2.25450000 H

C-C_R 0 -5.58679500 -1.60814700 4.77538900 H

H-H_ 0 -4.65319700 -3.02692400 6.10088100 H

H-H_ 0 -6.22390600 -0.16523800 3.30957000 H

H-H_ 0 -6.56882200 -1.70726300 5.22624300 H

O-O_R 0 0.31207500 -0.11330900 0.30827600 H

O-O_R 0 -2.23760200 -0.42615000 0.86722600 H

H-H_ 0 -1.70466900 -0.16385300 0.09062600 H

O-O_R 0 -0.72437400 -1.44604800 3.99773900 H

H-H_ 0 -2.41292500 -2.76600100 5.10539500 H

3-hydroxyflavone with β-cyclodextrin 1:1 Capped with secondary side

ONIOM: gridpoint 1 method: low system: model energy: 0.131592901576
ONIOM: gridpoint 2 method: high system: model energy: -803.486331060772
ONIOM: gridpoint 3 method: low system: real energy: 0.415611047029
ONIOM: extrapolated energy = -803.202312915319
Sum of electronic and thermal Free Energies= -801.726953

O-O_3 0 -6.64943500 -2.10866900 -0.56044000 L

H-H_ 0 -7.04555700 -1.84480300 0.31099900 L

O-O_3 0 -4.33838800 -3.62624000 1.17397300 L

H-H_ 0 -5.26235600 -3.98521500 1.12170200 L

O-O_3 0 -1.78077000 -4.85460200 1.66778600 L

H-H_ 0 -2.06306900 -4.29244900 2.43519500 L

O-O_3 0 1.21908900 -4.98121000 2.11571200 L

H-H_ 0 0.73898100 -4.11289300 2.08367300 L

O-O_3 0 3.81329300 -3.78585900 1.71375000 L

H-H_ 0 3.34564600 -3.41862300 2.50809300 L

O-O_3 0 6.45033200 -3.57806300 0.62335000 L

H-H_ 0 6.86589300 -4.37765700 0.20666400 L

O-O_3 0 7.29101900 -0.90160300 1.24515400 L

H-H_ 0 7.11499100 -1.41600400 2.07598700 L

O-O_3 0 6.52299800 1.91535600 2.48024300 L

H-H_ 0 5.94480800 1.10892500 2.50592100 L

O-O_3 0 4.40476600 3.80106200 3.00542800 L

H-H_ 0 4.44315400 3.12017600 3.72701500 L

O-O_3 0 1.36123900 3.51539200 3.32055700 L

H-H_ 0 1.56623900 2.70390000 2.78675900 L

O-O_3 0 -1.40759400 3.65876000 2.54575600 L

H-H_ 0 -1.21382100 2.75942800 2.91929500 L

O-O_3 0 -3.74494800 5.15286500 1.82450400 L

H-H_ 0 -4.58214100 5.30302100 2.33623800 L

O-O_3 0 -5.68389400 3.16337000 1.05477500 L

H-H_ 0 -5.39721300 2.93607900 1.97784800 L

O-O_3 0 -7.43851300 0.69095000 -0.58413500 L

H-H_ 0 -6.86275500 0.98170600 0.16932000 L

O-O_3 0 -4.78147000 1.66610200 -1.24870700 L

O-O_3 0 -3.74321100 -2.30186300 -1.21138500 L

O-O_3 0 0.11230400 -4.26598300 -0.42742300 L

O-O_3 0 4.14905800 -2.72528400 -0.93585400 L

O-O_3 0 5.43537400 1.00820500 -0.02813100 L

O-O_3 0 2.49910800 3.92103000 0.74070100 L

O-O_3 0 -1.81030200 4.40859800 -0.17855400 L

O-O_3 0 -4.98165500 0.47696300 -3.25035300 L

O-O_3 0 -3.11536500 -4.32268900 -2.19701000 L

O-O_3 0 1.80289700 -5.51317800 -1.45650900 L

O-O_3 0 5.65909000 -1.76267300 -2.41914300 L

O-O_3 0 5.72843700 3.17694400 -0.84633200 L

O-O_3 0 1.42111000 5.99038700 0.61383100 L

O-O_3 0 -3.37506600 5.04131000 -1.77932800 L

O-O_3 0 -5.18343000 4.21027400 -3.84839800 L

H-H_ 0 -5.33258600 4.09973200 -4.82322700 L

O-O_3 0 -4.09464000 -1.68058100 -4.86180300 L

H-H_ 0 -4.53323200 -0.97604600 -5.40638800 L

O-O_3 0 -1.08434600 -6.00259000 -3.27159100 L

H-H_ 0 -0.54177800 -6.28524900 -4.05295700 L

O-O_3 0 4.11192900 -5.63165900 -3.07939200 L

H-H_ 0 4.52179600 -5.69693500 -3.98075400 L

O-O_3 0 6.78102200 0.62498200 -3.49926900 L

H-H_ 0 6.95590400 -0.16129600 -4.07933200 L

O-O_3 0 4.53114500 5.61958100 -1.73573500 L

H-H_ 0 5.46130100 5.51797500 -2.06700400 L

O-O_3 0 1.10452000 7.02370900 -1.91127600 L

H-H_ 0 1.29578400 6.34481900 -2.60985900 L

C-C_3 0 -0.97039700 -4.60749600 -3.20374900 L

H-H_ 0 -1.39421000 -4.15649700 -4.12888700 L

H-H_ 0 0.09495200 -4.31255200 -3.14844400 L

C-C_3 0 -1.73318300 -4.06118200 -1.99150400 L

H-H_ 0 -1.53609100 -2.96647000 -1.96409700 L

C-C_3 0 -1.23188100 -4.67411200 -0.65929100 L

H-H_ 0 -1.38294200 -5.77707900 -0.71775100 L

C-C_3 0 -2.09142700 -4.13977500 0.49975000 L

H-H_ 0 -1.85381900 -3.05773600 0.64319000 L

C-C_3 0 -3.58874400 -4.28761800 0.18761500 L

H-H_ 0 -3.83839700 -5.37566200 0.19115100 L

C-C_3 0 -3.91986700 -3.71215400 -1.20327000 L

H-H_ 0 -4.97656100 -4.00784200 -1.39990700 L

C-C_3 0 3.21366800 -4.55755400 -3.13786500 L

H-H_ 0 2.42695400 -4.77767500 -3.89342100 L

H-H_ 0 3.73876100 -3.63178700 -3.46025900 L

C-C_3 0 2.54716500 -4.34180900 -1.77323200 L

H-H_ 0 1.86667500 -3.46852200 -1.89705800 L

C-C_3 0 3.58237400 -3.99691000 -0.66713700 L

H-H_ 0 4.33641100 -4.81547400 -0.58556900 L

C-C_3 0 2.86102800 -3.88762500 0.68701300 L

H-H_ 0 2.22877000 -2.96870400 0.66645600 L

C-C_3 0 1.95897400 -5.11100600 0.92629700 L

H-H_ 0 2.61249300 -6.00552800 1.03299000 L

C-C_3 0 1.02905100 -5.34764200 -0.27898800 L

H-H_ 0 0.53016800 -6.32057500 -0.07000200 L

C-C_3 0 5.44630900 0.52831500 -3.07737700 L

H-H_ 0 4.79450800 0.22392400 -3.92837500 L

H-H_ 0 5.10169700 1.52873900 -2.75621700 L

C-C_3 0 5.29465700 -0.47901800 -1.92645100 L

H-H_ 0 4.21275300 -0.46800100 -1.66186100 L

C-C_3 0 6.12575000 -0.04984500 -0.67719100 L

H-H_ 0 7.15440700 0.20769400 -1.01980600 L

C-C_3 0 6.24958900 -1.19749400 0.35002000 L

H-H_ 0 5.28690500 -1.27104400 0.90720600 L

C-C_3 0 6.51453100 -2.54745800 -0.32702300 L

H-H_ 0 7.53389100 -2.52144500 -0.78075900 L

C-C_3 0 5.48055500 -2.76987600 -1.44072100 L

H-H_ 0 5.71233000 -3.73742200 -1.94021400 L

C-C_3 0 3.91426200 4.36688900 -1.87238800 L

H-H_ 0 4.17125700 3.91684100 -2.85849600 L

H-H_ 0 2.81597900 4.50708300 -1.86150800 L

C-C_3 0 4.32824900 3.41171100 -0.74150400 L

H-H_ 0 3.76635100 2.46932900 -0.92693200 L

C-C_3 0 3.91705400 3.97163500 0.65036100 L

H-H_ 0 4.32300800 5.00860100 0.72813800 L

C-C_3 0 4.51777700 3.11327600 1.78630100 L

H-H_ 0 3.94164200 2.16118600 1.83962900 L

C-C_3 0 5.99950300 2.79223100 1.51287700 L

H-H_ 0 6.57805100 3.74118900 1.56990200 L

C-C_3 0 6.17299700 2.21753800 0.09771200 L

H-H_ 0 7.26843300 2.08051600 -0.04490600 L

C-C_3 0 -0.02134300 6.57145500 -1.20780300 L

H-H_ 0 -0.78251300 6.18849000 -1.92421200 L

H-H_ 0 -0.46242600 7.43338500 -0.66315600 L

C-C_3 0 0.37600800 5.47865900 -0.20510100 L

H-H_ 0 0.73362200 4.61084100 -0.80368200 L

C-C_3 0 -0.83785600 5.02487800 0.65097100 L

H-H_ 0 -1.23172900 5.89516400 1.22895800 L

C-C_3 0 -0.36203200 3.96820000 1.66114600 L

H-H_ 0 -0.06770300 3.05487700 1.09220600 L

C-C_3 0 0.85300200 4.49329300 2.44604500 L

H-H_ 0 0.51782200 5.35522900 3.06504100 L

C-C_3 0 1.94780700 5.00147400 1.48479100 L

H-H_ 0 2.70159800 5.50372900 2.13375900 L

C-C_3 0 -3.95676100 3.59255800 -3.56973900 L

H-H_ 0 -3.14909400 4.10934400 -4.13559700 L

H-H_ 0 -3.96327300 2.54212100 -3.91584300 L

C-C_3 0 -3.63636500 3.67366100 -2.07118000 L

H-H_ 0 -2.71421100 3.06555000 -1.92564800 L

C-C_3 0 -4.77352300 3.08507200 -1.17057200 L

H-H_ 0 -5.75127700 3.54584900 -1.44294500 L

C-C_3 0 -4.52052500 3.43199900 0.31430100 L

H-H_ 0 -3.68193000 2.79741200 0.68441300 L

C-C_3 0 -4.11676500 4.90176400 0.49425700 L

H-H_ 0 -4.97660800 5.55574800 0.21152500 L

C-C_3 0 -2.94723100 5.22709500 -0.44361300 L

H-H_ 0 -2.72059000 6.31251700 -0.33817100 L

C-C_3 0 -3.25706700 -1.02370900 -3.94744500 L

H-H_ 0 -2.71781900 -0.18808300 -4.45032700 L

H-H_ 0 -2.48643000 -1.73540600 -3.59938100 L

C-C_3 0 -4.05369400 -0.47557100 -2.75210000 L

H-H_ 0 -3.30393000 0.02285800 -2.09657900 L

C-C_3 0 -4.74565100 -1.61880100 -1.95311800 L

H-H_ 0 -5.27363400 -2.26795200 -2.69201600 L

C-C_3 0 -5.78960600 -1.06943200 -0.95200100 L

H-H_ 0 -5.23951200 -0.69323900 -0.06479800 L

C-C_3 0 -6.61302500 0.08883800 -1.55030700 L

H-H_ 0 -7.27286200 -0.32873700 -2.34315900 L

C-C_3 0 -5.68651300 1.12267100 -2.20673000 L

H-H_ 0 -6.33928000 1.89063600 -2.67933700 L

C-C_R 0 0.18605800 -0.88115700 2.56452600 H

C-C_R 0 1.53324500 -1.05509200 2.89530100 H

C-C_R 0 2.49868500 -0.51374200 2.06569800 H

C-C_R 0 2.13788000 0.17274900 0.89364300 H

C-C_R 0 0.80665700 0.36581700 0.58861000 H

C-C_R 0 -0.19171100 -0.14288000 1.43693500 H

H-H_ 0 1.79400100 -1.60618700 3.79125200 H

H-H_ 0 3.54364800 -0.62723900 2.32894300 H

H-H_ 0 2.90222800 0.56041100 0.23512700 H

H-H_ 0 0.49813000 0.92082500 -0.28980700 H

C-C_R 0 -1.60435000 0.11863000 1.22097100 H

C-C_R 0 -2.51064100 -0.44692000 2.21299500 H

C-C_R 0 -2.07715900 -1.26677100 3.21632000 H

C-C_R 0 -2.87707100 -2.03639400 4.17431200 H

C-C_R 0 -2.23333600 -2.80232500 5.16462900 H

C-C_R 0 -4.28251000 -2.07611100 4.10421000 H

C-C_R 0 -2.96842000 -3.58117400 6.04966500 H

C-C_R 0 -5.00822700 -2.86326900 4.99209100 H

H-H_ 0 -4.80100600 -1.50053700 3.35227600 H

C-C_R 0 -4.35984600 -3.61850300 5.96728600 H

H-H_ 0 -2.45129200 -4.16506800 6.80424100 H

H-H_ 0 -6.09052900 -2.88630800 4.91698800 H

H-H_ 0 -4.93222200 -4.23143000 6.65599700 H

O-O_R 0 -2.07461800 0.78412900 0.29071900 H

O-O_R 0 -3.80933000 -0.12698300 2.02395400 H

H-H_ 0 -3.80098400 0.45738900 1.23973600 H

O-O_R 0 -0.72894300 -1.45462000 3.38769500 H

H-H_ 0 -1.15370800 -2.79073600 5.23234300 H

3-hydroxyflavone with ϒ-cyclodextrin 1:1 inclusion complex

ONIOM: gridpoint 1 method: low system: model energy: 0.128470829976
ONIOM: gridpoint 2 method: high system: model energy: -803.487287405905
ONIOM: gridpoint 3 method: low system: real energy: 0.439416617682
ONIOM: extrapolated energy = -803.176341618199
Sum of electronic and thermal Free Energies= -801.513753

O-O_3 0 5.91343900 2.30009500 2.53939100 M

H-H_ 0 6.85010600 2.25899100 2.86581900 M

O-O_3 0 4.13545000 4.48449700 2.21806600 M

H-H_ 0 3.14434800 4.49159500 2.25079200 M

O-O_3 0 6.38408800 3.14980000 -0.98289700 M

O-O_3 0 5.88555600 5.42088800 -2.57827400 M

H-H_ 0 5.84439200 5.84394800 -3.47497100 M

O-O_3 0 3.07013100 4.66802000 -0.46107300 M

O-O_3 0 3.21543700 7.02324500 1.14775800 M

H-H_ 0 3.03416100 7.88189100 1.61162500 M

O-O_3 0 0.38001900 7.29504400 1.54979400 M

H-H_ 0 0.77732800 7.12159500 2.44302800 M

O-O_3 0 1.86454200 5.89036800 -2.03233200 M

O-O_3 0 -0.63996400 6.33102000 -3.37446200 M

H-H_ 0 0.13168900 6.67781000 -3.89357200 M

O-O_3 0 -1.13702100 5.31646500 -0.00415200 M

O-O_3 0 -1.78662700 5.51972800 2.69163000 M

H-H_ 0 -2.22454500 5.50186700 3.58223700 M

O-O_3 0 -4.05799400 3.78423300 2.70672800 M

H-H_ 0 -4.01835800 2.79272600 2.72919200 M

O-O_3 0 -3.24237800 6.14948200 -0.59551000 M

O-O_3 0 -4.10232200 5.60166100 -3.14667200 M

H-H_ 0 -3.50242900 4.92781700 -3.56115700 M

O-O_3 0 -4.84540800 2.90998400 0.08379800 M

O-O_3 0 -6.76020800 3.01255500 2.21224400 M

H-H_ 0 -7.49583800 2.81450100 2.84857400 M

O-O_3 0 -7.05926800 0.14963200 2.42634800 M

H-H_ 0 -6.69253900 0.48016500 3.28772500 M

O-O_3 0 -6.49534800 1.93080100 -1.24066800 M

O-O_3 0 -7.47853000 -0.39660500 -2.57977500 M

H-H_ 0 -7.89159900 0.43650500 -2.92697100 M

O-O_3 0 -5.48563100 -1.21651300 0.34528000 M

O-O_3 0 -5.20932100 -2.18592000 2.95528600 M

H-H_ 0 -6.05653300 -2.17445000 3.47252000 M

O-O_3 0 -3.39591900 -4.31821600 2.41026200 M

H-H_ 0 -2.95444200 -3.66429400 3.01292700 M

O-O_3 0 -6.30481000 -3.28856200 -0.35649200 M

O-O_3 0 -6.11016400 -3.81981800 -3.05602000 M

H-H_ 0 -5.51217100 -3.20564900 -3.55678900 M

O-O_3 0 -2.92125400 -4.74163900 -0.36205900 M

O-O_3 0 -3.08062100 -6.95487400 1.50528900 M

H-H_ 0 -2.85615800 -7.74114800 2.06810600 M

O-O_3 0 -0.25009200 -7.21518000 1.86118000 M

H-H_ 0 -0.13687500 -8.12690000 1.48428400 M

O-O_3 0 -1.84064700 -6.18454100 -1.82910400 M

O-O_3 0 0.55197200 -6.90561800 -3.21827300 M

H-H_ 0 -0.26067000 -7.24877500 -3.67337200 M

O-O_3 0 1.25261300 -5.48433000 0.02789800 M

O-O_3 0 2.13846300 -5.62169800 2.74185700 M

H-H_ 0 1.44282100 -4.95103700 2.51538700 M

O-O_3 0 4.34613200 -3.80530300 2.49381700 M

H-H_ 0 5.04416600 -4.46802000 2.73727000 M

O-O_3 0 3.31915600 -6.32906900 -0.65775300 M

O-O_3 0 5.75662100 -6.01913600 -2.03705400 M

H-H_ 0 6.27062900 -6.05666600 -2.88502200 M

O-O_3 0 4.91621400 -3.03700000 -0.20820400 M

O-O_3 0 7.07489400 -3.11192400 1.66346900 M

H-H_ 0 7.84981300 -2.87011100 2.23475200 M

O-O_3 0 7.37965900 -0.25760300 1.87718500 M

H-H_ 0 8.28748300 -0.15148600 1.48904100 M

O-O_3 0 6.35436300 -2.01896300 -1.72832600 M

O-O_3 0 7.05641400 0.22779300 -3.27145400 M

H-H_ 0 7.32728200 1.13576200 -2.97526800 M

O-O_3 0 5.65274400 1.15434400 -0.01281600 M

C-C_3 0 6.42460000 2.33522000 0.17676200 M

H-H_ 0 7.49579500 2.11816600 0.39589700 M

C-C_3 0 5.91749400 3.12130200 1.40035300 M

H-H_ 0 6.59154800 3.99602900 1.56710400 M

C-C_3 0 4.50242600 3.64914400 1.15089500 M

H-H_ 0 3.80727700 2.77513100 1.09898000 M

C-C_3 0 4.43946400 4.42697800 -0.17845600 M

H-H_ 0 5.04026900 5.35888100 -0.05044600 M

C-C_3 0 5.05716700 3.55040700 -1.30184300 M

H-H_ 0 4.40063200 2.66315300 -1.44102900 M

C-C_3 0 5.09420200 4.26703300 -2.65659700 M

H-H_ 0 5.52581600 3.57577500 -3.41491600 M

H-H_ 0 4.06432400 4.52446200 -2.98649700 M

C-C_3 0 2.80714200 5.96974400 -0.97652400 M

H-H_ 0 3.71411700 6.45001900 -1.40789900 M

C-C_3 0 2.26999400 6.90584600 0.11668300 M

H-H_ 0 2.09211800 7.90555600 -0.34811800 M

C-C_3 0 0.93906400 6.36178000 0.66151700 M

H-H_ 0 1.13092200 5.40006500 1.19176200 M

C-C_3 0 -0.04980400 6.08628800 -0.49471000 M

H-H_ 0 -0.37213100 7.05869700 -0.93897500 M

C-C_3 0 0.65592200 5.27131600 -1.61233400 M

H-H_ 0 0.85684700 4.24161700 -1.23565800 M

C-C_3 0 -0.24621900 5.09342100 -2.84385400 M

H-H_ 0 0.28883100 4.49514300 -3.61658300 M

H-H_ 0 -1.15060500 4.51857700 -2.56813900 M

C-C_3 0 -2.23939400 6.11647200 0.40785500 M

H-H_ 0 -1.95029800 7.17273500 0.61315600 M

C-C_3 0 -2.81080700 5.58321300 1.73258900 M

H-H_ 0 -3.60522800 6.28937200 2.07453500 M

C-C_3 0 -3.42702300 4.19354000 1.52096200 M

H-H_ 0 -2.60825000 3.47953600 1.25802000 M

C-C_3 0 -4.44581300 4.24134900 0.36798700 M

H-H_ 0 -5.28385900 4.90316700 0.69385700 M

C-C_3 0 -3.75577800 4.85440900 -0.87774300 M

H-H_ 0 -2.94136900 4.17158300 -1.20143400 M

C-C_3 0 -4.72684600 4.97069700 -2.06098300 M

H-H_ 0 -5.08823400 3.96504400 -2.37162500 M

H-H_ 0 -5.60784200 5.57759800 -1.76179400 M

C-C_3 0 -6.24896200 2.76403900 -0.12284700 M

H-H_ 0 -6.75627900 3.73032600 -0.34672000 M

C-C_3 0 -6.92882100 2.15829600 1.11132900 M

H-H_ 0 -8.01613300 2.04575400 0.88272100 M

C-C_3 0 -6.32835800 0.77296000 1.40197400 M

H-H_ 0 -5.26309400 0.89360900 1.71454000 M

C-C_3 0 -6.36041500 -0.11672700 0.14037200 M

H-H_ 0 -7.41749100 -0.41748200 -0.05502000 M

C-C_3 0 -5.86305800 0.66628200 -1.10680800 M

H-H_ 0 -4.75954000 0.79584900 -1.03422600 M

C-C_3 0 -6.11354800 -0.12676200 -2.39887000 M

H-H_ 0 -5.56376300 -1.08539900 -2.36059000 M

H-H_ 0 -5.71756800 0.44221800 -3.27038000 M

C-C_3 0 -6.16080500 -2.40290100 0.74400100 M

H-H_ 0 -7.18084700 -2.20411000 1.14711400 M

C-C_3 0 -5.39526400 -3.08601800 1.89378600 M

H-H_ 0 -5.98273900 -3.96926300 2.24304500 M

C-C_3 0 -4.03151200 -3.58036300 1.39886700 M

H-H_ 0 -3.39942200 -2.70151700 1.11996300 M

C-C_3 0 -4.21300700 -4.46259100 0.15353900 M

H-H_ 0 -4.78049100 -5.37453600 0.45758100 M

C-C_3 0 -5.04595600 -3.67851700 -0.89388300 M

H-H_ 0 -4.46384100 -2.78826900 -1.21875600 M

C-C_3 0 -5.30070800 -4.52180500 -2.15117500 M

H-H_ 0 -4.33813400 -4.79613500 -2.63980000 M

H-H_ 0 -5.82416000 -5.46029100 -1.86944000 M

C-C_3 0 -2.73522200 -6.10489500 -0.73226800 M

H-H_ 0 -3.68605100 -6.57452600 -1.07212000 M

C-C_3 0 -2.18327300 -6.95421800 0.42649500 M

H-H_ 0 -2.05872800 -7.99667700 0.04683200 M

C-C_3 0 -0.81398500 -6.40511100 0.85975300 M

H-H_ 0 -0.96579900 -5.39231100 1.29443000 M

C-C_3 0 0.13798200 -6.27764400 -0.35280900 M

H-H_ 0 0.42136900 -7.29916300 -0.70270800 M

C-C_3 0 -0.58867600 -5.57563400 -1.53438900 M

H-H_ 0 -0.73217600 -4.50084900 -1.28303000 M

C-C_3 0 0.26503400 -5.59341000 -2.81271700 M

H-H_ 0 -0.26723100 -5.04660400 -3.62469500 M

H-H_ 0 1.21849600 -5.05818800 -2.63963900 M

C-C_3 0 2.38199800 -6.26387500 0.40524400 M

H-H_ 0 2.11211900 -7.31153600 0.67274600 M

C-C_3 0 3.04625100 -5.68330800 1.66942100 M

H-H_ 0 3.86673000 -6.37522700 1.96352400 M

C-C_3 0 3.65797600 -4.30152900 1.37284100 M

H-H_ 0 2.83181500 -3.59056000 1.14632700 M

C-C_3 0 4.58098400 -4.37048000 0.14066300 M

H-H_ 0 5.46370300 -4.99687900 0.41259200 M

C-C_3 0 3.79944500 -5.04078800 -1.02424100 M

H-H_ 0 2.95474200 -4.37253800 -1.30370300 M

C-C_3 0 4.64975200 -5.19775700 -2.28996300 M

H-H_ 0 4.02307700 -5.65904300 -3.08604400 M

H-H_ 0 4.98117700 -4.20303000 -2.65913700 M

C-C_3 0 6.27669000 -2.86588700 -0.59461200 M

H-H_ 0 6.76166800 -3.82315300 -0.89124400 M

C-C_3 0 7.11172000 -2.26445500 0.54596300 M

H-H_ 0 8.16266700 -2.16960500 0.18174200 M

C-C_3 0 6.56794700 -0.87025400 0.90657600 M

H-H_ 0 5.55403200 -0.99603500 1.34892700 M

C-C_3 0 6.44234900 0.02203800 -0.34976600 M

H-H_ 0 7.46662200 0.27591500 -0.71173400 M

C-C_3 0 5.73671300 -0.76010900 -1.49209900 M

H-H_ 0 4.66304300 -0.89613600 -1.22668600 M

C-C_3 0 5.74538500 0.02165100 -2.81420800 M

H-H_ 0 5.20040800 -0.57136200 -3.57945100 M

H-H_ 0 5.19731700 0.98113000 -2.71211300 M

C-C_R 0 0.17027700 1.35031900 1.97195500 H

C-C_R 0 0.33668000 2.66476300 2.41850100 H

C-C_R 0 1.08439200 2.89502800 3.55897400 H

C-C_R 0 1.67582300 1.82764100 4.25836800 H

C-C_R 0 1.49980500 0.53076500 3.81554600 H

C-C_R 0 0.73541800 0.27189300 2.66278400 H

H-H_ 0 -0.12560500 3.46987100 1.86262600 H

H-H_ 0 1.21538300 3.91228000 3.91278500 H

H-H_ 0 2.26526100 2.02543300 5.14678400 H

H-H_ 0 1.93701300 -0.31281900 4.33724800 H

C-C_R 0 0.50072700 -1.07432400 2.16015100 H

C-C_R 0 -0.30025500 -1.16796300 0.94339800 H

C-C_R 0 -0.78954200 -0.06314100 0.30347300 H

C-C_R 0 -1.52461300 0.01208000 -0.96296400 H

C-C_R 0 -1.77135300 1.26936400 -1.54768100 H

C-C_R 0 -1.96712200 -1.14135000 -1.63648600 H

C-C_R 0 -2.41730400 1.36715000 -2.77340100 H

C-C_R 0 -2.61253100 -1.03206000 -2.86365900 H

H-H_ 0 -1.79983200 -2.11594100 -1.20351800 H

C-C_R 0 -2.83229500 0.21630200 -3.44271300 H

H-H_ 0 -2.59472500 2.34514900 -3.20875800 H

H-H_ 0 -2.94551000 -1.93272600 -3.36927800 H

H-H_ 0 -3.33111200 0.29311900 -4.40329700 H

O-O_R 0 0.92984500 -2.11555500 2.67189500 H

O-O_R 0 -0.48478500 -2.43001500 0.49425300 H

H-H_ 0 -0.00362400 -2.99582300 1.13161900 H

O-O_R 0 -0.56167800 1.17764100 0.84252400 H

H-H_ 0 -1.44289800 2.16726800 -1.04190100 H

3-hydroxyflavone with ϒ-cyclodextrin 1:1 Capped with primary side

ONIOM: gridpoint 1 method: low system: model energy: 0.119495069972
ONIOM: gridpoint 2 method: high system: model energy: -803.486976670345
ONIOM: gridpoint 3 method: low system: real energy: 0.440611408401
ONIOM: extrapolated energy = -803.165860331917
Sum of electronic and thermal Free Energies= -801.504527

```
O-O_3 0 -3.89320300 -3.68432700 -3.78745900 M
H-H_ 0 -4.15972100 -4.54030100 -4.21382700 M
O-O_3 0 -5.14393900 -1.14819200 -3.66817900 M
H-H_ 0 -4.70322200 -0.26342100 -3.57762400 M
O-O_3 0 -5.66295700 -3.87997500 -0.59912100 M
O-O_3 0 -7.90768200 -2.58636400 0.46213300 M
H-H_ 0 -8.51535600 -2.41595400 1.22792700 M
O-O_3 0 -5.52758700 -0.21491400 -0.96444200 M
O-O_3 0 -7.21157700 0.82325500 -3.01801800 M
H-H_ 0 -7.76598600 1.39919200 -3.60642600 M
O-O_3 0 -6.16267400 3.51570200 -2.94022800 M
H-H_ 0 -5.98331200 3.14799200 -3.84482700 M
O-O_3 0 -6.49675600 1.30166600 0.51271600 M
O-O_3 0 -6.19533500 3.64192300 2.14350400 M
H-H_ 0 -6.95347900 3.07369900 2.43951600 M
O-O_3 0 -4.16460300 3.80342800 -0.81501900 M
O-O_3 0 -3.62193300 4.86775800 -3.34076800 M
H-H_ 0 -3.32461700 5.40051700 -4.12384100 M
O-O_3 0 -0.98647900 5.87801100 -2.78351800 M
H-H_ 0 -0.88701900 5.17082100 -3.47313000 M
O-O_3 0 -3.91743000 5.92316400 0.13721700 M
O-O_3 0 -3.40965700 6.00831800 2.83634700 M
H-H_ 0 -3.17402300 5.12127100 3.21461300 M
O-O_3 0 -0.25157900 5.61677700 -0.03811000 M
O-O_3 0 0.73953100 7.78044600 -1.65113500 M
H-H_ 0 1.30161900 8.46708300 -2.09607900 M
O-O_3 0 3.38285100 6.71904700 -2.00606300 M
H-H_ 0 2.95883000 6.75310800 -2.90314200 M
O-O_3 0 1.36092100 6.15861100 1.54613400 M
O-O_3 0 3.79310600 5.38162700 2.91534300 M
```

H-H_ 0 3.28219800 6.07840300 3.40384500 M

O-O_3 0 3.83103700 4.29158800 -0.43619400 M

O-O_3 0 4.66584200 4.12474300 -3.10751200 M

H-H_ 0 4.98619400 4.99474500 -3.46235700 M

O-O_3 0 5.93006500 1.56067000 -2.97981000 M

H-H_ 0 5.18572300 1.54485100 -3.63656500 M

O-O_3 0 6.05828900 4.11656200 0.26916200 M

O-O_3 0 6.44250400 3.30072500 2.86699300 M

H-H_ 0 5.59633800 2.96018400 3.25927600 M

O-O_3 0 5.98489100 0.48568800 -0.32978900 M

O-O_3 0 7.91165100 -0.37138000 -2.25558900 M

H-H_ 0 8.47582900 -0.92277100 -2.85810800 M

O-O_3 0 6.84797700 -3.03348200 -2.56728700 M

H-H_ 0 7.60078900 -3.56842600 -2.20244600 M

O-O_3 0 6.79824500 -1.14659400 1.11658900 M

O-O_3 0 6.33542100 -3.61731900 2.50410200 M

H-H_ 0 7.05835200 -3.07900600 2.91986200 M

O-O_3 0 4.65371100 -3.51989200 -0.68300200 M

O-O_3 0 4.38200800 -4.59747500 -3.31539800 M

H-H_ 0 4.14898900 -3.64078300 -3.18982000 M

O-O_3 0 1.67843800 -5.52389000 -3.04579100 M

H-H_ 0 1.91338400 -6.47327800 -3.21617600 M

O-O_3 0 4.29297000 -5.65285400 0.19477800 M

O-O_3 0 2.73513800 -7.44242300 1.71247000 M

H-H_ 0 2.47753200 -7.82125200 2.59270300 M

O-O_3 0 0.65862600 -5.36576100 -0.36591500 M

O-O_3 0 -0.20502500 -7.49514500 -2.08904600 M

H-H_ 0 -0.75329600 -8.11934500 -2.63229200 M

O-O_3 0 -2.76689200 -6.37404300 -2.68670100 M

H-H_ 0 -3.32076800 -7.09612300 -2.28907700 M

O-O_3 0 -1.07675500 -5.93459800 1.07894000 M

O-O_3 0 -3.52903300 -5.23321700 2.33292900 M

H-H_ 0 -4.41352000 -5.02698300 1.93209000 M

O-O_3 0 -3.36629300 -3.99911400 -1.04839300 M

C-C_3 0 -4.67800300 -4.20647400 -1.56380100 M

H-H_ 0 -4.85772300 -5.26368000 -1.86098000 M

C-C_3 0 -4.88534700 -3.37659900 -2.84248400 M

H-H_ 0 -5.89392700 -3.60695400 -3.26286100 M

C-C_3 0 -4.82812600 -1.88321900 -2.51383000 M

H-H_ 0 -3.78937900 -1.63822100 -2.18097500 M

C-C_3 0 -5.81461300 -1.54074600 -1.38164700 M

H-H_ 0 -6.84575700 -1.66654300 -1.78982700 M

C-C_3 0 -5.58969800 -2.52001900 -0.19456500 M

H-H_ 0 -4.59530500 -2.30255900 0.24684600 M

C-C_3 0 -6.61345500 -2.31567600 0.92620300 M

H-H_ 0 -6.36953400 -3.00627600 1.76452500 M

H-H_ 0 -6.55533000 -1.27840600 1.31439300 M

C-C_3 0 -6.68385600 0.56959900 -0.68657900 M

H-H_ 0 -7.59650700 -0.04958300 -0.53242700 M

C-C_3 0 -6.98389000 1.54373400 -1.83475600 M

H-H_ 0 -7.89964500 2.12116500 -1.56064900 M

C-C_3 0 -5.80930100 2.52057400 -2.01422500 M

H-H_ 0 -4.91850000 1.95646600 -2.38236500 M

C-C_3 0 -5.43587200 3.18543000 -0.67234800 M

H-H_ 0 -6.24892600 3.89428500 -0.38554000 M

C-C_3 0 -5.33670000 2.12048900 0.45330800 M

H-H_ 0 -4.43054200 1.49694400 0.28072400 M

C-C_3 0 -5.13833700 2.77412200 1.83043300 M

H-H_ 0 -5.04438200 1.98721400 2.61242300 M

H-H_ 0 -4.19172300 3.34702500 1.83905200 M

C-C_3 0 -4.24309000 5.20624700 -1.04335400 M

H-H_ 0 -5.25838800 5.53683800 -1.36371300 M

C-C_3 0 -3.30536700 5.61255800 -2.19297900 M

H-H_ 0 -3.45532200 6.70158300 -2.39065800 M

C-C_3 0 -1.84296800 5.37082700 -1.79280400 M

H-H_ 0 -1.67440500 4.27242900 -1.67207800 M

C-C_3 0 -1.53558200 6.05731300 -0.45166100 M

H-H_ 0 -1.59976100 7.16039000 -0.61236700 M

C-C_3 0 -2.60165400 5.62889100 0.59272300 M

H-H_ 0 -2.48743900 4.53949800 0.79116400 M

C-C_3 0 -2.39003800 6.34547500 1.93430800 M

H-H_ 0 -1.40003200 6.07946800 2.36887300 M

H-H_ 0 -2.41321800 7.44489800 1.77641200 M

C-C_3 0 0.57546900 6.65622300 0.47637800 M

H-H_ 0 -0.02111900 7.49791500 0.89608400 M

C-C_3 0 1.50209100 7.23939700 -0.60423800 M

H-H_ 0 2.11060100 8.04552800 -0.12743700 M

C-C_3 0 2.44698300 6.14611300 -1.12980100 M

H-H_ 0 1.84798800 5.37007600 -1.66577400 M

C-C_3 0 3.18614600 5.46337400 0.04332000 M

H-H_ 0 3.89600600 6.19408000 0.50065900 M

C-C_3 0 2.16284300 5.05608600 1.14115300 M

H-H_ 0 1.52227400 4.23266200 0.74903100 M

C-C_3 0 2.85103900 4.48434700 2.39060800 M

H-H_ 0 3.35859900 3.53478300 2.14036800 M

H-H_ 0 2.08421600 4.23767700 3.16037000 M

C-C_3 0 5.19511200 4.51440700 -0.78338500 M

H-H_ 0 5.41160800 5.58634000 -0.99667500 M

C-C_3 0 5.54731900 3.75809300 -2.07768100 M

H-H_ 0 6.59594100 4.00957700 -2.36773500 M

C-C_3 0 5.46236500 2.24418100 -1.84571200 M

H-H_ 0 4.39817700 1.96420100 -1.65000200 M

C-C_3 0 6.30227900 1.83725300 -0.62316200 M

H-H_ 0 7.37604500 1.99951500 -0.88252300 M

C-C_3 0 5.91402500 2.73724900 0.58015000 M

H-H_ 0 4.86425400 2.51062100 0.85534400 M

C-C_3 0 6.77487700 2.43480200 1.81518600 M

H-H_ 0 6.63904200 1.38091100 2.14380100 M

H-H_ 0 7.84727500 2.58155400 1.56531500 M

C-C_3 0 7.11303800 -0.31315400 0.01413500 M

H-H_ 0 7.98987600 0.29748000 0.32937300 M

C-C_3 0 7.55987300 -1.18592400 -1.16827000 M

H-H_ 0 8.44835000 -1.77505000 -0.83738800 M

C-C_3 0 6.42385200 -2.14905800 -1.55986900 M

H-H_ 0 5.58642300 -1.54471700 -1.97565700 M

C-C_3 0 5.89635400 -2.92736700 -0.33200200 M

H-H_ 0 6.67373700 -3.65978200 -0.00911200 M

C-C_3 0 5.66072400 -1.95971000 0.85837000 M

H-H_ 0 4.77382900 -1.32737800 0.63039100 M

C-C_3 0 5.31631600 -2.72055400 2.14957600 M

H-H_ 0 5.14085100 -1.99473800 2.97565000 M

H-H_ 0 4.37450800 -3.28587000 2.01751000 M

C-C_3 0 4.74913200 -4.91747300 -0.93021200 M

H-H_ 0 5.79152000 -5.24715800 -1.14776100 M

C-C_3 0 3.93839000 -5.30341600 -2.18276400 M

H-H_ 0 4.11323700 -6.38601000 -2.37231200 M

C-C_3 0 2.43154900 -5.08820500 -1.94165700 M

H-H_ 0 2.24531900 -3.99709300 -1.82115200 M

C-C_3 0 1.98200900 -5.79615200 -0.64808300 M

H-H_ 0 2.06917700 -6.89638900 -0.81323900 M

C-C_3 0 2.93282400 -5.36564300 0.50368600 M

H-H_ 0 2.79159800 -4.27535700 0.67882900 M

C-C_3 0 2.59323600 -6.05332500 1.83106900 M

H-H_ 0 3.28727100 -5.67813900 2.61629700 M

H-H_ 0 1.55848800 -5.79488000 2.14602500 M

C-C_3 0 -0.20756000 -6.41204500 0.06521300 M

H-H_ 0 0.35111900 -7.26375700 0.51442000 M

C-C_3 0 -1.04499400 -6.97147900 -1.09528100 M

H-H_ 0 -1.68665700 -7.78655100 -0.68241000 M

C-C_3 0 -1.94183100 -5.86013400 -1.67131200 M

H-H_ 0 -1.28929400 -5.08463300 -2.13123900 M

C-C_3 0 -2.77675100 -5.19158900 -0.54924100 M

H-H_ 0 -3.53272400 -5.92025700 -0.17072900 M

C-C_3 0 -1.84280500 -4.82003200 0.63824600 M

H-H_ 0 -1.17088400 -3.98967300 0.31982100 M

C-C_3 0 -2.62536700 -4.27805000 1.84261100 M

H-H_ 0 -1.90519600 -4.03254900 2.65210900 M

H-H_ 0 -3.13889500 -3.32973800 1.58176300 M

C-C_R 0 0.69362400 -0.42965800 3.21974500 H

C-C_R 0 1.74836100 -0.42225300 4.13893800 H

C-C_R 0 3.00328900 -0.03255600 3.70975100 H

C-C_R 0 3.21902500 0.34534000 2.37382800 H

C-C_R 0 2.17491100 0.33258900 1.46970000 H

C-C_R 0 0.88650200 -0.05680300 1.88313900 H

H-H_ 0 1.55864800 -0.71464800 5.16519200 H

H-H_ 0 3.82957100 -0.01994700 4.41262300 H

H-H_ 0 4.21362900 0.63330200 2.06049500 H

H-H_ 0 2.31797700 0.61801500 0.43374200 H

C-C_R 0 -0.25856100 -0.08401300 0.98025000 H

C-C_R 0 -1.52932300 -0.47885500 1.58094300 H

C-C_R 0 -1.63846100 -0.81370600 2.89964500 H

C-C_R 0 -2.83870100 -1.20906600 3.64535100 H

C-C_R 0 -2.70252400 -1.91956800 4.85144400 H

C-C_R 0 -4.12775700 -0.86450300 3.20602000 H

C-C_R 0 -3.82106600 -2.27457600 5.59487700 H

C-C_R 0 -5.23937400 -1.19609100 3.97456600 H

H-H_ 0 -4.25230100 -0.33588600 2.27180000 H

C-C_R 0 -5.09484200 -1.90589300 5.16459600 H

H-H_ 0 -3.69703200 -2.83368700 6.51661900 H

H-H_ 0 -6.22782500 -0.89353800 3.64648100 H

H-H_ 0 -5.96817100 -2.16899300 5.75245800 H

O-O_R 0 -0.22899400 0.19802700 -0.22345100 H

O-O_R 0 -2.58282100 -0.48925500 0.73247300 H

H-H_ 0 -2.21493300 -0.23079200 -0.13567900 H

O-O_R 0 -0.52133400 -0.80608700 3.69400600 H

H-H_ 0 -1.71575900 -2.19549600 5.20043000 H

3-hydroxyflavone with ϒ-cyclodextrin 1:1 Capped with secondary side

ONIOM: gridpoint 1 method: low system: model energy: 0.126995492053
ONIOM: gridpoint 2 method: high system: model energy: -803.486950816575
ONIOM: gridpoint 3 method: low system: real energy: 0.449751934845
ONIOM: extrapolated energy = -803.164194373783
Sum of electronic and thermal Free Energies= -801.502545

O-O_3 0 -6.68893900 -1.55954400 2.26732300 M

H-H_ 0 -7.64729200 -1.39969900 2.47119200 M

O-O_3 0 -5.09291100 -3.88537100 2.08888200 M

H-H_ 0 -4.11406400 -3.95432000 2.24076600 M

O-O_3 0 -6.87306600 -2.30495600 -1.30280000 M

O-O_3 0 -6.50420500 -4.67236400 -2.82362000 M

H-H_ 0 -6.43905500 -5.10988400 -3.71188900 M

O-O_3 0 -3.83251600 -4.22457500 -0.47671200 M

O-O_3 0 -3.98063200 -6.46689500 1.23720400 M

H-H_ 0 -3.83092400 -7.31615700 1.72879900 M

O-O_3 0 -1.14497000 -6.96159900 1.41116800 M

H-H_ 0 -1.45316600 -6.72385800 2.32446900 M

O-O_3 0 -2.82112300 -5.56693400 -2.09193200 M

O-O_3 0 -0.44867200 -6.19904100 -3.61353900 M

H-H_ 0 -1.27860600 -6.53337000 -4.04321100 M

O-O_3 0 0.40322200 -5.24589000 -0.35739200 M

O-O_3 0 1.44032000 -5.52619400 2.20344400 M

H-H_ 0 2.00481800 -5.56786300 3.01907600 M

O-O_3 0 3.95474400 -4.18710600 1.85242000 M

H-H_ 0 4.17174000 -3.22057100 1.80544700 M

O-O_3 0 2.27765100 -6.32321800 -1.25839900 M

O-O_3 0 2.76842600 -5.86927200 -3.91472200 M

H-H_ 0 2.22636900 -5.11384200 -4.26249200 M

O-O_3 0 4.37981000 -3.32848800 -0.85555200 M

O-O_3 0 6.65280400 -3.82893400 0.79848500 M

H-H_ 0 7.53620200 -3.81272100 1.25078800 M

O-O_3 0 7.51786500 -1.07160600 0.99613000 M

H-H_ 0 7.34441000 -1.44349700 1.89966800 M

O-O_3 0 5.82497000 -2.48127000 -2.47196000 M

O-O_3 0 6.75695800 -0.22053900 -3.95508300 M

H-H_ 0 6.93512700 -1.07477000 -4.42815500 M

O-O_3 0 5.77674000 0.67319900 -0.59975200 M

O-O_3 0 6.44722100 1.50244200 2.01491200 M

H-H_ 0 7.39916400 1.35419300 2.25431800 M

O-O_3 0 4.84587200 3.85577300 2.05364400 M

H-H_ 0 4.52631200 3.22329500 2.74921000 M

O-O_3 0 6.71022600 2.64523000 -1.44000900 M

O-O_3 0 5.97362000 3.45291300 -3.94893100 M

H-H_ 0 5.21513100 2.92021200 -4.30440800 M

O-O_3 0 3.63940800 4.44124300 -0.47546700 M

O-O_3 0 3.92725900 6.48521200 1.49006300 M

H-H_ 0 4.73362500 7.01185900 1.24895800 M

O-O_3 0 1.14858200 6.93656700 1.93362400 M

H-H_ 0 1.08623900 7.87341200 1.61038800 M

O-O_3 0 2.54879200 5.97088100 -1.85184900 M

O-O_3 0 0.09345600 6.80588900 -3.12609000 M

H-H_ 0 0.90135900 7.17631000 -3.56800900 M

O-O_3 0 -0.54903800 5.47523900 0.06643400 M

O-O_3 0 -1.50567800 5.58745500 2.74709200 M

H-H_ 0 -0.91046800 4.83233000 2.49982500 M

O-O_3 0 -3.96203600 4.14269200 2.36955300 M

H-H_ 0 -4.56341200 4.89137100 2.62126600 M

O-O_3 0 -2.47099600 6.60033400 -0.64606500 M

O-O_3 0 -4.85511500 6.68974000 -2.13870300 M

H-H_ 0 -5.31904900 6.82226900 -3.00581200 M

O-O_3 0 -4.56052100 3.57518000 -0.37175300 M

O-O_3 0 -6.76578400 4.00891800 1.40423700 M

H-H_ 0 -7.59461000 3.89565100 1.93860700 M

O-O_3 0 -7.52881500 1.24327500 1.59058700 M

H-H_ 0 -8.42619400 1.27824900 1.16699700 M

O-O_3 0 -6.08159500 2.81297300 -1.95979500 M

O-O_3 0 -7.08904400 0.72772600 -3.54919900 M

H-H_ 0 -7.53005900 -0.11672500 -3.27009700 M

O-O_3 0 -5.97942500 -0.43029800 -0.23098600 M

C-C_3 0 -6.92149300 -1.49450600 -0.14000000 M

H-H_ 0 -7.96993900 -1.13512600 -0.02409000 M

C-C_3 0 -6.64784200 -2.35176100 1.10999600 M

H-H_ 0 -7.42589700 -3.15014500 1.17516600 M

C-C_3 0 -5.27719000 -3.02433400 0.99477000 M

H-H_ 0 -4.49244300 -2.22955800 1.00005500 M

C-C_3 0 -5.18317600 -3.81385300 -0.32319100 M

H-H_ 0 -5.90605900 -4.66035600 -0.24647300 M

C-C_3 0 -5.58508900 -2.87861700 -1.49891100 M

H-H_ 0 -4.81205100 -2.08244800 -1.58303800 M

C-C_3 0 -5.59602400 -3.60548800 -2.84859000 M

H-H_ 0 -5.89663600 -2.88434500 -3.64147800 M

H-H_ 0 -4.57737400 -3.96970100 -3.09927600 M

C-C_3 0 -3.68298400 -5.55444100 -0.96670300 M

H-H_ 0 -4.64108400 -6.00458900 -1.31206300 M

C-C_3 0 -3.12265000 -6.47898800 0.12623300 M

H-H_ 0 -3.06605500 -7.51095500 -0.29693700 M

C-C_3 0 -1.70769800 -6.02401500 0.52981200 M

H-H_ 0 -1.77005800 -5.02452800 1.02556900 M

C-C_3 0 -0.81203300 -5.88889300 -0.72081400 M

H-H_ 0 -0.65570000 -6.90168600 -1.16531900 M

C-C_3 0 -1.54282900 -5.02887200 -1.78569000 M

H-H_ 0 -1.63857300 -3.98490100 -1.40764000 M

C-C_3 0 -0.74565900 -4.92967200 -3.09496100 M

H-H_ 0 -1.32291900 -4.33524800 -3.83987900 M

H-H_ 0 0.19846100 -4.38170800 -2.91938200 M

C-C_3 0 1.45678300 -6.17306300 -0.11141400 M

H-H_ 0 1.07591500 -7.18702500 0.15255200 M

C-C_3 0 2.29109100 -5.73561100 1.10522500 M

H-H_ 0 3.01272900 -6.55532800 1.33771600 M

C-C_3 0 3.07509600 -4.45471300 0.79271800 M

H-H_ 0 2.34491600 -3.61528700 0.68696300 M

C-C_3 0 3.86802000 -4.60875000 -0.51814500 M

H-H_ 0 4.66215000 -5.37363600 -0.34219000 M

C-C_3 0 2.90484000 -5.10270100 -1.62882400 M

H-H_ 0 2.14868600 -4.30940600 -1.81262500 M

C-C_3 0 3.64063700 -5.33148900 -2.95728100 M

H-H_ 0 4.07059800 -4.37817500 -3.33736000 M

H-H_ 0 4.47377600 -6.04973600 -2.80215400 M

C-C_3 0 5.71141700 -3.35481400 -1.36284700 M

H-H_ 0 6.00794500 -4.36261300 -1.73312800 M

C-C_3 0 6.73114600 -2.94543300 -0.28998400 M

H-H_ 0 7.74707400 -3.00095000 -0.75055200 M

C-C_3 0 6.46872300 -1.49942900 0.16689100 M

H-H_ 0 5.50401200 -1.46267400 0.72701200 M

C-C_3 0 6.35956200 -0.54483200 -1.04184700 M

H-H_ 0 7.37325300 -0.41851300 -1.49139900 M

C-C_3 0 5.44204400 -1.15471500 -2.13704900 M

H-H_ 0 4.38728900 -1.13356600 -1.77801600 M

C-C_3 0 5.46173200 -0.31485900 -3.42386000 M

H-H_ 0 5.08819700 0.70426700 -3.21322400 M

H-H_ 0 4.76921300 -0.76221100 -4.17340800 M

C-C_3 0 6.72844700 1.70830500 -0.37496200 M

H-H_ 0 7.77133100 1.32581500 -0.28389200 M

C-C_3 0 6.43439600 2.42235200 0.95593500 M

H-H_ 0 7.21126000 3.20654100 1.12541400 M

C-C_3 0 5.06499900 3.10571900 0.88698300 M

H-H_ 0 4.27331500 2.32624500 0.78594300 M

C-C_3 0 4.99150900 4.03007300 -0.33877400 M

H-H_ 0 5.70217900 4.87209300 -0.15867700 M

C-C_3 0 5.42804500 3.24190000 -1.60558300 M

H-H_ 0 4.65946200 2.46624100 -1.81832800 M

C-C_3 0 5.48613300 4.15660000 -2.83794700 M

H-H_ 0 4.47918600 4.56957600 -3.06837800 M

H-H_ 0 6.16981600 5.00885100 -2.63692000 M

C-C_3 0 3.48558600 5.81985200 -0.79798100 M

H-H_ 0 4.42910600 6.28642000 -1.16269000 M

C-C_3 0 3.02017600 6.62645900 0.42909400 M

H-H_ 0 2.95230900 7.70176100 0.13854400 M

C-C_3 0 1.62673800 6.14846200 0.87198200 M

H-H_ 0 1.71631500 5.10321800 1.24507900 M

C-C_3 0 0.63900600 6.15531300 -0.31748900 M

H-H_ 0 0.46176300 7.21048900 -0.63732400 M

C-C_3 0 1.28194000 5.41701300 -1.52189900 M

H-H_ 0 1.38188600 4.33510600 -1.27384000 M

C-C_3 0 0.39608600 5.48125700 -2.77657100 M

H-H_ 0 0.90901800 4.96821700 -3.62220000 M

H-H_ 0 -0.54763800 4.93234500 -2.60231300 M

C-C_3 0 -1.59093900 6.37046600 0.44275900 M

H-H_ 0 -1.20116500 7.36282500 0.76759300 M

C-C_3 0 -2.36739000 5.83333100 1.66280600 M

H-H_ 0 -3.08386700 6.62509200 1.97620000 M

C-C_3 0 -3.17187700 4.57411300 1.28973300 M

H-H_ 0 -2.45286600 3.75878400 1.05419000 M

C-C_3 0 -4.03398600 4.82780100 0.03747800 M

H-H_ 0 -4.81857700 5.57467500 0.30583300 M

C-C_3 0 -3.11481300 5.40590000 -1.07327500 M

H-H_ 0 -2.36580900 4.62714400 -1.33441500 M

C-C_3 0 -3.87291100 5.71701400 -2.36859100 M

H-H_ 0 -3.14825800 6.09698800 -3.12338400 M

H-H_ 0 -4.33110500 4.79193600 -2.78101300 M

C-C_3 0 -5.91325700 3.63406100 -0.81659200 M

H-H_ 0 -6.21622800 4.65988500 -1.12441100 M

C-C_3 0 -6.88907800 3.17925100 0.27947100 M

H-H_ 0 -7.92356400 3.25665400 -0.13265100 M

C-C_3 0 -6.59515100 1.71668900 0.65253600 M

H-H_ 0 -5.59235700 1.68550900 1.12928000 M

C-C_3 0 -6.56423000 0.81187500 -0.59956800 M

H-H_ 0 -7.60080600 0.72714000 -1.00192300 M

C-C_3 0 -5.69423200 1.46735800 -1.70836400 M

H-H_ 0 -4.62412600 1.42161500 -1.40074800 M

C-C_3 0 -5.78337400 0.69793200 -3.03462800 M

H-H_ 0 -5.10898900 1.18388300 -3.77167700 M

H-H_ 0 -5.42032600 -0.34402200 -2.91488500 M

C-C_R 0 -1.11618300 -0.90706600 3.19310700 H

C-C_R 0 -2.13452900 -1.61717300 3.83858700 H

C-C_R 0 -3.44433800 -1.21044100 3.66368100 H

C-C_R 0 -3.75209300 -0.11418800 2.83960300 H

C-C_R 0 -2.74565900 0.55948600 2.17659600 H

C-C_R 0 -1.40333800 0.17402500 2.35006400 H

H-H_ 0 -1.87585800 -2.45561700 4.47479900 H

H-H_ 0 -4.24155500 -1.74371100 4.17064000 H

H-H_ 0 -4.78262300 0.19809900 2.73285300 H

H-H_ 0 -2.95883300 1.39544900 1.52167800 H

C-C_R 0 -0.29423200 0.87112700 1.71295000 H

C-C_R 0 1.04063400 0.39502000 2.06519000 H

C-C_R 0 1.24290100 -0.64574700 2.92766600 H

C-C_R 0 2.50784900 -1.18225300 3.44290500 H

C-C_R 0 2.49765300 -2.35800200 4.21779400 H

C-C_R 0 3.73598600 -0.52801400 3.23213600 H

C-C_R 0 3.67122500 -2.85786900 4.76810300 H

C-C_R 0 4.90265100 -1.03091200 3.79953500 H

H-H_ 0 3.77202900 0.37338500 2.63827300 H

C-C_R 0 4.88040100 -2.19404000 4.56593600 H

H-H_ 0 3.64021900 -3.76905300 5.35659300 H

H-H_ 0 5.83847500 -0.50576700 3.64762000 H

H-H_ 0 5.79679900 -2.57993300 5.00033700 H

O-O_R 0 -0.39857600 1.82622700 0.93367900 H

O-O_R 0 2.05028100 1.08597900 1.48993400 H

H-H_ 0 1.61063100 1.76911400 0.94397100 H

O-O_R 0 0.15648900 -1.30739100 3.44155700 H

H-H_ 0 1.56487300 -2.87871100 4.38760700 H

3-hydroxyflavone with octa acid cavitand 1:2 inclusion complex

ONIOM: gridpoint 1 method: low system: model energy: 0.127851299099
ONIOM: gridpoint 2 method: high system: model energy: -803.486577426288
ONIOM: gridpoint 3 method: low system: real energy: 1.382864123961

ONIOM: extrapolated energy = -802.231564601427

Sum of electronic and thermal Free Energies= -799.049990

O-O_R 0 -3.86031500 -4.65300700 -8.34221700 L

C-C_R 0 -3.49900800 -3.31290300 -8.49980600 L

O-O_R 0 -3.44554900 -2.84004500 -9.66628800 L

C-C_R 0 -3.20988000 -2.43300900 -7.34070900 L

H-H_ 0 -2.46109200 -4.01353400 -6.05936700 L

C-C_R 0 -2.69581200 -2.96041500 -6.14316400 L

C-C_R 0 -3.39900600 -1.04356000 -7.44921700 L

H-H_ 0 -3.76944600 -0.61251200 -8.37133600 L

C-C_R 0 -2.39909600 -2.11622100 -5.06381200 L

C-C_R 0 -3.13915700 -0.19808100 -6.36577400 L

O-O_R 0 -1.73578400 -2.60914000 -3.93284700 L

C-C_R 0 -2.62634600 -0.74343000 -5.17497800 L

O-O_R 0 -3.30610500 1.18802800 -6.53279500 L

C-C_R 0 -2.42566400 -3.59119000 -3.21009100 L

H-H_ 0 -4.32745900 -2.57772100 -3.24477400 L

H-H_ 0 -2.36948000 -0.09915100 -4.34398700 L

C-C_R 0 -3.83546600 1.86738500 -5.42554200 L

C-C_R 0 -3.78701900 -3.44691700 -2.89320700 L

C-C_R 0 -1.70266200 -4.65497700 -2.66344000 L

H-H_ 0 -5.55956200 0.60181500 -5.24989000 L

H-H_ 0 -2.22486100 3.28957000 -5.37023300 L

H-H_ 0 -6.44627700 -1.63222100 -4.06228600 L

H-H_ 0 -0.64836100 -4.75461300 -2.87767000 L

C-C_R 0 -7.26035200 -1.60938000 -3.34901600 L

C-C_R 0 -3.15747100 2.97840800 -4.91716600 L

C-C_R 0 -5.04488400 1.46064300 -4.83935600 L

O-O_R 0 -11.20589600 -6.85067400 -2.04803900 L

O-O_R 0 -6.70447100 -3.86698700 -2.72998800 L

C-C_R 0 -7.54058100 -2.74444100 -2.58315500 L

C-C_R 0 -7.96125400 -0.41927400 -3.14743200 L

O-O_R 0 -7.54698100 0.73621100 -3.82897900 L

H-H_ 0 -11.70553000 0.08938700 -2.61394000 L

H-H_ 0 -11.00890300 -4.03387600 -1.53624300 L

O-O_R 0 -12.64583200 2.27976900 -4.21981400 L

C-C_R 0 -4.42951200 -4.37218800 -2.05686800 L

C-C_R 0 -2.32642500 -5.58438800 -1.82771600 L

C-C_R 0 -10.50345100 -6.65613500 -0.85884900 L

H-H_ 0 -9.72660500 1.47662400 -2.98333200 L

C-C_R 0 -9.00977600 -0.36587500 -2.20448800 L

C-C_R 0 -5.56140300 2.13865300 -3.72192900 L

C-C_R 0 -8.58926400 -2.71153000 -1.62502300 L

H-H_ 0 -8.68133900 -4.82281900 -1.47490800 L

C-C_R 0 -3.64857300 3.66756400 -3.80439500 L

C-C_3 0 -11.30570800 0.73133100 -1.79889200 L

C-C_R 0 -9.27975600 -1.50167900 -1.41961900 L

C-C_3 0 -5.83990700 -4.08552500 -1.60544700 L

C-C_3 0 -10.46574100 -4.14378500 -0.57230700 L

C-C_R 0 -3.68927100 -5.45342000 -1.53654100 L

C-C_3 0 -9.77998800 0.93036700 -2.01734600 L

H-H_ 0 -13.15766200 1.83600000 -1.52848700 L

C-C_R 0 -12.03614100 2.79004500 -3.07383300 L

C-C_3 0 -8.94384300 -3.96090700 -0.82472400 L

C-C_3 0 -6.80309000 1.65179500 -3.00741800 L

O-O_R 0 -1.61892800 -6.71708700 -1.41259400 L

O-O_R 0 -2.85925300 4.70197800 -3.26555100 L

H-H_ 0 -11.90464900 -5.52630700 0.28800900 L

C-C_3 0 -10.81850900 -5.50416200 0.05573200 L

C-C_R 0 -4.84183700 3.22543300 -3.19722800 L

O-O_R 0 -6.36138900 -5.17335200 -0.82911800 L

C-C_3 0 -12.09396000 2.05342600 -1.76368400 L

O-O_R 0 -9.60288900 -7.47551800 -0.53762900 L

H-H_ 0 -10.02239500 -1.44019600 -0.64157500 L

O-O_R 0 -11.44352800 3.89931500 -3.13670200 L

O-O_R 0 -7.64184000 2.77042900 -2.66860400 L

H-H_ 0 -0.55800600 -8.80917300 -0.39456500 L

H-H_ 0 -10.85857300 -3.35660300 0.10123800 L

H-H_ 0 -11.51258000 0.19538300 -0.85169600 L

H-H_ 0 -5.74533000 -3.14729700 -0.99562400 L

H-H_ 0 -4.91110400 6.19863000 -4.07500300 L

H-H_ 0 -6.42598100 1.14045600 -2.09045300 L

H-H_ 0 -4.15072700 -6.18797300 -0.88994700 L

C-C_R 0 -0.61045400 -8.00398000 0.32677300 L

H-H_ 0 -5.19855500 3.70939600 -2.30093000 L

C-C_R 0 -1.18930500 -6.79445500 -0.07127200 L

C-C_R 0 -8.09338800 -4.03514800 0.43635400 L

C-C_R 0 -3.51820800 5.69237300 -2.52239600 L

C-C_R 0 -6.87324400 -4.73980600 0.39435200 L

C-C_R 0 -9.09493500 1.78045900 -0.96131900 L

H-H_ 0 -10.27651900 -5.62396100 1.01885400 L

C-C_R 0 -4.56712900 6.44284900 -3.07867000 L

C-C_R 0 -8.12150100 2.72772800 -1.34841500 L

H-H_ 0 -11.70608100 2.70037000 -0.94753000 L

O-O_R 0 0.18482500 -10.66787400 1.47917100 L

O-O_R 0 -6.42370500 8.48412600 -4.29025000 L

C-C_R 0 0.57823700 -9.43873400 2.01360200 L

O-O_R 0 1.47923300 -9.43086300 2.89391200 L

C-C_R 0 -0.05551100 -8.15998200 1.60452800 L

C-C_R 0 -6.02496200 -4.75174500 1.49754400 L

C-C_R 0 -3.06898400 5.98392200 -1.23268500 L

C-C_R 0 -1.21295500 -5.71112900 0.84003700 L

C-C_R 0 -8.43170200 -3.32837400 1.60752300 L

C-C_R 0 -6.28582300 8.27461400 -2.91613700 L

C-C_R 0 -7.54133200 3.56198600 -0.38878400 L

C-C_R 0 -5.16712700 7.48383800 -2.34619200 L

C-C_R 0 -9.37558900 1.60912300 0.40622300 L

H-H_ 0 -5.04462200 -5.20447900 1.41181400 L

H-H_ 0 -9.33089300 -2.73217800 1.63198100 L

H-H_ 0 -6.79299700 4.28445200 -0.68495200 L

H-H_ 0 -10.04068500 0.81917100 0.71626400 L

H-H_ 0 -2.25510000 5.41450100 -0.80633900 L

H-H_ 0 -1.63630700 -4.76799700 0.54642500 L

O-O_R 0 -7.13218500 8.79694100 -2.14285600 L

C-C_R 0 -0.04490100 -7.06321700 2.47657900 L

C-C_R 0 -0.61570200 -5.83986400 2.11361800 L

C-C_R 0 -6.34068100 -4.01596900 2.63363100 L

C-C_R 0 -3.68518900 6.98973800 -0.47596700 L

C-C_R 0 -4.71521300 7.74902300 -1.04071400 L

C-C_R 0 -7.57606200 -3.34155400 2.72680500 L

C-C_R 0 -7.85062000 3.41026900 0.96531400 L

C-C_R 0 -8.79099100 2.44423300 1.37468800 L

H-H_ 0 -3.04543300 -4.59169900 3.20289800 L

H-H_ 0 -11.42884000 3.24721000 1.66351600 L

H-H_ 0 0.43054200 -7.14673500 3.44660800 L

H-H_ 0 -5.15314100 -1.93714600 2.42450400 L

H-H_ 0 -10.92215300 1.04597200 2.75592900 L

H-H_ 0 -5.42175900 5.63227200 0.99229400 L

O-O_R 0 -5.31428800 -3.73682500 3.54094000 L

C-C_R 0 -2.55647600 -3.63167200 3.10370000 L

O-O_R 0 -0.39344600 -4.76031800 2.99039700 L

H-H_ 0 -5.74545700 2.56079200 1.87221900 L

H-H_ 0 -5.17250800 8.53364900 -0.45040100 L

O-O_R 0 -7.13675000 4.15189600 1.91559400 L

O-O_R 0 -3.28122900 7.23860300 0.84420300 L

C-C_3 0 -11.49818300 3.11560300 2.76369900 L

C-C_3 0 -4.78962600 -2.40962200 3.37587300 L

C-C_3 0 -7.90118200 -2.57356300 3.99687000 L

H-H_ 0 -10.09494900 -2.43754300 3.75146600 L

C-C_3 0 -9.06673100 2.25411400 2.85707900 L

C-C_R 0 -4.64574200 5.38718500 1.70466300 L

H-H_ 0 -9.53127100 -3.91030100 4.56168200 L

C-C_3 0 -10.55551200 1.98555000 3.21347600 L

C-C_R 0 -8.40262900 -0.15214600 3.37478800 L

O-O_R 0 -10.70936200 5.51995800 2.78938000 L

H-H_ 0 -9.34981300 -0.47909800 2.97946200 L

C-C_3 0 -9.35597800 -2.81477700 4.48625800 L

H-H_ 0 -12.54625300 2.82056700 2.98545900 L

C-C_3 0 -6.04547500 3.42679300 2.50484300 L

C-C_R 0 -1.17028000 -3.60012900 2.88152800 L

C-C_R 0 -3.28678800 -2.42983200 3.22606100 L

C-C_R 0 -7.49592900 -1.11092500 3.85906800 L

C-C_R 0 -3.46570000 6.15415700 1.72060400 L

C-C_R 0 -8.09537700 1.22053200 3.40198700 L

C-C_R 0 -4.81716000 4.30690200 2.58759600 L

H-H_ 0 -7.27821400 -3.00666100 4.80929500 L

C-C_R 0 -11.20947800 4.41115800 3.47319700 L

H-H_ 0 -8.84409500 3.20879400 3.37914300 L

C-C_R 0 -6.19660900 -0.69144800 4.23933500 L

C-C_R 0 -6.81881100 1.62794600 3.85466200 L

O-O_R 0 -5.17134100 -1.61658000 4.51251100 L

H-H_ 0 -10.64575800 1.85587300 4.31474300 L

C-C_R 0 -5.88958000 0.67134600 4.27597800 L

C-C_R 0 -2.47312700 5.86365700 2.66081800 L

C-C_R 0 -2.60672300 -1.20622200 3.12541000 L

C-C_R 0 -0.51405100 -2.36807800 2.79157500 L

O-O_R 0 -6.43247000 2.97959700 3.81362700 L

C-C_R 0 -3.77738300 3.98832000 3.47420700 L

C-C_3 0 -9.66415100 -2.16380800 5.84717200 L

C-C_R 0 -1.21834900 -1.17249900 2.93036400 L

C-C_R 0 -2.60145900 4.76291500 3.51437000 L

H-H_ 0 -1.57727500 6.47088000 2.69954500 L

H-H_ 0 -3.15701900 -0.27492500 3.18008700 L

H-H_ 0 -4.89878700 0.98448100 4.57795600 L

H-H_ 0 0.55509100 -2.33481600 2.63883700 L

O-O_R 0 -11.41688100 4.49857900 4.71216300 L

H-H_ 0 -10.73558000 -2.32557800 6.09192900 L

H-H_ 0 -1.17200900 2.58425500 2.60378500 L

O-O_R 0 -8.99297300 -4.05771600 7.38049400 L

H-H_ 0 -3.88701800 3.15004800 4.14850000 L

H-H_ 0 -9.50817300 -1.06554600 5.78516100 L

C-C_R 0 -8.82924300 -2.73840400 6.95855300 L

C-C_R 0 -1.07443600 2.25386900 3.62993700 L

O-O_R 0 -1.58017600 4.50901700 4.44605500 L

O-O_R 0 -0.53099700 0.04508900 2.82145500 L

C-C_R 0 -1.28366600 3.15734200 4.68670000 L

C-C_R 0 -0.73911600 0.92448000 3.89373700 L

O-O_R 0 -7.97854800 -2.01115600 7.53563300 L

C-C_R 0 -1.09501900 2.73229300 6.00649200 L

C-C_R 0 -0.54871000 0.50770300 5.22252000 L

C-C_R 0 -0.72870100 1.40472500 6.28786300 L

H-H_ 0 -0.24969900 -0.51479100 5.41950700 L

H-H_ 0 -1.19652200 3.45851300 6.80219900 L

C-C_R 0 -0.48215300 0.95718500 7.68067900 L

O-O_R 0 0.32341600 0.01241800 7.89490600 L

O-O_R 0 -1.13217800 1.53660600 8.77264300 L

H-H_ 0 -4.04857300 -4.91256900 -7.38254700 L

H-H_ 0 -11.93635900 -6.16251700 -2.18563800 L

H-H_ 0 -13.13080700 1.40742200 -4.04696100 L

H-H_ 0 -0.65754900 -10.61808600 0.92121900 L

H-H_ 0 -5.62028500 8.18783800 -4.82895600 L

H-H_ 0 -10.54705600 5.33730000 1.80634200 L

H-H_ 0 -9.73246000 -4.53712400 6.88109700 L

H-H_ 0 -1.88164400 2.16430400 8.51197500 L

O-O_R 0 3.71800900 9.12844100 2.08390200 L

C-C_R 0 2.67161800 8.89586700 1.18823000 L

O-O_R 0 2.28974000 9.84483800 0.45292700 L

C-C_R 0 2.02981400 7.56533600 1.05520200 L

H-H_ 0 2.36328300 6.96102900 3.11268300 L

C-C_R 0 1.97149300 6.67714400 2.14471900 L

C-C_R 0 1.44600000 7.18642000 -0.16736200 L

H-H_ 0 1.48249100 7.85070100 -1.02212600 L

C-C_R 0 1.35720900 5.42378200 2.00762900 L

C-C_R 0 0.85771200 5.92576500 -0.31377200 L

O-O_R 0 1.22934700 4.56161200 3.10335500 L

C-C_R 0 0.78757500 5.06071900 0.78649100 L

O-O_R 0 0.33848900 5.54068500 -1.55717100 L

C-C_R 0 2.41569700 4.06387800 3.67738800 L

H-H_ 0 3.71393900 4.36520300 1.97125000 L

H-H_ 0 0.29894700 4.10160000 0.69062600 L

C-C_R 0 1.00494600 4.44541400 -2.12817700 L

C-C_R 0 3.64048600 3.98785200 2.98053900 L

C-C_R 0 2.33463800 3.52272000 4.96307700 L

H-H_ 0 2.96216600 5.31045800 -1.95666900 L

H-H_ 0 -0.80826400 3.33122600 -2.42799900 L

H-H_ 0 4.92756700 5.08769000 0.02338500 L

H-H_ 0 1.40941200 3.59057300 5.51323000 L

C-C_R 0 5.89350900 4.66726900 -0.22544300 L

C-C_R 0 0.26404300 3.34218700 -2.55817900 L

C-C_R 0 2.40148000 4.44744700 -2.28971500 L

O-O_R 0 11.45954100 5.89812900 3.29269900 L

O-O_R 0 6.37293900 4.50580900 2.12698500 L

C-C_R 0 6.78946200 4.33787700 0.79392900 L

C-C_R 0 6.18172700 4.35767200 -1.55449900 L

O-O_R 0 5.17117200 4.49755800 -2.51504300 L

H-H_ 0 9.41559100 4.86155200 -3.46867100 L

H-H_ 0 10.59621800 4.74993500 0.80565600 L

O-O_R 0 8.87949300 6.00346500 -6.04849000 L

C-C_R 0 4.77023300 3.39995700 3.57508000 L

C-C_R 0 3.42286800 2.86957600 5.54124400 L

C-C_R 0 11.23605800 4.53401100 3.47880900 L

H-H_ 0 7.11690500 4.19345600 -3.96384700 L

C-C_R 0 7.42185100 3.78265400 -1.89865500 L

C-C_R 0 3.06125700 3.33440000 -2.84216400 L

C-C_R 0 8.04225600 3.75374500 0.47282200 L

H-H_ 0 8.83805500 4.14412100 2.39963700 L

C-C_R 0 0.90046900 2.23142700 -3.11662400 L

C-C_3 0 9.18424500 3.81717800 -3.77212200 L

C-C_R 0 8.32365800 3.44654500 -0.87309700 L

C-C_3 0 6.05406400 3.27040300 2.78700500 L

C-C_3 0 10.51828400 3.71222600 1.19728600 L

C-C_R 0 4.64528200 2.82680600 4.85456500 L

C-C_3 0 7.72341800 3.48561700 -3.35904200 L

H-H_ 0 10.52010000 3.83695000 -5.48616000 L

C-C_R 0 8.63732400 4.63097000 -6.10337500 L

C-C_3 0 9.03899100 3.42590900 1.57611100 L

C-C_3 0 4.56890300 3.24683500 -2.88735500 L

O-O_R 0 3.28976400 2.35445100 6.83957900 L

O-O_R 0 0.13797600 1.09071000 -3.40225200 L

H-H_ 0 12.53239600 3.68246500 2.01252300 L

C-C_3 0 11.49241100 3.54242200 2.37712100 L

C-C_R 0 2.30027400 2.22525900 -3.24225800 L

O-O_R 0 7.14300100 2.92194900 3.65390800 L

C-C_3 0 9.44231600 3.66164200 -5.28192000 L

O-O_R 0 10.83112500 4.12626200 4.59923800 L

H-H_ 0 9.23606100 2.92979400 -1.12211700 L

O-O_R 0 7.74006600 4.19692300 -6.87287700 L

O-O_R 0 5.00499500 2.88829300 -4.20905000 L

H-H_ 0 4.93274500 1.72804100 8.76190600 L

H-H_ 0 10.86299200 3.04515100 0.38280200 L

H-H_ 0 9.91109600 3.17261300 -3.23954700 L

H-H_ 0 5.84999400 2.46255300 2.03944200 L

H-H_ 0 0.08975100 2.21236200 -5.81074000 L

H-H_ 0 4.83017800 2.44218300 -2.15506300 L

H-H_ 0 5.49002200 2.34569600 5.32835200 L

C-C_R 0 4.67162100 0.87522000 8.14888500 L

H-H_ 0 2.79802500 1.33486500 -3.60254200 L

C-C_R 0 3.83016200 1.07590300 7.04937400 L

C-C_R 0 8.78282000 2.01991400 2.09903300 L

C-C_R 0 0.43521200 0.46319400 -4.61846700 L

C-C_R 0 7.88816300 1.84252300 3.17313500 L

C-C_R 0 7.26715000 2.07706700 -3.70514100 L

H-H_ 0 11.42329000 2.50668300 2.77455400 L

C-C_R 0 0.30771900 1.15325300 -5.83397400 L

C-C_R 0 5.96463800 1.86086100 -4.21229300 L

H-H_ 0 9.21681600 2.62019900 -5.59653900 L

O-O_R 0 6.91887400 0.35236700 10.08388400 L

O-O_R 0 -0.53254800 2.31261000 -8.48538600 L

C-C_R 0 6.02924800 -0.62847300 9.63923300 L

O-O_R 0 6.01648100 -1.73918400 10.23366900 L

C-C_R 0 5.13378400 -0.41126100 8.47593900 L

C-C_R 0 7.58279000 0.56420500 3.63539800 L

C-C_R 0 0.72614000 -0.90171800 -4.62441000 L

C-C_R 0 3.42072600 -0.02730500 6.27777900 L

C-C_R 0 9.34142100 0.88314500 1.48368600 L

C-C_R 0 0.27803400 1.18293000 -8.35103500 L

C-C_R 0 5.57132100 0.57669000 -4.59768200 L

C-C_R 0 0.43859900 0.47332900 -7.05764800 L

C-C_R 0 8.10782200 0.97084000 -3.49337100 L

H-H_ 0 6.83546900 0.43818600 4.40853300 L

H-H_ 0 9.97375800 1.00179500 0.61807500 L

H-H_ 0 4.56981400 0.41443800 -4.97348300 L

H-H_ 0 9.06415200 1.11631400 -3.01767300 L

H-H_ 0 0.78881400 -1.43195000 -3.68202500 L

H-H_ 0 2.71875400 0.11032900 5.46459300 L

O-O_R 0 0.86558500 0.74344100 -9.37507000 L

C-C_R 0 4.68392300 -1.50789000 7.72173400 L

C-C_R 0 3.84012100 -1.32156900 6.62214700 L

C-C_R 0 8.12360100 -0.55767400 3.01403900 L

C-C_R 0 0.90567900 -1.58754200 -5.83975100 L

C-C_R 0 0.73545100 -0.90093000 -7.04610100 L

C-C_R 0 9.05448000 -0.40982700 1.96300500 L

C-C_R 0 6.42434200 -0.51508500 -4.41631400 L

C-C_R 0 7.71829400 -0.32145500 -3.88649900 L

H-H_ 0 5.67078600 -1.84627700 4.83719300 L

H-H_ 0 9.81361600 0.09930200 -5.65759400 L

H-H_ 0 4.98226400 -2.51481800 7.98721700 L

H-H_ 0 6.40210600 -1.46957000 1.59088600 L

H-H_ 0 10.56691400 -0.49677700 -3.33540100 L

H-H_ 0 3.49467000 -1.82726500 -5.20393300 L

O-O_R 0 7.58081900 -1.81245700 3.31705300 L

C-C_R 0 4.98601200 -2.38772700 4.19951000 L

O-O_R 0 3.28109400 -2.44311900 5.99610600 L

H-H_ 0 5.29991200 -1.82066600 -2.67071500 L

H-H_ 0 0.83398900 -1.44668300 -7.97654500 L

O-O_R 0 5.94244300 -1.80533600 -4.68423400 L

O-O_R 0 1.16097600 -2.97006200 -5.87685300 L

C-C_3 0 10.36715200 -0.83971600 -5.44865300 L

C-C_3 0 6.68730100 -2.29030100 2.29867100 L

C-C_3 0 9.66149400 -1.65004900 1.32972700 L

H-H_ 0 11.38841400 -0.71963800 0.30678600 L

C-C_3 0 8.62770200 -1.51810300 -3.66533400 L

C-C_R 0 3.29758900 -2.75665900 -4.68646600 L

H-H_ 0 11.69151100 -1.19256300 1.98968900 L

C-C_3 0 10.12739300 -1.27310700 -3.99179200 L

C-C_R 0 9.07751600 -1.58221800 -1.15244900 L

O-O_R 0 8.90780800 -1.74689900 -7.30636100 L

H-H_ 0 9.81138900 -0.80330400 -1.27873400 L

C-C_3 0 11.18423200 -1.51258700 1.05334200 L

H-H_ 0 11.44628300 -0.61228200 -5.58422600 L

C-C_3 0 5.52870300 -2.52237400 -3.50932600 L

C-C_R 0 3.70530700 -2.72510000 4.68474800 L

C-C_R 0 5.37360800 -2.74625500 2.89245100 L

C-C_R 0 8.82839700 -2.09044800 0.13524500 L

C-C_R 0 2.10171300 -3.45015300 -4.94917200 L

C-C_R 0 8.37853500 -2.06922100 -2.27205300 L

C-C_R 0 4.24102400 -3.26889900 -3.78211000 L

H-H_ 0 9.60702600 -2.46015400 2.08871200 L

C-C_R 0 9.98110500 -1.91326700 -6.43028700 L

H-H_ 0 8.33314600 -2.31117700 -4.38508200 L

C-C_R 0 7.77260100 -3.01574400 0.31380400 L

C-C_R 0 7.35876400 -3.02508600 -2.07731400 L

O-O_R 0 7.32228200 -3.37261600 1.59700300 L

H-H_ 0 10.69677700 -2.20848000 -3.79515100 L

C-C_R 0 7.08333100 -3.50735500 -0.79623200 L

C-C_R 0 1.86845000 -4.67897700 -4.32434500 L

C-C_R 0 4.46112500 -3.43454200 2.07715400 L

C-C_R 0 2.83490700 -3.44871400 3.86468600 L

O-O_R 0 6.56226300 -3.45222700 -3.14896600 L

C-C_R 0 3.94225000 -4.45745000 -3.09377500 L

C-C_3 0 11.83548100 -2.81281800 0.54776200 L

C-C_R 0 3.19788300 -3.80569100 2.56387900 L

C-C_R 0 2.75030100 -5.16217300 -3.35276500 L

H-H_ 0 0.97274000 -5.23810500 -4.56183500 L

H-H_ 0 4.71782600 -3.65458800 1.05060300 L

H-H_ 0 6.28079800 -4.21871400 -0.65562700 L

H-H_ 0 1.85923600 -3.72651300 4.24193500 L

O-O_R 0 10.63504000 -2.98896800 -6.46091000 L

H-H_ 0 12.90221300 -2.61512700 0.30887000 L

H-H_ 0 1.77538800 -4.56945800 -0.84380200 L

O-O_R 0 12.48631700 -3.84521700 2.76117900 L

H-H_ 0 4.64283900 -4.85218400 -2.37062200 L

H-H_ 0 11.34605800 -3.13715700 -0.39571700 L

C-C_R 0 11.77185200 -3.91854400 1.56553100 L

C-C_R 0 2.35344800 -5.39637700 -0.46215400 L

O-O_R 0 2.45092300 -6.37489900 -2.70254900 L

O-O_R 0 2.23819000 -4.41245600 1.73647600 L

C-C_R 0 2.76026700 -6.41967500 -1.33194000 L

C-C_R 0 2.70379400 -5.42849100 0.88829500 L

O-O_R 0 11.07688500 -4.94143600 1.32824600 L

C-C_R 0 3.45510000 -7.52091700 -0.82159100 L

C-C_R 0 3.42964700 -6.52182400 1.38808300 L

C-C_R 0 3.81802500 -7.57252100 0.53731700 L

H-H_ 0 3.67583800 -6.55501000 2.44231700 L

H-H_ 0 3.70751000 -8.32906300 -1.49509500 L

C-C_R 0 4.57536900 -8.72583000 1.08476900 L

O-O_R 0 4.47095800 -9.01258300 2.30696600 L

O-O_R 0 5.44132600 -9.48807300 0.29715600 L

H-H_ 0 4.08388900 8.28576600 2.50811100 L

H-H_ 0 11.82651600 6.11038800 2.37272900 L

H-H_ 0 9.65019100 6.23843600 -5.43458900 L

H-H_ 0 6.99619900 1.14382200 9.45829800 L

H-H_ 0 -1.10448900 2.50284700 -7.67344900 L

H-H_ 0 8.42880600 -0.86327000 -7.18053800 L

H-H_ 0 13.04562100 -3.00343700 2.82933700 L

H-H_ 0 5.61804800 -9.09006100 -0.61587600 L

C-C_R 0 -1.68152700 1.02786700 -0.20630900 H

C-C_R 0 -2.32291000 2.26771600 -0.13415100 H

C-C_R 0 -3.70396200 2.30205500 -0.08469800 H

C-C_R 0 -4.46051700 1.11751600 -0.07825100 H

C-C_R 0 -3.82491500 -0.10192700 -0.16571100 H

C-C_R 0 -2.42362900 -0.15684200 -0.24749900 H

H-H_ 0 -1.73384100 3.17417500 -0.11189700 H

H-H_ 0 -4.20627000 3.26081000 -0.05184000 H

H-H_ 0 -5.53958200 1.16042700 0.00531600 H

H-H_ 0 -4.37333100 -1.03669800 -0.17147200 H

C-C_R 0 -1.71518800 -1.41596900 -0.35417500 H

C-C_R 0 -0.26507600 -1.32475900 -0.43996900 H

C-C_R 0 0.40010400 -0.13938500 -0.31409300 H

C-C_R 0 1.84697400 0.07306200 -0.21413200 H

C-C_R 0 2.34136700 1.35742100 0.07417500 H

C-C_R 0 2.77172800 -0.97768900 -0.37241300 H

C-C_R 0 3.70497200 1.58324500 0.19468300 H

C-C_R 0 4.13567200 -0.73836600 -0.24364800 H

H-H_ 0 2.42613000 -1.97572700 -0.59270900 H

C-C_R 0 4.61411400 0.53878100 0.03649200 H

H-H_ 0 4.05639900 2.58789900 0.39012300 H

H-H_ 0 4.82886500 -1.56294400 -0.36258100 H

H-H_ 0 5.68091100 0.71989000 0.12343800 H

O-O_R 0 -2.24972500 -2.52925900 -0.34950400 H

O-O_R 0 0.36158100 -2.51354000 -0.56018300 H

H-H_ 0 -0.35682000 -3.17489100 -0.55305900 H

O-O_R 0 -0.32186400 1.02569500 -0.22124100 H

H-H_ 0 1.65226100 2.18028000 0.19640800 H

4’-(N,N-Diethylamino)-3-hydroxyflavone with octa acid cavitand 1:2 inclusion complex

ONIOM: gridpoint 1 method: low system: model energy: 0.173864351259

ONIOM: gridpoint 2 method: high system: model energy: -1016.127453412282

ONIOM: gridpoint 3 method: low system: real energy: 1.435541220900

ONIOM: extrapolated energy = -1014.865776542641

Sum of electronic and thermal Free Energies= -1011.560140

```
O-O_R 0 -3.04604900 1.11049300 8.55823400 L
C-C_R 0 -2.61674200 -0.16770400 8.19320600 L
O-O_R 0 -2.24041900 -0.95415800 9.10227900 L
C-C_R 0 -2.62851000 -0.63145700 6.78341600 L
H-H_ 0 -2.33399300 1.33011100 5.91851600 L
C-C_R 0 -2.48929100 0.27744600 5.72232400 L
C-C_R 0 -2.73602000 -2.00186300 6.49502000 L
H-H_ 0 -2.81920400 -2.72371900 7.29827500 L
C-C_R 0 -2.48320300 -0.17034000 4.39064200 L
C-C_R 0 -2.75024200 -2.45926400 5.17327100 L
O-O_R 0 -2.27498700 0.72762100 3.33342300 L
C-C_R 0 -2.60044900 -1.53685100 4.12013300 L
O-O_R 0 -2.78606200 -3.84581500 4.94749300 L
C-C_R 0 -3.17370700 1.80343000 3.32168300 L
H-H_ 0 -4.92629500 0.55995900 3.21378200 L
H-H_ 0 -2.54926900 -1.88067200 3.09453800 L
C-C_R 0 -3.57922100 -4.30479400 3.88168200 L
C-C_R 0 -4.55653200 1.57734500 3.26015800 L
C-C_R 0 -2.68571800 3.11399500 3.32301900 L
H-H_ 0 -5.19397300 -2.89588400 4.16074500 L
H-H_ 0 -2.18269200 -5.86869200 3.39567200 L
H-H_ 0 -6.71966400 -1.00381200 4.36670400 L
H-H_ 0 -1.61823400 3.28793300 3.37652100 L
C-C_R 0 -7.65866200 -0.87166300 3.84513400 L
C-C_R 0 -3.13998500 -5.42060800 3.16031000 L
C-C_R 0 -4.83766500 -3.74090300 3.58747200 L
O-O_R 0 -12.19140700 3.47358800 5.84862300 L
O-O_R 0 -7.39869800 1.50689200 4.12061400 L
C-C_R 0 -8.17211300 0.41702800 3.67572800 L
C-C_R 0 -8.29804500 -1.98104100 3.28228000 L
```

O-O_R 0 -7.68408200 -3.24334500 3.39197000 L

H-H_ 0 -12.04675000 -2.83564900 2.88427300 L

H-H_ 0 -11.87737200 1.28499300 4.01651800 L

O-O_R 0 -12.49630100 -5.63034200 3.37995400 L

C-C_R 0 -5.45743600 2.65075600 3.22325200 L

C-C_R 0 -3.56674500 4.20137200 3.27734300 L

C-C_R 0 -11.81957100 3.97818800 4.60261800 L

H-H_ 0 -9.88342600 -3.88992500 2.44539900 L

C-C_R 0 -9.49311700 -1.80558700 2.54220800 L

C-C_R 0 -5.64572000 -4.28282100 2.57472000 L

C-C_R 0 -9.40994800 0.60045700 3.00656900 L

H-H_ 0 -9.73467400 2.48995100 3.90084200 L

C-C_R 0 -3.89979800 -5.92852600 2.10368700 L

C-C_3 0 -11.68814600 -2.97780200 1.84151300 L

C-C_R 0 -10.01579300 -0.50806300 2.39041100 L

C-C_3 0 -6.92949200 2.35555900 3.06019200 L

C-C_3 0 -11.62273900 1.92282000 3.14206000 L

C-C_R 0 -4.95289900 3.96701400 3.23233900 L

C-C_3 0 -10.13412200 -2.99270600 1.84070000 L

H-H_ 0 -13.41174900 -4.14696200 1.22087400 L

C-C_R 0 -11.98684600 -5.47691000 2.09065500 L

C-C_3 0 -10.07824200 1.96988100 2.98055300 L

C-C_3 0 -6.96222000 -3.63132100 2.21307500 L

O-O_R 0 -3.04937500 5.49877500 3.42485800 L

O-O_R 0 -3.41019500 -7.01519600 1.35862200 L

H-H_ 0 -13.36406900 3.20641800 3.34795900 L

C-C_3 0 -12.25844600 3.31264900 3.32701600 L

C-C_R 0 -5.15723900 -5.36852900 1.82722900 L

O-O_R 0 -7.70346900 3.56581000 3.05822700 L

C-C_3 0 -12.30768900 -4.26278500 1.26280100 L

O-O_R 0 -11.12022000 5.02392800 4.54845900 L

H-H_ 0 -10.89321500 -0.35569900 1.78470900 L

O-O_R 0 -11.26116200 -6.38468400 1.60596800 L

O-O_R 0 -7.76965700 -4.53893600 1.44702200 L

H-H_ 0 -3.81206200 7.88437700 4.14840400 L

H-H_ 0 -12.10226500 1.45415400 2.25956400 L

H-H_ 0 -12.08748900 -2.12688300 1.25588600 L

H-H_ 0 -6.99307400 1.82276800 2.07423000 L

H-H_ 0 -4.70263100 -8.61007200 -0.28184400 L

H-H_ 0 -6.68757100 -2.73187200 1.62069200 L

H-H_ 0 -5.62480800 4.81474300 3.21783900 L

C-C_R 0 -3.86949900 7.74019500 3.07728000 L

H-H_ 0 -5.74309600 -5.78441900 1.01874100 L

C-C_R 0 -3.55156600 6.48242400 2.55545200 L

C-C_R 0 -9.61247500 2.77559900 1.77794500 L

C-C_R 0 -3.51509700 -6.84861600 -0.03151100 L

C-C_R 0 -8.52664000 3.65977100 1.93346800 L

C-C_R 0 -9.50331700 -3.14531200 0.46512500 L

H-H_ 0 -12.00940000 3.95756200 2.45652000 L

C-C_R 0 -4.22765400 -7.78244300 -0.79192900 L

C-C_R 0 -8.35420600 -3.94519500 0.32311900 L

H-H_ 0 -11.95607300 -4.41134100 0.21890400 L

O-O_R 0 -5.12618400 10.30136600 4.03757800 L

O-O_R 0 -5.29066700 -9.91305900 -2.63747100 L

C-C_R 0 -4.54175800 10.14479600 2.77852100 L

O-O_R 0 -4.32562700 11.17512100 2.08648600 L

C-C_R 0 -4.20339200 8.80893300 2.22722700 L

C-C_R 0 -8.08633600 4.42984800 0.86326800 L

C-C_R 0 -2.91142700 -5.75476500 -0.66606900 L

C-C_R 0 -3.54718500 6.29646100 1.16331100 L

C-C_R 0 -10.17747700 2.60625400 0.49850400 L

C-C_R 0 -5.16525100 -8.56806600 -2.99287500 L

C-C_R 0 -7.71294700 -4.04876400 -0.91389800 L

C-C_R 0 -4.37931200 -7.60670200 -2.18077400 L

C-C_R 0 -9.97227900 -2.42521400 -0.64813300 L

H-H_ 0 -7.20522000 5.04917000 0.97904400 L

H-H_ 0 -10.94268400 1.85993500 0.34998500 L

H-H_ 0 -6.80946500 -4.63719800 -0.99601800 L

H-H_ 0 -10.81035200 -1.75608000 -0.53583200 L

H-H_ 0 -2.33988200 -5.04802000 -0.08364800 L

H-H_ 0 -3.23690900 5.34843800 0.76536000 L

O-O_R 0 -5.72341900 -8.17366300 -4.05123600 L

C-C_R 0 -4.14478600 8.61772500 0.83556900 L

C-C_R 0 -3.82500500 7.36866400 0.29308900 L

C-C_R 0 -8.64575700 4.26137900 -0.39982500 L

C-C_R 0 -3.06116200 -5.55858400 -2.03925600 L

C-C_R 0 -3.79789100 -6.48230400 -2.79540900 L

C-C_R 0 -9.70896700 3.35751800 -0.59841300 L

C-C_R 0 -8.17398600 -3.32320300 -2.01503400 L

C-C_R 0 -9.35176100 -2.55291400 -1.90486200 L

H-H_ 0 -6.03952000 6.19507900 -1.07403000 L

H-H_ 0 -11.71136000 -3.89411200 -2.44542800 L

H-H_ 0 -4.34219000 9.44344200 0.16270600 L

H-H_ 0 -6.92759500 2.93634900 -1.44116400 L

H-H_ 0 -11.95202700 -1.39650300 -2.43940900 L

H-H_ 0 -5.03025200 -3.90716100 -2.31863300 L

O-O_R 0 -7.96464500 4.78950000 -1.49733800 L

C-C_R 0 -5.32272300 5.61370600 -1.63856300 L

O-O_R 0 -3.62619200 7.27687400 -1.09863500 L

H-H_ 0 -6.63181800 -1.47405700 -2.40034900 L

H-H_ 0 -3.88960700 -6.33429200 -3.86454700 L

O-O_R 0 -7.39357400 -3.28370400 -3.18359100 L

O-O_R 0 -2.39246000 -4.48961200 -2.65105900 L

C-C_3 0 -11.98160300 -3.33583400 -3.36623900 L

C-C_3 0 -7.08209000 3.83175600 -2.09934900 L

C-C_3 0 -10.21891400 3.12450800 -2.01236500 L

H-H_ 0 -12.16187600 2.18720100 -1.51639100 L

C-C_3 0 -9.89311800 -1.83534900 -3.12743000 L

C-C_R 0 -4.53235100 -3.32187900 -3.07581700 L

H-H_ 0 -12.19589600 3.95632400 -1.61305200 L

C-C_3 0 -11.43833200 -1.89839400 -3.28298300 L

C-C_R 0 -9.95920100 0.64267700 -2.53187200 L

O-O_R 0 -10.62382700 -5.15025800 -4.49242500 L

H-H_ 0 -10.83916800 0.45463000 -1.93953500 L

C-C_3 0 -11.76870100 3.04829700 -2.09209200 L

H-H_ 0 -13.09150500 -3.29985900 -3.40450400 L

C-C_3 0 -6.68869600 -2.04102500 -3.35968300 L

C-C_R 0 -3.99892100 6.06795600 -1.70379300 L

C-C_R 0 -5.70107000 4.41557600 -2.27382900 L

C-C_R 0 -9.46998500 1.95658400 -2.64514200 L

C-C_R 0 -3.17047800 -3.55206700 -3.35590600 L

C-C_R 0 -9.32378600 -0.42913100 -3.18221300 L

C-C_R 0 -5.24792900 -2.31283100 -3.74103900 L

H-H_ 0 -9.97364300 4.02979400 -2.60798700 L

C-C_R 0 -11.48754300 -4.05866500 -4.59041900 L

H-H_ 0 -9.52063800 -2.36600100 -4.02971800 L

C-C_R 0 -8.25192500 2.17475600 -3.34080800 L

C-C_R 0 -8.10655700 -0.19565900 -3.85798600 L

O-O_R 0 -7.62974600 3.43654800 -3.36621800 L

H-H_ 0 -11.73206800 -1.34723100 -4.20373800 L

C-C_R 0 -7.59987700 1.10207100 -3.95695700 L

C-C_R 0 -2.52209000 -2.73902100 -4.28948300 L

C-C_R 0 -4.72962800 3.67703900 -2.96549800 L

C-C_R 0 -3.05393200 5.34533400 -2.43959500 L

O-O_R 0 -7.35034200 -1.26240100 -4.37161600 L

C-C_R 0 -4.57021800 -1.50096900 -4.66801300 L

C-C_3 0 -12.30549300 2.94149300 -3.53124800 L

C-C_R 0 -3.40423700 4.14496300 -3.06833700 L

C-C_R 0 -3.20134000 -1.70320100 -4.93562200 L

H-H_ 0 -1.47066800 -2.89722000 -4.49598700 L

H-H_ 0 -4.99571700 2.71485700 -3.38259300 L

H-H_ 0 -6.66029800 1.26876900 -4.46806200 L

H-H_ 0 -2.03228700 5.69990900 -2.49255300 L

O-O_R 0 -11.85444600 -3.66895400 -5.73024700 L

H-H_ 0 -13.40984100 2.82583100 -3.49860000 L

H-H_ 0 -1.97124700 0.78738700 -3.91512300 L

O-O_R 0 -12.54424400 5.39858200 -4.07411500 L

H-H_ 0 -5.09780600 -0.70793600 -5.18041900 L

H-H_ 0 -11.89830600 2.03042100 -4.01971000 L

C-C_R 0 -11.97754900 4.15573800 -4.35629300 L

C-C_R 0 -2.46701900 1.23022400 -4.77045000 L

O-O_R 0 -2.50513600 -0.93420900 -5.88457800 L

O-O_R 0 -2.38729100 3.38022700 -3.66969700 L

C-C_R 0 -2.80765400 0.43335600 -5.87290200 L

C-C_R 0 -2.77091800 2.59430300 -4.76698800 L

O-O_R 0 -11.19145500 4.04806800 -5.33407300 L

C-C_R 0 -3.41587700 1.01378700 -6.99106000 L

C-C_R 0 -3.40167100 3.16831900 -5.88365900 L

C-C_R 0 -3.74169100 2.38145300 -6.99767000 L

H-H_ 0 -3.62288000 4.22873000 -5.87819000 L

H-H_ 0 -3.62573000 0.38893600 -7.84923800 L

C-C_R 0 -4.40410400 3.00441300 -8.17002600 L

O-O_R 0 -4.22684700 4.22952200 -8.40409900 L

O-O_R 0 -5.25343300 2.28327600 -9.01246900 L

H-H_ 0 -3.50108100 1.61627000 7.80942500 L

H-H_ 0 -12.78939900 2.65922000 5.77601900 L

H-H_ 0 -13.10230900 -4.86575500 3.65242800 L

H-H_ 0 -5.42510300 9.43069600 4.45748200 L

H-H_ 0 -4.68050800 -10.19160800 -1.88009600 L

H-H_ 0 -10.36382400 -5.36347300 -3.53691400 L

H-H_ 0 -13.19411500 5.36283300 -3.29794500 L

H-H_ 0 -5.48522600 1.36235700 -8.66333100 L

O-O_R 0 2.85903200 -5.47940900 -6.24736900 L

C-C_R 0 1.78235800 -5.56475800 -5.36151700 L

O-O_R 0 1.03020700 -6.57323900 -5.42707900 L

C-C_R 0 1.52417300 -4.52744500 -4.33348000 L

H-H_ 0 2.35397700 -2.88606300 -5.47946700 L

C-C_R 0 1.89839800 -3.18795700 -4.54555000 L

C-C_R 0 0.87540200 -4.87413200 -3.13527300 L

H-H_ 0 0.58602800 -5.90040300 -2.94448500 L

C-C_R 0 1.63599500 -2.21019200 -3.56990700 L

C-C_R 0 0.63460300 -3.90913600 -2.15293700 L

O-O_R 0 1.95509800 -0.85888000 -3.77847000 L

C-C_R 0 0.97265300 -2.57201800 -2.39691300 L

O-O_R 0 0.04112600 -4.28933400 -0.94048200 L

C-C_R 0 3.26207500 -0.57975200 -4.21995900 L

H-H_ 0 4.24124600 -2.09720500 -3.04240300 L

H-H_ 0 0.73017800 -1.81679600 -1.66685000 L

C-C_R 0 0.79119700 -4.01546300 0.21934200 L

C-C_R 0 4.38026600 -1.29711700 -3.75619500 L

C-C_R 0 3.45916000 0.52963800 -5.04417000 L

H-H_ 0 2.73769900 -4.32454100 -0.67288500 L

H-H_ 0 -0.97213900 -3.73969000 1.42825900 L

H-H_ 0 4.86662500 -4.15363000 -1.96649400 L

H-H_ 0 2.60294400 1.11102400 -5.36343100 L

C-C_R 0 5.85782000 -4.15659100 -1.53288800 L

C-C_R 0 0.10871800 -3.75254400 1.41394500 L

C-C_R 0 2.20032000 -4.08430500 0.23445800 L

O-O_R 0 11.23225000 -5.39436500 -5.22500000 L

O-O_R 0 6.68849700 -3.07527300 -3.53053200 L

C-C_R 0 6.93768200 -3.67307200 -2.27762100 L

C-C_R 0 6.02728100 -4.56958900 -0.20639800 L

O-O_R 0 4.89293300 -4.95925300 0.52673700 L

H-H_ 0 8.92755500 -6.53797400 1.41483200 L

H-H_ 0 10.39592600 -5.22633200 -2.47878600 L

O-O_R 0 7.89433600 -8.53580600 3.20267800 L

C-C_R 0 5.67891300 -0.95124800 -4.16431000 L

C-C_R 0 4.74203600 0.92225100 -5.43902900 L

C-C_R 0 11.45688100 -4.09742300 -4.76365900 L

H-H_ 0 6.71550700 -5.70778700 2.05228000 L

C-C_R 0 7.31290200 -4.53448600 0.38865600 L

C-C_R 0 2.91479400 -3.88224400 1.42486900 L

C-C_R 0 8.23721500 -3.68859500 -1.71753200 L

H-H_ 0 9.14823700 -3.45179000 -3.61707800 L

C-C_R 0 0.80963700 -3.46455800 2.58820100 L

C-C_3 0 8.82325000 -5.69821700 2.13604100 L

C-C_R 0 8.39724900 -4.06185500 -0.37159800 L

C-C_3 0 6.86119400 -1.64606800 -3.52360600 L

C-C_3 0 10.68600100 -4.16006100 -2.35536400 L

C-C_R 0 5.85312900 0.16150800 -5.01120400 L

C-C_3 0 7.49553100 -4.94490000 1.84439200 L

H-H_ 0 9.90969200 -6.70167200 3.72826200 L

C-C_R 0 7.86357500 -7.29771400 3.84423900 L

C-C_3 0 9.42989400 -3.26848200 -2.55783900 L

C-C_3 0 4.42324200 -3.95703200 1.44131800 L

O-O_R 0 4.84187900 2.08196900 -6.23442400 L

O-O_R 0 0.09127400 -3.21672400 3.76832100 L

H-H_ 0 12.71498200 -4.45300700 -3.07643600 L

C-C_3 0 11.83047700 -3.83101400 -3.33113200 L

C-C_R 0 2.20965900 -3.54053300 2.59014600 L

O-O_R 0 8.09518400 -1.30555000 -4.18319800 L

C-C_3 0 8.90721200 -6.24954700 3.57100900 L

O-O_R 0 11.36395000 -3.13495700 -5.57028900 L

H-H_ 0 9.36774300 -3.96996200 0.08642500 L

O-O_R 0 6.95405500 -7.06423800 4.68319100 L

O-O_R 0 4.87471000 -4.29246700 2.76108400 L

H-H_ 0 6.67075100 1.45837500 -8.02572600 L

H-H_ 0 11.08733400 -4.06238800 -1.32714500 L

H-H_ 0 9.70324200 -5.04351100 1.98191200 L

H-H_ 0 6.85003900 -1.26804700 -2.48128200 L

H-H_ 0 0.44709000 -3.45618200 6.33940000 L

H-H_ 0 4.77323100 -2.94498600 1.12952500 L

H-H_ 0 6.85099600 0.46111200 -5.29464600 L

C-C_R 0 6.96944300 2.27742200 -7.38451600 L

H-H_ 0 2.73910000 -3.36269100 3.51503700 L

C-C_R 0 6.10306600 2.67704000 -6.35935800 L

C-C_R 0 9.68479200 -1.78172200 -2.38813100 L

C-C_R 0 0.60503500 -2.21405300 4.60921700 L

C-C_R 0 9.09655700 -0.87776200 -3.29567900 L

C-C_R 0 7.24222100 -3.74260700 2.74343600 L

H-H_ 0 12.13353600 -2.76857600 -3.20906300 L

C-C_R 0 0.67839300 -2.46107700 5.98387600 L

C-C_R 0 5.93290700 -3.49077400 3.19664200 L

H-H_ 0 8.80819100 -5.41599400 4.29964700 L

O-O_R 0 9.19126200 1.21560200 -9.11567700 L

O-O_R 0 0.38473900 -2.68255500 8.97147700 L

C-C_R 0 9.10731200 2.54106000 -8.68280000 L

O-O_R 0 9.87040900 3.39327800 -9.21052300 L

C-C_R 0 8.17974600 2.95963500 -7.60319300 L

C-C_R 0 9.39565300 0.48248500 -3.21629300 L

C-C_R 0 0.90698700 -0.93100800 4.12014100 L

C-C_R 0 6.43246600 3.77692900 -5.55918500 L

C-C_R 0 10.44827500 -1.27891900 -1.31657500 L

C-C_R 0 1.14757900 -1.69870900 8.33785200 L

C-C_R 0 5.65216300 -2.35289200 3.95576300 L

C-C_R 0 1.05836000 -1.44429100 6.87835200 L

C-C_R 0 8.25879800 -2.82153200 3.05878300 L

H-H_ 0 8.92631700 1.17167200 -3.90588000 L

H-H_ 0 10.80051800 -1.95635500 -0.55522500 L

H-H_ 0 4.63067200 -2.12695000 4.23044400 L

H-H_ 0 9.25405500 -2.96767600 2.66979800 L

H-H_ 0 0.77885600 -0.70680200 3.07163600 L

H-H_ 0 5.74084200 4.11005800 -4.79581000 L

O-O_R 0 1.92068800 -0.99613200 9.04195300 L

C-C_R 0 8.48361100 4.08185400 -6.81135500 L

C-C_R 0 7.62784500 4.48044100 -5.77874100 L

C-C_R 0 10.19944800 0.97311300 -2.18206500 L

C-C_R 0 1.28952200 0.08906100 4.99840100 L

C-C_R 0 1.35837400 -0.16779800 6.37154000 L

C-C_R 0 10.72916700 0.09718700 -1.21295800 L

C-C_R 0 6.65832900 -1.43615100 4.25175700 L

C-C_R 0 7.98650600 -1.69428000 3.85687300 L

H-H_ 0 9.08966600 3.57964400 -3.75096300 L

H-H_ 0 9.44322000 -3.08156300 5.74185700 L

H-H_ 0 9.39720500 4.63925300 -6.97862600 L

H-H_ 0 8.53053400 2.21226900 -0.95069400 L

H-H_ 0 10.84848200 -2.00263900 3.97052200 L

H-H_ 0 3.87917900 0.26610200 4.84424300 L

O-O_R 0 10.33557400 2.35712100 -2.02341500 L

C-C_R 0 8.66441600 4.31958900 -3.08755400 L

O-O_R 0 7.93945100 5.62806600 -5.03176900 L

H-H_ 0 6.44445500 0.38225900 2.71197800 L

H-H_ 0 1.61525500 0.64204300 7.04340000 L

O-O_R 0 6.29595100 -0.17022900 4.73681000 L

O-O_R 0 1.40360800 1.40471200 4.53541600 L

C-C_3 0 10.16108000 -2.27109600 5.98860100 L

C-C_3 0 9.37961200 2.91024800 -1.10791400 L

C-C_3 0 11.48231600 0.67743400 -0.02327500 L

H-H_ 0 12.43937800 -1.17283100 0.73136500 L

C-C_3 0 9.07416800 -0.70921800 4.24275900 L

C-C_R 0 3.85669300 1.13946000 4.20796200 L

H-H_ 0 13.36763600 -0.30933500 -0.50697900 L

C-C_3 0 10.38035500 -1.37641600 4.75584800 L

C-C_R 0 10.19333300 0.01737200 2.07611800 L

O-O_R 0 8.41005100 -1.66736900 7.71300000 L

H-H_ 0 10.65888600 -0.95241900 2.02382000 L

C-C_3 0 12.72558700 -0.15935700 0.38838900 L

H-H_ 0 11.12173800 -2.76361200 6.25168300 L

C-C_3 0 6.35107600 0.84253800 3.71701300 L

C-C_R 0 8.01625100 5.43695600 -3.64132500 L

C-C_R 0 8.76335500 4.15969600 -1.69426100 L

C-C_R 0 10.49435400 0.98130400 1.09788100 L

C-C_R 0 2.62628200 1.78658400 3.94965000 L

C-C_R 0 9.29272000 0.29134400 3.12067500 L

C-C_R 0 5.05211700 1.61416900 3.62594400 L
H-H_ 0 11.92167300 1.64371100 -0.34963700 L
C-C_R 0 9.68771700 -1.48588500 7.18215100 L
H-H_ 0 8.71790700 -0.12344600 5.11751300 L
C-C_R 0 9.81901700 2.22560900 1.13452000 L
C-C_R 0 8.56596500 1.50735500 3.10152700 L
O-O_R 0 10.03292900 3.19840600 0.13965400 L
H-H_ 0 11.11747400 -0.58305100 5.01086700 L
C-C_R 0 8.87552600 2.48011400 2.14132800 L
C-C_R 0 2.61081800 2.91218800 3.11999100 L
C-C_R 0 8.17233200 5.12026000 -0.85895600 L
C-C_R 0 7.50121500 6.42078100 -2.79179900 L
O-O_R 0 7.46773100 1.72137100 3.96477900 L
C-C_R 0 4.99909600 2.73918000 2.79086500 L
C-C_3 0 13.56240300 0.49474000 1.50295000 L
C-C_R 0 7.53539700 6.25483000 -1.40305500 L
C-C_R 0 3.78603000 3.40710800 2.55321800 L
H-H_ 0 1.67165200 3.41224100 2.91711700 L
H-H_ 0 8.22025000 4.99525700 0.21444300 L
H-H_ 0 8.33044300 3.41479800 2.13589700 L
H-H_ 0 7.03419600 7.30149300 -3.21443200 L
O-O_R 0 10.45675100 -0.64180500 7.71300600 L
H-H_ 0 14.38703700 -0.19275100 1.78828200 L
H-H_ 0 4.98936300 5.49680600 -0.39083900 L
O-O_R 0 15.14762800 1.86506800 0.08943600 L
H-H_ 0 5.89751700 3.07465800 2.29064700 L
H-H_ 0 12.93503500 0.64252600 2.40808500 L
C-C_R 0 14.16190400 1.80605800 1.07446400 L
C-C_R 0 5.33726600 5.88454000 0.55846900 L
O-O_R 0 3.70343500 4.50390300 1.67711100 L
O-O_R 0 6.99116200 7.26709400 -0.59048500 L
C-C_R 0 4.75950600 5.42729000 1.74516800 L
C-C_R 0 6.37490500 6.82920700 0.59141300 L
O-O_R 0 13.78315900 2.87199800 1.62754100 L
C-C_R 0 5.19571800 5.95156700 2.97533900 L
C-C_R 0 6.78811100 7.36224500 1.81638900 L
C-C_R 0 6.22050400 6.91271800 3.02096800 L
H-H_ 0 7.57983800 8.10145800 1.82628800 L

H-H_ 0 4.70210400 5.63375100 3.88442800 L

C-C_R 0 6.68486900 7.47316800 4.31370500 L

O-O_R 0 7.17594500 8.63282600 4.34629100 L

O-O_R 0 6.62846800 6.74157500 5.50242600 L

H-H_ 0 3.51258000 -4.74058600 -6.02156800 L

H-H_ 0 11.37741300 -6.09351600 -4.50655100 L

H-H_ 0 8.69073200 -8.63411700 2.58448700 L

H-H_ 0 8.68074500 0.56517300 -8.53240700 L

H-H_ 0 -0.33806300 -3.07880400 8.38542900 L

H-H_ 0 7.86063400 -2.34420800 7.19718500 L

H-H_ 0 15.40896900 0.94903500 -0.25500700 L

H-H_ 0 6.38997600 5.76731200 5.36861300 L

C-C_R 0 -2.72046400 -0.47153800 -0.03428600 H

C-C_R 0 -3.18888700 -1.78724300 -0.01494600 H

C-C_R 0 -4.55379100 -2.01045800 -0.00807600 H

C-C_R 0 -5.46222300 -0.93734400 -0.01552000 H

C-C_R 0 -4.99255800 0.35882400 -0.04508000 H

C-C_R 0 -3.61093900 0.60763400 -0.05908000 H

H-H_ 0 -2.47817600 -2.60272700 -0.02694500 H

H-H_ 0 -4.91650500 -3.03161700 -0.01072500 H

H-H_ 0 -6.53106100 -1.12051200 -0.01790800 H

H-H_ 0 -5.66259700 1.21009100 -0.06249000 H

C-C_R 0 -3.07654700 1.95862600 -0.11161800 H

C-C_R 0 -1.63443800 2.05436700 -0.18886000 H

C-C_R 0 -0.81886700 0.95803200 -0.13624100 H

C-C_R 0 0.63494500 0.94993100 -0.15085500 H

C-C_R 0 1.36329800 -0.15455700 0.31760500 H

C-C_R 0 1.37176200 2.03671000 -0.65313200 H

C-C_R 0 2.74632600 -0.18174300 0.29419400 H

C-C_R 0 2.75077700 2.01735300 -0.68575800 H

H-H_ 0 0.85670200 2.90352000 -1.03922300 H

C-C_R 0 3.49245000 0.91406800 -0.19996800 H

H-H_ 0 3.24572900 -1.05759800 0.68077100 H

H-H_ 0 3.24921900 2.87415800 -1.11566400 H

O-O_R 0 -3.76104000 2.99041600 -0.08908500 H

O-O_R 0 -1.14497300 3.31648000 -0.27494200 H

H-H_ 0 -1.93630800 3.88368100 -0.26913800 H

O-O_R 0 -1.37186700 -0.29404500 -0.04082100 H

H-H_ 0 0.83127200 -1.01457000 0.70272000 H

N-N_R 0 4.87158000 0.91525700 -0.19999100 H

C-C_3 0 5.58191000 -0.33904000 0.07410800 H

H-H_ 0 5.28275100 -0.69811500 1.06356700 H

H-H_ 0 5.24561500 -1.10356300 -0.63904500 H

C-C_3 0 7.10432100 -0.26020900 0.03984000 H

H-H_ 0 7.51196300 0.40426800 0.79404100 H

H-H_ 0 7.51301400 -1.25525100 0.22736700 H

H-H_ 0 7.49767800 0.08965900 -0.91174800 H

C-C_3 0 5.58369900 2.11228700 -0.62688400 H

H-H_ 0 6.52405000 2.15517300 -0.08453400 H

H-H_ 0 5.02652500 2.97725500 -0.26931600 H

C-C_3 0 5.79946800 2.22683600 -2.13972000 H

H-H_ 0 6.13482600 3.23241100 -2.40374300 H

H-H_ 0 6.54636400 1.51680500 -2.50111400 H

H-H_ 0 4.87089900 2.03731900 -2.68117700 H
